# Synthesis and Evaluation of Trehalose‐Based Mertansine Warheads for Bacillus Calmette–Guérin Delivery of Anticancer Agents

**DOI:** 10.1002/cbic.202500390

**Published:** 2025-07-09

**Authors:** Michael Grimmeisen, Xuan Wang, Melissa Weldle, Kerstin Sartory, Sara Benkhelifa, Yu Zhang, Trinh Dao, Jonas Meyer, Oliver Gorka, Olaf Groß, Claudia Jessen‐Trefzer

**Affiliations:** ^1^ University of Freiburg Institute of Organic Chemistry Albertstrasse 21 79104 Freiburg Germany; ^2^ Zhongshan Institute for Drug Discovery Shanghai Institute of Materia Medica Chinese Academy of Sciences Zhongshan 528400 China; ^3^ CIBSS‐Centre for Integrative Biological Signalling Studies University of Freiburg Schänzlestrasse 18 79104 Freiburg Germany; ^4^ Institute of Neuropathology, University of Freiburg University Medical Center Faculty of Medicine Breisacher Straße 113 79106 Freiburg Germany

**Keywords:** antigen 85 complex, anti cancer agents, bladder cancer, delivery, mertansine, mycobacteria, trehalose

## Abstract

Nonmuscle invasive bladder cancer (NMIBC) accounts for 75% of bladder cancer cases, with Bacillus Calmette–Guérin (BCG) immunotherapy as the gold standard for high‐risk patients. BCG elicits a robust immune response but is limited by adverse effects and resistance. To enhance its efficacy, a trehalose‐based conjugation strategy is developed, tethering a cytostatic agent to BCG via a cleavable disulfide linker. This system enables selective drug integration into the BCG envelope and controlled release in tumor cells, aiming to improve therapeutic precision while minimizing toxicity. This approach combines immunotherapy with targeted chemotherapy, offering a promising strategy for NMIBC treatment.

## Graphical abstract

Bacillus Calmette–Guérin (BCG) immunotherapy is the gold standard for nonmuscle invasive bladder cancer (NMIBC). However, it is limited by adverse effects and resistance, despite eliciting a robust immune response. This proof‐of‐concept study explores Antigen 85‐mediated, metabolically with trehalose‐PEG_4_ mertansine‐labeled BCG as a novel treatment option for NMIBC, offering promising *in cellulo* results.

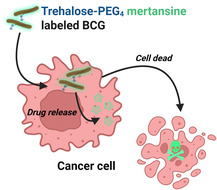

## 1 Introduction

Bladder cancer is one of the most common malignancies worldwide, with nonmuscle invasive bladder cancer (NMIBC) accounting for ≈75% of newly diagnosed cases.^[^ ^1^ ^]^ The standard treatment for early‐stage bladder cancer involves transurethral resection of the tumor, often followed by adjuvant therapies to reduce the risk of recurrence and progression. Among the available treatment options, intravesical Bacillus Calmette–Guérin (BCG) immunotherapy has emerged as the most effective and widely utilized approach for patients with high‐risk NMIBC.^[^ ^1^, ^2^, ^3^, ^4^ ^]^ BCG, an attenuated strain of *Mycobacterium bovis (M. bovis)*, was originally developed as a vaccine against tuberculosis but has been repurposed as a potent immunotherapeutic agent for bladder cancer.^[^ ^5^, ^6^, ^7^, ^8^ ^]^ Administered directly into the bladder through intravesical instillation, BCG induces a robust local immune response, which plays a critical role in eradicating residual tumor cells and preventing recurrence. The mechanism of action involves the activation of both innate and adaptive immunity. BCG triggers a pro‐inflammatory response by stimulating urothelial cells and resident immune cells, leading to the recruitment of macrophages, neutrophils, and T lymphocytes to the bladder wall. This immune activation results in the production of cytokines, such as interleukin‐2 (IL‐2), tumor necrosis factor‐alpha (TNF‐α), and interferon‐gamma (IFN‐*γ*), which mediate cytotoxic effects against tumor cells. Furthermore, when BCG is exposed to the tumor microenvironment, it adheres to the cell surface and is internalized by tumor cells, activating various signaling pathways, including nuclear factor‐kappa B (NF‐κB). This process triggers the release of cytokines by immune cells such as neutrophils and macrophages, which contribute to the immune cascade or directly target tumor cells for destruction. However, the exact mechanisms underlying these effects are complex and not yet fully understood.^[^ ^6^, ^7^, ^8^, ^9^, ^10^ ^]^

BCG therapy remains the gold standard for adjuvant treatment of nonmuscle‐invasive bladder cancer (NMIBC), particularly in carcinoma in situ (CIS) and high‐grade tumors, due to its proven efficacy in reducing recurrence and delaying progression.^[^ ^3^, ^9^, ^10^, ^11^ ^]^ However, adverse effects such as local inflammation, urinary symptoms, and rare systemic infections have prompted exploration of alternatives. Intravesical chemotherapies, including mitomycin C, gemcitabine, epirubicin, and docetaxel, have shown variable efficacy, with combinations like gemcitabine‐docetaxel demonstrating promise. Immunotherapeutic options include IFN‐α with BCG and checkpoint inhibitors (e.g., Atezolizumab, Pembrolizumab) for BCG‐unresponsive cases. Emerging therapies, such as gene therapy (e.g., Nadofaragene Firadenovec) and FGFR inhibitors, offer additional avenues, particularly for high‐risk or refractory patients.^[^ ^12^ ^]^ Surgical options, including TURBT and early radical cystectomy, remain vital in select cases.

Recent research focuses on enhancing BCG's efficacy and safety. Strategies include conjugating BCG with therapeutic agents (e.g., doxorubicin‐loaded nanoparticles) and genetically modifying BCG to boost immunogenicity.^[^ ^13^, ^14^, ^15^ ^]^ Engineered strains overexpressing cyclic di‐AMP^[^ ^16^ ^]^ or IL‐15‐Ag85B have shown improved immune activation and antitumor effects.^[^ ^17^ ^]^ Novel delivery systems—such as magnetic chitosan hydrogels and cationic nanoparticles—have further enhanced BCG retention and immune responses in preclinical models.^[^ ^18^ ^]^ These innovations are undergoing clinical evaluation, with future trials essential to determine their translational potential in NMIBC management.

**Figure 1:**
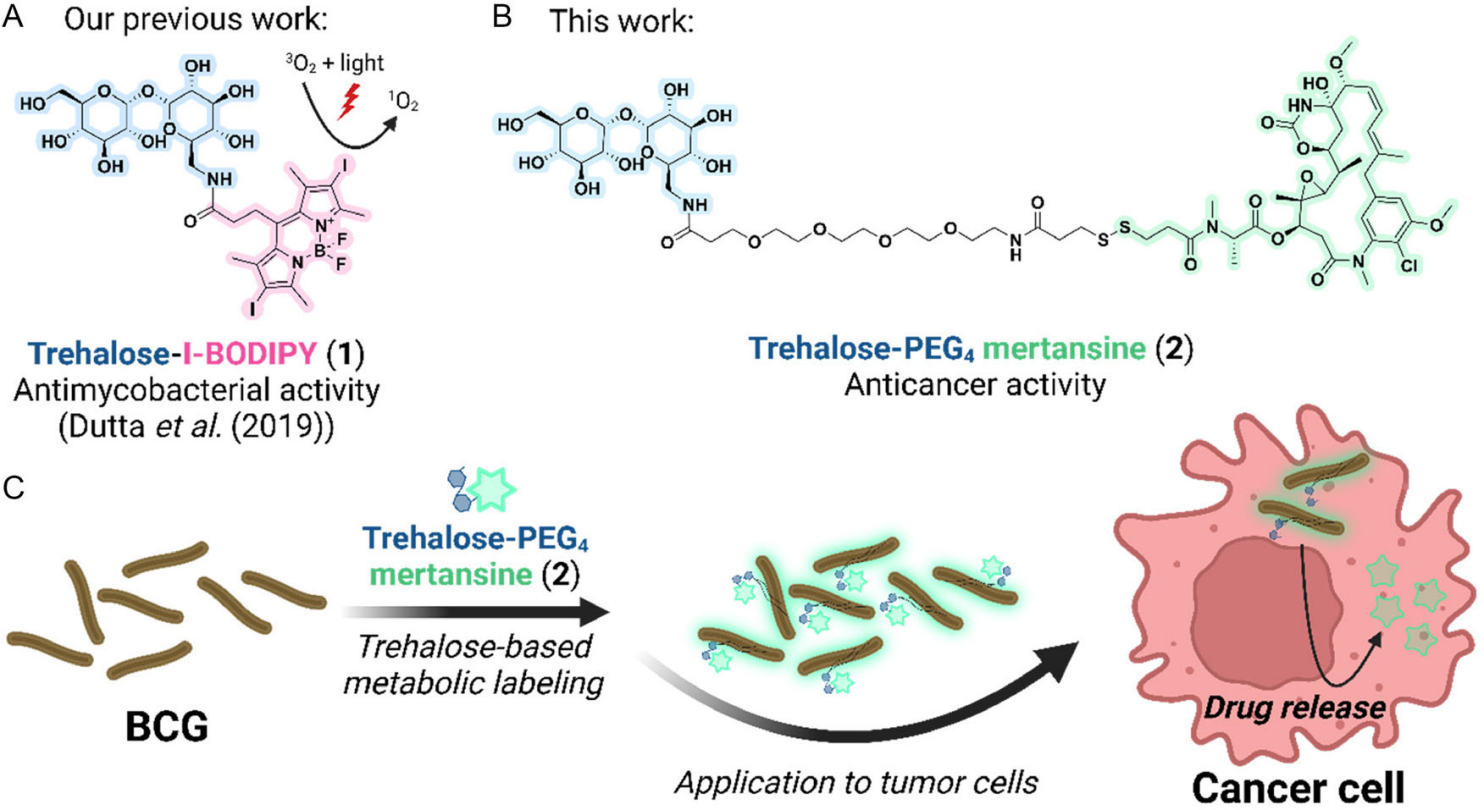
Trehalose‐photosensitizer conjugates as light‐triggered antimycobacterial agents and the described novel trehalose‐mertansine conjugate as anticancer agent. A) Trehalose‐I‐BODIPY (**1**). B) Trehalose‐PEG_4_ mertansine (**2**). C) Working hypothesis of the described trehalose‐mertansine conjugate **2**. Created in BioRender. Grimmeisen, M. (2025) https://BioRender.com/f45f537, and ChemDraw 23.

In this manuscript, we expand on our previous work, in which we described the synthesis of trehalose‐BODIPY conjugates as antimycobacterial agents (**Figure** **1**A). Here, we report the chemical synthesis and evaluation of a cytostatic agent conjugated to BCG, utilizing the disaccharide trehalose as a molecular scaffold (Figure 1B).

**Figure 2:**
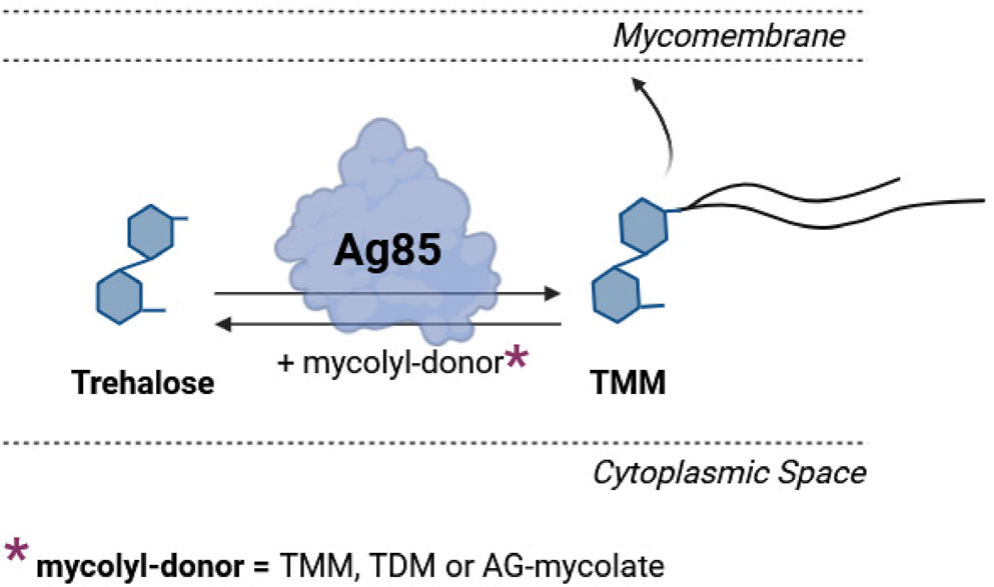
Schematic representation of the Ag85 antigen complex and the catalyzed esterification of trehalose to TMM. TMM is subsequently incorporated into the outer membrane. Mycolyl‐donors for the outlined reaction are TMM, TDM, or arabinogalactan‐mycolates (AG‐mycolates). Mycolic acids are structurally diverse long‐chain fatty acids and contain between 60 and 90 carbon atoms. Created in BioRender. Grimmeisen, M. (2025) https://BioRender.com/l01p077.

The cytostatic agent mertansine^[^ ^19^, ^20^, ^21^ ^]^ is tethered to trehalose through a cleavable disulfide linker moiety, enabling controlled drug release. Trehalose, a nonreducing disaccharide, is an ideal scaffold due to its natural interaction with mycobacterial species. Specifically, trehalose and its analogs are efficiently incorporated into the BCG cell envelope via the Antigen 85 (Ag85) enzyme complex, a family of mycolyl‐transferases crucial for mycobacterial cell wall biosynthesis. The Ag85 enzyme complex catalyzes the transfer of mycolic acids—long‐chain fatty acids—which are essential glycolipids in the mycobacterial outer membrane. The Ag85 isoforms (Ag85A, Ag85B, and Ag85C) facilitate a reversible transesterification reaction in which two trehalose monomycolate (TMM) molecules interact to produce trehalose dimycolate (TDM) and free trehalose. Additionally, the reverse process enables the direct esterification of trehalose (**Figure** **2**). These lipids contribute to the membrane's structural integrity, impermeability, and virulence, allowing mycobacteria to survive in hostile environments. By exploiting this natural incorporation pathway, trehalose‐based conjugates are selectively recognized and processed by Ag85, facilitating their integration into the BCG outer membrane.^[^ ^22^, ^23^ ^]^

While these trehalose‐based conjugates improve incorporation into the mycobacterial cell wall via Ag85, our design further enhances their functionality by including a cleavable disulfide linker, which introduces a key functional advantage. Disulfide bonds are sensitive to the reducing intracellular environment, particularly within the cytoplasm, where elevated levels of glutathione (GSH) and other reducing agents trigger bond cleavage.^[^ ^24^ ^]^ After integration of the trehalose‐cytostatic conjugate into the BCG cell envelope via Ag85‐mediated transfer, the bacterial conjugate can be applied to bladder cancer cells. The reducing conditions within these cells should promote disulfide bond cleavage, releasing the active cytostatic payload from the bacteria specifically at the target site (Figure 1C). This mechanism ensures precise, controlled drug release, minimizing off‐target effects while enhancing therapeutic efficacy. By harnessing the natural function of the Ag85 enzyme complex and incorporating cleavable disulfide linkers, our approach provides a targeted delivery system for cytostatic agents. This strategy shows significant promise for developing innovative therapies for early‐stage bladder cancer. By combining the immune‐stimulating properties of BCG with targeted drug release, this method has the potential to improve treatment outcomes, reduce cancer recurrence, and offer a less invasive alternative to systemic chemotherapy or other conventional therapies.

## 2 Results and Discussion

In 2019, our group reported the synthesis and incorporation of trehalose‐photosensitizer conjugates (see Figure 1A, Trehalose‐I‐BODIPY (**1**)) for light‐triggered killing of mycobacterial species, including *Mycobacterium tuberculosis* (*M. tuberculosis*) and *Mycobacterium abscessus* (*M. abscessus*).^[^ ^25^ ^]^ This work was inspired by earlier studies from the B. Davis group, who demonstrated the incorporation of fluorescent trehalose reporters into the mycobacterial membrane via the Ag85 enzyme complex. In the last years, similar fluorescent trehalose reporters have been successfully incorporated into the membranes of various mycobacterial strains, including *M. bovis*, *Mycobacterium smegmatis* (*M. smegmatis*), *Mycobacterium marinum* (*M. marinum*), and *M. tuberculosis*.^[^ ^22^, ^26^, ^27^ ^]^ To explore whether a cytostatic trehalose probe could be used to label the outer membrane of BCG, we first tested the toxicity of the cytostatic agent alone against BCG. For our experiments, we focused on the clinically approved drug mertansine. Mertansine (DM1) is a potent cytotoxic agent used in antibody‐drug conjugates (ADCs) for targeted cancer therapy. It disrupts microtubule assembly by binding to tubulin, leading to cell cycle arrest and apoptosis.^[^ ^19^, ^20^, ^21^ ^]^ The minimal inhibitory concentration of mertansine against BCG was >200 μM, as determined using a resazurin‐reduction assay (Figure S1, Supporting Information), enabling us to proceed with our study.

### 2.1 Synthesis and Characterization of Trehalose‐Fluorophore Conjugates

**Figure 3:**
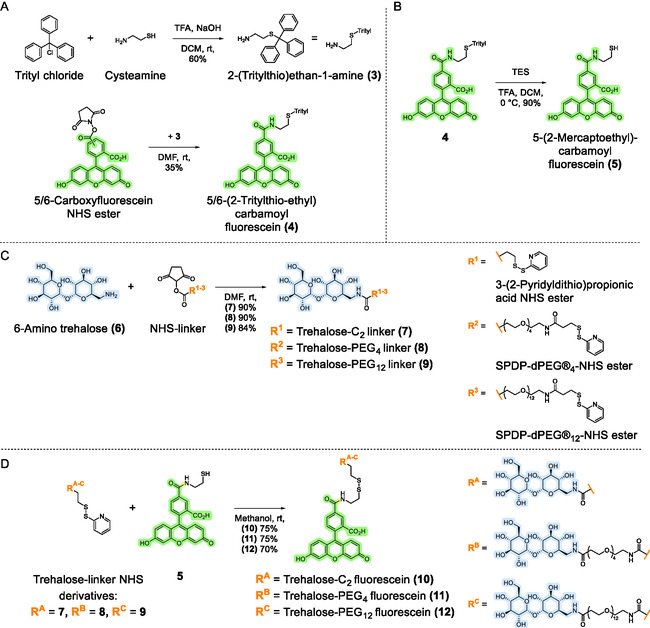
Synthesis procedure for generation of the described trehalose‐fluorescein conjugates. A) The trityl‐functionalized fluorescein derivative **4**. B) Thiol‐modified fluorescein derivative **5**. C) Trehalose‐linker derivatives **7**, **8**, and **9**. D) The final products trehalose‐C_2_ fluorescein (**10**), trehalose‐PEG_4_ fluorescein (**11**), and trehalose‐PEG_12_ fluorescein (**12**). Created with ChemDraw 23.

Next, we focused on identifying the optimal construct, particularly the ideal linker length, for connecting trehalose to the cytotoxic agent via a cleavable disulfide bridge. We began by synthesizing trehalose‐fluorophore conjugates—as fluorescent probes to simplify initial validations—using different linkers for subsequent in vitro release studies of the conjugated fluorophore in the presence of a reducing agent. The fluorophore 5‐(2‐mercaptoethyl)‐carbamoyl fluorescein (**5**) was synthesized from a mixture of 5‐ and 6‐carboxyfluorescein NHS esters using a slightly modified procedure from Togashi et al., with a yield of 19% over three steps (**Figure** **3** and Supporting Information). Concurrently, 6‐amino trehalose was coupled to three different linkers: I) 3‐(2‐pyridyldithio)propionic acid NHS ester, II) SPDP‐dPEG4‐NHS ester, and III) SPDP‐dPEG12‐NHS ester. The resulting trehalose‐linker‐disulfide intermediates were then reacted with **5** to yield the final trehalose‐fluorescein compounds: **10** (C_2_), **11** (PEG_4_), and **12** (PEG_12_) with yields of 68%, 68%, and 59% over two steps, respectively.

### 2.2 Lipid Analysis of Incorporated Trehalose‐Fluorophore Conjugates

**Figure 4:**
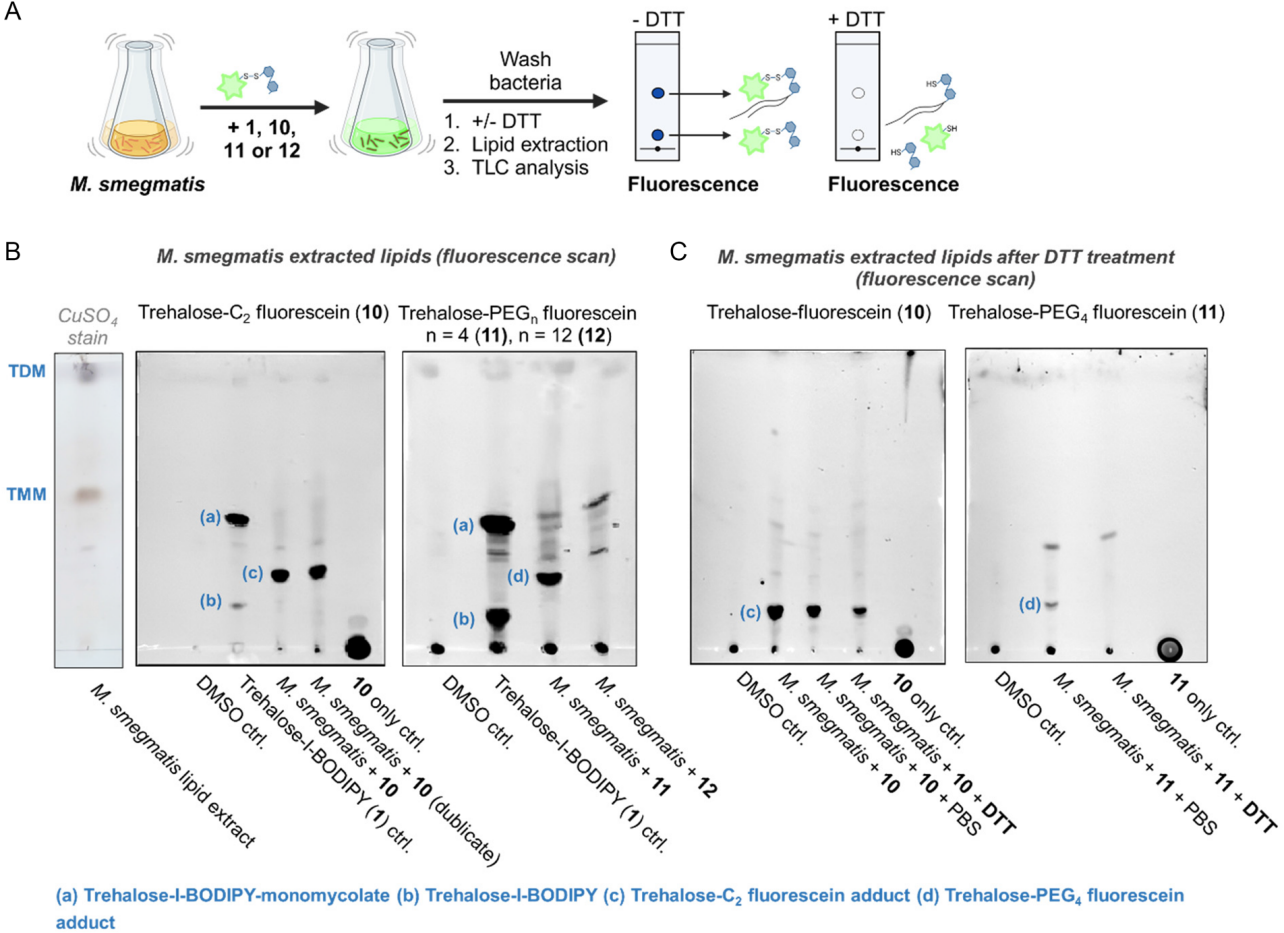
Lipid analysis of *M. smegmatis* treated with trehalose conjugates. Trehalose‐I‐BODIPY (**1**) was used as a control. *M. smegmatis* was treated with the indicated trehalose conjugate (10 μM) for 6 h. After lipid extraction, the samples were resolved by TLC in chloroform/methanol/water (20:4:0.5). TLC plates were scanned for fluorescence (emission (Em) = 590 nm) or stained by copper sulfate (CuSO_4_). A) Simplified scheme of the assay workflow. B) CuSO_4_ stained analysis of wild‐type *M. smegmatis* lipids as control to visualize retention factors (Rf) of natural TMM and TDM. Additionally, analysis of trehalose‐C_2_ fluorescein (**10**), trehalose‐PEG_4_ fluorescein (**11**), and trehalose‐PEG_12_ fluorescein (**12**). C) To test whether conjugated fluorophores could be cleaved by reduction, *M. smegmatis* was incubated with 100 μM DTT (or PBS as a control) after pretreatment with the respective trehalose conjugate to break the disulfide bond. Lipids were extracted after 30 min of incubation and analyzed. Created in BioRender. Grimmeisen, M. (2025) https://BioRender.com/d43r257.

These three conjugates were subsequently tested for incorporation into *M. smegmatis* to evaluate the effect of linker length on membrane integration. *M. smegmatis* is often used as a surrogate for BCG and other mycobacteria in preclinical studies due to its genetic similarity, faster growth, and safer handling. Furthermore, metabolic labeling with C^6^‐OH modified‐trehalose analogs was shown to rely on conserved pathways in *M. smegmatis* and BCG.^[^ ^22^, ^27^, ^28^ ^]^ To this end, *M. smegmatis* was cultured in standard 7H9 broth until it reached an optical density (OD) of 0.7. The cells were then harvested by centrifugation and washed with fresh culture broth. Compounds **10**, **11**, and **12** were each added to separate vessels containing *M. smegmatis* culture at an adjusted OD of 0.1. After 6 h of incubation, the bacterial cells were collected by centrifugation and washed twice with ultrapure water. Following our published protocol,^[^ ^25^ ^]^ bacterial membrane lipids were extracted using chloroform/methanol, and the isolated lipids were analyzed by thin‐layer chromatography (TLC, **Figure** **4**A) in a solvent system of chloroform/methanol/water (20:4:0.5).

Fluorescently labeled lipids were visualized by fluorescence scanning on a gel imager with an emission wavelength of 590 nm. As a positive control, we used the trehalose‐I‐BODIPY conjugate **1**, previously synthesized and validated in our group.^[^ ^25^ ^]^ In our reported study, **1** was shown to be modified by mycolic acids upon incubation with mycobacteria applying the same workflow, and lipid extraction yielded trehalose‐I‐BODIPY‐mycolate (Figure 4, TLC spot (a): Rf = 0.43). Repeating the analysis with **10**, **11**, and **12** gave only for **10** and **11** a clear spot of the mycolate adduct (Figure 4, TLC spot (c): Rf = 0.25 and (d): Rf = 0.23). We figured that compound **12** potentially failed to incorporate into mycobacterial membranes, whereas compounds **10** and **11** demonstrated successful incorporation (Figure 4B). This suggests that the linker SPDP‐dPEG12‐NHS ester may be too long, preventing the conjugate from accessing the periplasmic space to interact with Ag85, or that Ag85 does not recognize the molecule as a substrate due to steric hindrance at its active site. Next, we aimed to verify whether the fluorophore of the incorporated compounds **10** and **11** could be cleaved from the membrane in the presence of a reducing agent, hence if the fluorophore was accessible and not buried within the membrane. After a 6 h incubation of the bacteria with **10** or **11** and subsequent washing, dithiothreitol (DTT) was added to reduce disulfide bonds and release the fluorophore from the trehalose conjugate. Subsequently, lipids were isolated and analyzed by TLC. As shown in Figure 4C, only the fluorophore from modified **11** (TLC spot (d)) was released, whereas modified **10** (TLC spot (c)), carrying the shorter C_2_ linker, remained at least partially intact. These results indicate that the trehalose‐fluorescein conjugate **11** was successfully processed and incorporated into the *M. smegmatis* membrane, with the fluorophore accessible for subsequent reduction and release. Consequently, linker **8** was found suitable for further synthesis of trehalose conjugates designed to deliver cleavable cytotoxic agents.

In the next experiment, we aimed to quantify the average amount of incorporated conjugate per colony‐forming unit (CFU). CFU is a standard measure commonly used to describe bacterial load in experiments, including those involving bladder instillation.^[^ ^29^ ^]^ We incubated the fluorescent trehalose‐BODIPY probe **13**,^[^ ^25^ ^]^ which lacks a cleavable linker, in 1 mL of bacterial culture at an OD of 0.1 containing 1.5 × 10^7^ CFU. After a 24 h incubation, the bacteria were washed twice with PBS containing 10% DMSO to remove nonspecifically bound probe. Membrane lipids were then isolated, and the amount of BODIPY‐containing lipids was quantified via fluorescence measurement against a standard curve generated with compound **13** (Figure S2, Supporting Information). Using this approach, we determined that an average of 395 fmol per 10^6^ CFU corresponding to 5.85 pmol of compound **13** per mL BCG at an OD 0.1 was incorporated into the BCG membrane lipids. This value was subsequently employed in control experiments to assess the activity of BCG in combination with the nonconjugated drug against bladder cancer cells.

### 2.3 Synthesis and Characterization of the Trehalose‐Warhead Conjugate

**Figure 5:**
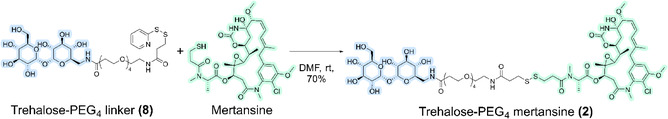
Synthesis procedure of trehalose‐PEG_4_ mertansine (**2**). Created with ChemDraw 23.

Next, we synthesized the trehalose conjugate **2,** carrying the cytotoxic agent mertansine (**Figure** **5**). For the synthesis of trehalose‐PEG_4_ mertansine (**2**), we utilized trehalose‐PEG_4_ linker **8**, which was subsequently coupled to mertansine, a compound with a naturally occurring free thiol group. The final product, trehalose‐PEG_4_ mertansine (**2**), was obtained in a yield of 16% over 5 steps (starting from d‐(+)‐trehalose, see Supporting Information).

To confirm the successful release of the cytotoxic agent in vitro via disulfide bridge reduction, trehalose‐PEG_4_ mertansine (**2**) was incubated with 10 mM DTT and analyzed by high‐pressure liquid chromatography (HPLC). After 30 min of incubation, complete release of mertansine was observed (Figure S3, Supporting Information). Additionally, the toxicity of the compound was evaluated to determine the activity against BCG. Trehalose‐PEG_4_‐mertansine (**2**) exhibited a MIC > 200 μM against BCG after 24 h of incubation, highlighting its potential as a warhead for further investigation (Figure S1, Supporting Information).

### 2.4 In Vitro Ag85 Processing Assay

The substrate specificity and a full kinetic analysis of the Ag85 enzymes have been previously reported by Backus and coworkers using a ^14^C‐trehalose radioassay that monitors ^14^C‐trehalose radiolabel incorporation into TDM and TMM by thin‐layer chromatography and a complementary mass spectrometry‐based assay.^[^ ^22^ ^]^ Using this assay, it was demonstrated that mono‐ and dihexanoyl trehalose substrates, which proved to be water soluble, are readily processed by Ag85 as alkyl donors. To investigate whether **2** is recognized by the Ag85 enzymes in vitro, we monitored the enzymatic reaction of the complex members Ag85A and B individually in the presence of trehalose‐hexadecanoate and **2**. The enzymes were purified according to published procedures.^[^ ^23^, ^30^ ^]^ Control compound was trehalose‐I‐BODIPY (**1**) (Figure S4, Supporting Information). From the assay, we inferred that trehalose‐PEG_4_ mertansine (**2**) serves as a substrate of Ag85 enzymes and is successfully modified with hexadecanoate derived from trehalose‐hexadecanoate.

### 2.5 Cellular Assays Using Human Bladder Cancer Cell Lines HT‐1376, T24, and mouse BMDMs

Next, the IC_50_ values (inhibitory concentrations) of mertansine and trehalose‐PEG_4_ mertansine (**2**) were determined for HT‐1376 and T24 cell lines (**Table** **1** and Figure S5, Supporting Information). HT‐1376 and T24 are human bladder cancer cell lines commonly used in research to study bladder cancer biology, drug responses, and molecular mechanisms of tumor progression.^[^ ^31^ ^]^ Blasticidin S was used as a positive control. Mertansine exhibited IC_50_ values in the nanomolar to low micromolar range against both cell lines, with T24 cells showing slightly higher susceptibility to it. Trehalose‐PEG_4_ mertansine (**2**) exhibited a higher toxicity against both cell lines. This increase in toxicity might be attributed to the presence of polyethylene glycol (PEG) in **2**. PEGylation of cytotoxic agents has already been reported in the literature to potentially increase cytotoxicity. PEGylation enhances cytotoxic drug efficacy by improving aqueous solubility (e.g., 20‐fold increase in paclitaxel solubility via PEG 400) and stability through steric shielding against degradation.^[^ ^32^ ^]^ Clinical studies demonstrate PEGylated formulations (e.g., Doxil reduce systemic toxicity: 90% lower cardiotoxicity versus free doxorubicin^[^ ^33^ ^]^) and prolong circulation (77‐h half‐life for PEG‐camptothecin^[^ ^34^ ^]^), validating its role in optimizing therapeutic indices for bladder cancer drug delivery. Along the same lines, it was shown that the efficacy of the photosensitizer 5,10,15,20‐tetrakis(4‐hydroxyphenyl)porphyrin (p‐THPP) could be increased by PEGylation.^[^ ^35^ ^]^

**Table 1:** IC_50_ values of the two cell lines upon treatment with mertansine and trehalose‐PEG_4_ mertansine(2).

	IC_50_ [HT‐1376]	IC_50_ [T24]
Blasticidin S (positive control)	1.9 ± 2.3 μM	5.3 ± 0.6 μM
Mertansine	2.7 ± 2.2 μM	0.31 ± 0.30 μM
Trehalose‐PEG_4_ mertansine (**2**)	0.05 ± 0.16 μM	0.15 ± 0.02 μM

Cell viability was assessed using the 3‐[4,5‐dimethylthiazol‐2‐yl]‐2,5 diphenyl tetrazolium bromide (MTT) reduction assay in 96‐well plate format following a 24‐h incubation period, followed by two washing steps with PBS and an additional 48 h incubation period. During the 24‐h incubation period, trehalose conjugate **2** remained stable in the extracellular medium, suggesting that the cytotoxic effect likely arises from its ability to enter the cells and release the active drug in the presence of intracellular thiols. The compound's stability in cell culture medium was assessed using HPLC analysis (Figure S6, Supporting Information). Over 24 h, it showed no significant degradation in both supplemented EMEM and supplemented DMEM/F12 media, maintaining a purity of ≥98%.

**Figure 6:**
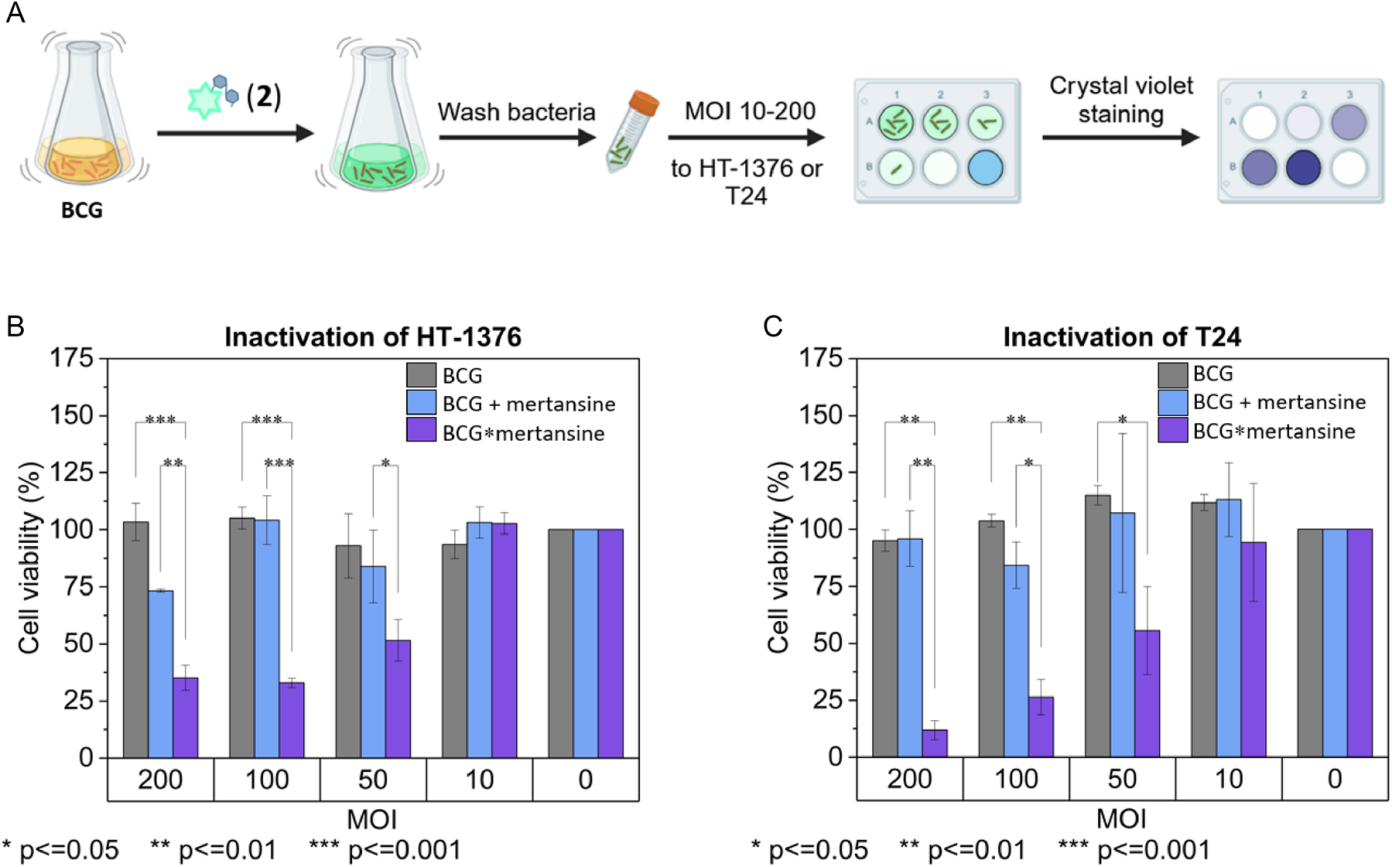
Comparison of the cell viability of bladder cancer cells treated with wild‐type BCG, BCG*mertansine, and BCG combined with nonconjugated mertansine (BCG + mertansine). A) Scheme of the assay workflow. B) Cell viability of HT‐1376 cells. C) Cell viability of T24 cells. Data were normalized to the untreated control. Experiments were performed at least in triplicates. Created in BioRender. Grimmeisen, M. (2025) https://BioRender.com/w24r975 and Origin Pro 2025.

In the next experiment, we used the synthesized trehalose conjugate **2** to label the BCG envelope, following the same protocol as for trehalose‐fluorophore conjugates. The labeled bacteria BCG* mertansine were applied to the bladder cancer cell lines HT‐1376 and T24 at varying multiplicities of infection (MOIs) ranging from 10 to 200. Bladder cancer cells were seeded in 6‐well plates and incubated overnight for attachment before bacterial treatment. After a 24 h incubation with BCG*mertansine, the cells were washed and incubated for another 48 h. Live cells were then stained with crystal violet. The plates were imaged, and cell viability was quantified by solubilizing the crystal violet stain with ethanol, followed by absorbance measurement against cellular standards (**Figure** **6**). Controls included untreated cells, nonlabeled BCG, and BCG combined with nonconjugated drug at concentrations matching the average labeling efficiency as determined earlier using the trehalose‐fluorophore conjugate **13** at different MOIs. For HT‐1376 cells (120,000 cells seeded), drug concentrations of 9.5 ± 0.6 pmol per well, 4.7 ± 0.4 pmol per well, 2.37 ± 0.14 pmol per well, and 0.47 ± 0.03 pmol per well were applied at MOIs of 200, 100, 50, and 10, respectively. For T24 cells (33,000 cells seeded), MOIs of 200, 100, 50, and 10 corresponded to drug concentrations of 2.6 ± 0.2 pmol per well, 1.30 ± 0.08 pmol per well, 0.65 ± 0.04 pmol per well, and 0.130 ± 0.008 pmol per well, respectively. All measurements were performed at least in triplicates and showed high reproducibility.

Experiments on HT‐1376 and T24 cells showed consistent trends. No reduction in cell viability was observed with wild‐type BCG treatment at various MOIs. This finding aligns with previous reports, including those by Ratliff and colleagues, who demonstrated that the efficacy of BCG treatment depends on cytokine release, the recruitment of immune cells—particularly T cells—and the subsequent induction of apoptosis in bladder cancer cells.^[^ ^36^ ^]^ Furthermore, treatment outcomes are heavily influenced by the BCG subtype, which is classified into substrains such as Russia, Japan, Moreau, Danish, Glaxo, Tice, Connaught, Pasteur, and Phipps, each associated with variable experimental and clinical responses in bladder cancer therapy.^[^ ^37^ ^]^

BCG*mertansine demonstrated significant cytotoxicity in HT‐1376 cells, reducing cell viability to 51% ± 6% at an MOI of 50, 32.9% ± 1.4% at 100, and 35% ± 4% at 200, with no observable reduction at an MOI of 10. In comparison, treatment with BCG combined with nonconjugated mertansine only showed a notable reduction in viability to 73.1% ± 0.5% at an MOI of 200. It is noteworthy that the applied concentrations of nonconjugated mertansine are well below its determined IC_50_ value. Overall, these observations suggest that BCG*mertansine may have an advanced toxic effect, probably facilitated by BCG internalization into cancer cells and subsequent intracellular release of the active drug. Alternatively, it is conceivable that the chemical modification of BCG with trehalose conjugates alters its membrane composition, enhancing its ability to enter cells and exert cytotoxic effects. Further investigation is needed to clarify this mechanism. In T24 cells, BCG*mertansine exhibited even greater cytotoxicity, reducing cell viability to 94% ± 18% at an MOI of 10, 55% ± 13% at 50, 26% ± 5% at 100, and 12% ± 3% at 200. This cytotoxicity was significantly higher than that observed with BCG combined with nonconjugated mertansine (e.g., 96% ± 9% at an MOI of 200), underscoring the enhanced efficacy of the BCG*mertansine conjugate in reducing cell viability, particularly in the T24 bladder cancer cell line, as shown in Figure 6.

**Figure 7:**
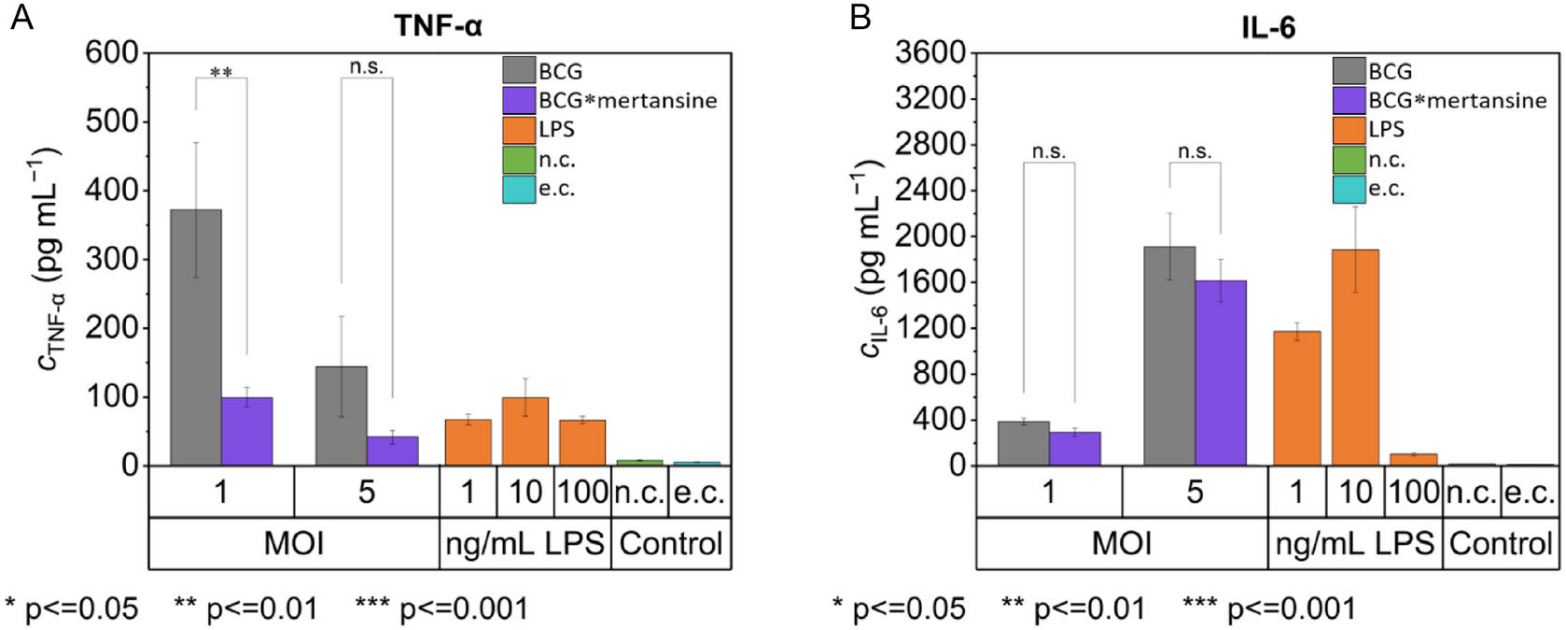
Comparison of cytokine release from BMDMs after incubation with wild‐type BCG and BCG*mertansine at MOI 5 and MOI 1 and the results of the controls, LPS (1, 10, 100 ng mL^−1^), negative control (n.c., medium only), and ELISA control (e.c., background of the ELISA assay). A) The determined TNF‐α concentrations. B) The measured IL‐6 concentrations.

In addition to our cytotoxicity studies, we performed an Enzyme‐Linked Immunosorbent Assay (ELISA) to analyze cytokine secretion from mouse bone marrow‐derived dendritic cells (BMDMs^[^ ^38^ ^]^) stimulated with either wild‐type BCG or BCG*mertansine. Cytokine release was reduced by modification of BCG with trehalose‐PEG_4_ mertansine compared to wild‐type BCG, but was still present (**Figure** **7** and S7, Supporting Information and **Table** **2**). Lipopolysaccharide (LPS) was used as positive control. Supernatants from stimulated BMDMs showed that BCG*mertansine induced 29.0% and 26.8% of the TNF‐α response observed with wild‐type BCG at MOIs of 5 and 1, respectively. For interleukin‐6 (IL‐6), BCG*mertansine was found to have an ELISA response of 84.5% at MOI 5 and 75.2% at MOI 1, compared to wild‐type BCG.

**Table 2:** Cytokine release (TNF‐α and IL‐6) of BMDMs after stimulation with either wild‐type BCG or BCG*mertansine.

	MOI	TNF‐α [pg mL^−1^]	IL‐6 [pg mL^−1^]
Wild‐type BCG	1	372 ± 80	387 ± 25
BCG*mertansine		100 ± 11	291 ± 29
Wild‐type BCG	5	145 ± 60	1913 ± 237
BCG*mertansine		42 ± 8	1615 ± 151

The consistency in IL‐6 production between the wild‐type and mertansine‐modified BCG suggests that basic inflammatory signaling pathways remain intact in BCG*mertansine.^[^ ^39^ ^]^ The reduced TNF‐alpha secretion in BCG*mertansine represents a potentially significant finding with complex implications. The reduction in TNF‐α production could potentially compromise antitumor activity, given this cytokine's established importance in BCG's therapeutic mechanism. Interestingly, excessive inflammatory responses may not always correlate with improved treatment outcomes.^[^ ^40^ ^]^ While TNF‐α is clearly important, its reduced expression might potentially improve tolerability without sacrificing efficacy if other cytotoxic mechanisms remain intact. Whether this change ultimately enhances or diminishes therapeutic efficacy requires further investigation, as the optimal balance of inflammatory stimulation remains an area of active research in BCG immunotherapy for bladder cancer.

**Figure 8:**
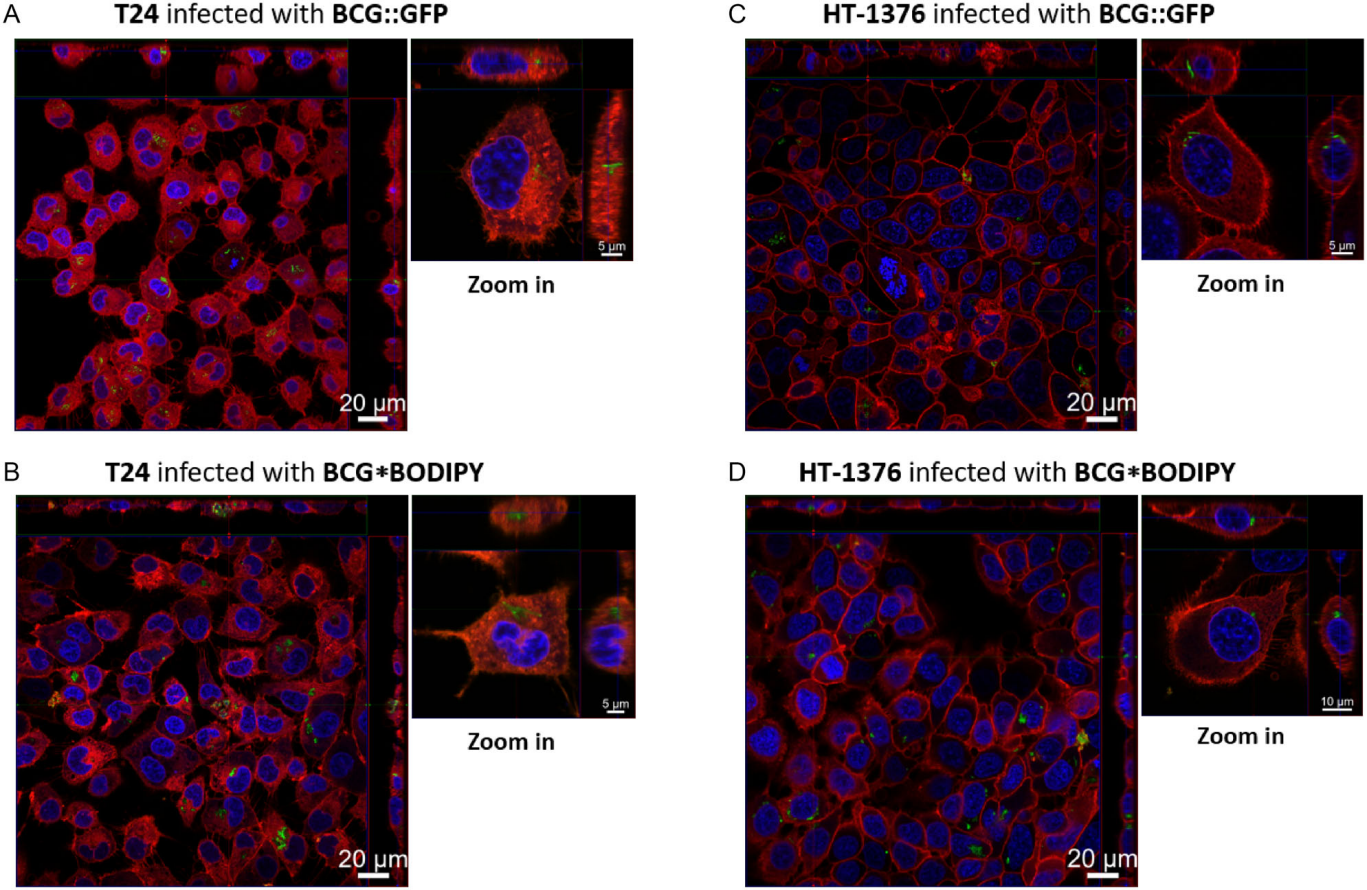
Fluorescence microscopy of bladder cancer cells infected with BCG:GFP and BCG*BODIPY. A) T24 cells infected with BCG:GFP at a MOI of 100, overview and single cell z‐stack. B) T24 cell infected with BCG*BODIPY at a MOI of 100, overview and single cell z‐stack. C) HT‐1376 cells infected with BCG:GFP at a MOI of 200, overview and single cell z‐stack. D) HT‐1376 cell infected with BCG*BODIPY at a MOI of 200, overview and single cell z‐stack. Nucleus was stained with 4′,6‐diamidino‐2‐phenylindol (DAPI) and the cell membrane with CellMask Orange.

To assess whether BCG labeled with our trehalose conjugates was successfully internalized, we utilized confocal fluorescence microscopy on HT‐1376 and T24 cell lines using BCG* BODIPY (labeled with **13**). As controls, we included BCG expressing green fluorescent protein (BCG:GFP). While the general ability of wild‐type BCG to be internalized by bladder cancer and normal bladder epithelial cells has been extensively studied, it remains incompletely understood. In vitro studies suggest that bladder cancer cell lines internalize BCG through phagocytosis and endocytosis, with less differentiated cells exhibiting higher uptake. Electron microscopy studies in animal models indicate that normal intact urothelial cells are unable to internalize BCG, potentially due to differences in the extracellular matrix and altered fibronectin expression in cancerous and regenerating urothelial cells.^[^ ^4^, ^9^, ^41^ ^]^ Our confocal microscopy images and z‐stacks clearly demonstrate that BCG*BODIPY, as well as BCG:GFP, are internalized by bladder cancer cells under the applied conditions (**Figure** **8**). These findings align with previous studies by Backus and coworkers,^[^ ^22^ ^]^ which showed successful internalization of fluorophore‐modified BCG into macrophages.

Thus, we conclude that trehalose conjugation does not affect the ability of BCG to enter mammalian cells. Based on these results, we propose that BCG*mertansine represents a promising avenue for future investigations and potential improvements in early‐stage bladder cancer therapy.

## 3 Conclusions

Treatment of early‐stage bladder cancer with BCG is a well‐established clinical practice. However, this approach has limitations, including nonresponsiveness in some patients, side effects such as local inflammation, urinary symptoms, and, in rare cases, systemic BCG infection.^[^ ^1^, ^2^, ^3^, ^4^, ^10^ ^]^ Additionally, the need for repetitive treatments (typically six doses) presents logistical and patient compliance challenges, highlighting the need for further improvements. Recent research has focused on genetically modifying BCG to enhance its efficacy.^[^ ^2^, ^15^ ^]^ For example, the overexpression of Ag85B has shown promising results in cellular assays, demonstrating improved killing efficiency in the 5637 bladder cancer cell line.^[^ ^11^ ^]^ Another avenue under investigation involves the formulation of BCG in liposomes, which aims to enhance its stability, targeting, and cellular uptake, potentially improving its therapeutic outcomes.^[^ ^42^ ^]^ Our novel approach functionalizes BCG with chemical entities using trehalose to specifically label the mycobacterial membrane system—an entirely new strategy for targeted modification in this context. Our analyses indicate that this approach could represent a significant advancement in BCG‐based therapies. Specifically, the use of BCG*mertansine has demonstrated promising in vitro results, and further studies are required to evaluate its activity in animal models of superficial bladder cancer. Success in these models could open a new frontier of “bug‐drug” conjugates in addition to the earlier described “bug‐nanoparticle” conjugate by Liu et al. in 2024,^[^ ^13^ ^]^ offering potential treatment options not only for bladder cancer but also for other cancer types. Future studies should compare the two approaches to identify their respective advantages and disadvantages, providing a basis for further improvement.

## 4 Experimental Section

Please see Supporting Information.

## Conflict of Interest

The authors declare no conflict of interest.

## Supporting information

Supplementary Material

## Data Availability

The data that support the findings of this study are available in the supplementary material of this article.
