# Supplementary material for: Synthesis and Evaluation of Trehalose‐Based Mertansine Warheads for Bacillus Calmette–Guérin Delivery of Anticancer Agents
Source: Chembiochem. 2025 Jul 9;26(16):e202500390. doi: 10.1002/cbic.202500390 (PMC12432487; doi:10.1002/cbic.202500390)

Supporting Information

Synthesis and Evaluation of Trehalose-Based Mertansine Warheads for BCG Delivery of Anti-Cancer Agents

Michael Grimmeisen[a]1, Xuan Wang[a,b]1, Melissa Weldle[a], Kerstin Sartory[a], Sara Benkhelifa[a], Yu Zhang[a], Trinh Dao[a][c], Jonas Meyer[a], Oliver Gorka[d], Olaf Groß[c][d], Claudia Jessen-Trefzer[a][d]*

[a] Michael Grimmeisen, Xuan Wang, Melissa Weldle, Kerstin Sartory, Sara Benkhelifa, Yu Zhang, Trinh Dao, Jonas Meyer, Claudia Jessen-Trefzer

University of Freiburg, Institute of Organic Chemistry, Albertstrasse 21, 79104 Freiburg, Germany

[b] Xuan Wang

Zhongshan Institute for Drug Discovery, Shanghai Institute of Materia Medica, Chinese Academy of Sciences, Zhongshan 528400, China

[c] Trinh Dao, Olaf Groß, Claudia Jessen-Trefzer

CIBSS - Centre for Integrative Biological Signalling Studies, University of Freiburg, Schänzlestrasse 18, 79104 Freiburg, Germany

[d] Oliver Gorka, Olaf Groß

University of Freiburg, Institute of Neuropathology University Medical Center, Faculty of Medicine, Breisacher Straße 113, 79106 Freiburg, Germany

[1]equal contribution

E-mail: claudia.jessen-trefzer@pharmazie.uni-freiburg.de

Contents

Supporting Figures 3

Material and methods 10

Chemistry 10

Chemicals, analytics and general remarks 10

Liquid chromatography 10

HPLC analyses ... 10
Analytics ... 10
Synthesis of 6-amino-trehalose (6) ... 11
6-p-Toluenesulfonyl trehalose: ... 11
6-Azido-trehalose: ... 11
6-Amino-trehalose (6): ... 12
Synthesis of trehalose-fluorescein conjugates (C_2 (10), PEG_4 (11) and PEG_{12} (12)) ... 12
2-(tritylthio)ethan-1-amine (3): ... 12
5-(2-Tritylthio-ethyl)carbamoyl fluorescein (4): ... 13
5-(2-mercaptoethyl)-carbamoyl fluorescein (5): ... 13
Trehalose-C_2 linker (7): ... 14
Trehalose-C_2 fluorescein (10): ... 14
Trehalose-PEG_4 linker (8): ... 15
Trehalose-PEG_4 fluorescein (11): ... 15
Trehalose-PEG_{12} linker (9): ... 16
Trehalose-PEG_{12} fluorescein (12): ... 16
Synthesis of trehalose-PEG_4 mertansine (2) ... 16
Synthesis of trehalose-BODIPY (13) and trehalose-I-BODIPY (1) ... 17
Trehalose-BODIPY (13): ... 17
Trehalose-I-BODIPY (1): ... 18
Biology ... 18
Bacterial strains and growth conditions ... 18
Mammalian cells and growth conditions ... 18
MIC assay against *M. bovis* BCG ... 19
Mertansine release from trehalose-PEG_4-mertansine (2) with dithiothreitol (DTT) ... 20
Medium stability assay of trehalose-PEG_4-mertansine (2) ... 20
IC_{50} assay against HT-1376 and T24 cell lines ... 20
Lipid extraction of labeled *M. bovis* BCG ... 21

Lipid extraction of *M. smegmatis* for linker length screening 21

Lipid analysis by TLC assay 22

Purification of Ag85 enzymes 22

Ag85 *in vitro* processing assay 23

Cell inactivation assay with labeled bacteria 23

IL-6 and TNF-α ELISA assay of BCG infected BMDMs 24

Fluorescence microscopy of internalized bacteria 25

Literature 25

Appendix: Analytical Data (^{1}H-NMR, ^{13}C-NMR, ^{11}B, ^{19}F, 2D-NMR, HR-MS and HPLC Chromatograms) 26

Supporting Figures

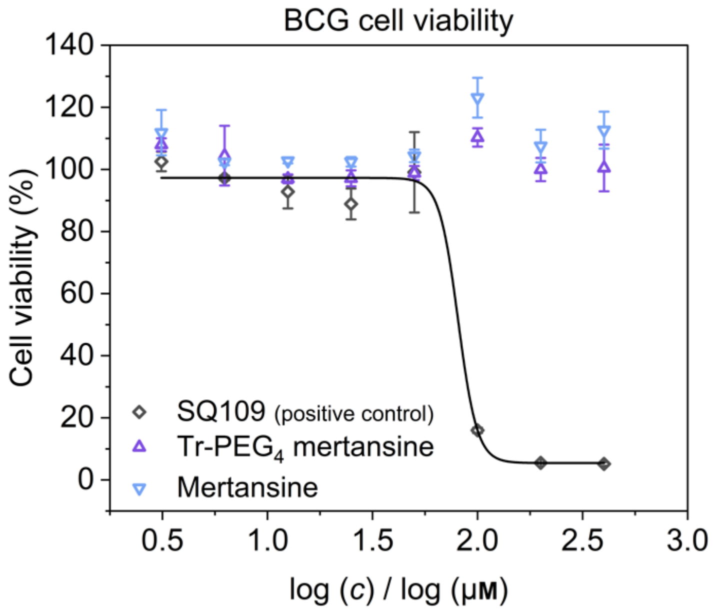

Figure S1: Viability screening of trehalose-PEG$_4$ mertansine (**2,** Tr-PEG$_4$ mertansine) and mertansine against BCG. Incubation time: 24 hours. SQ109 was used as positive control.

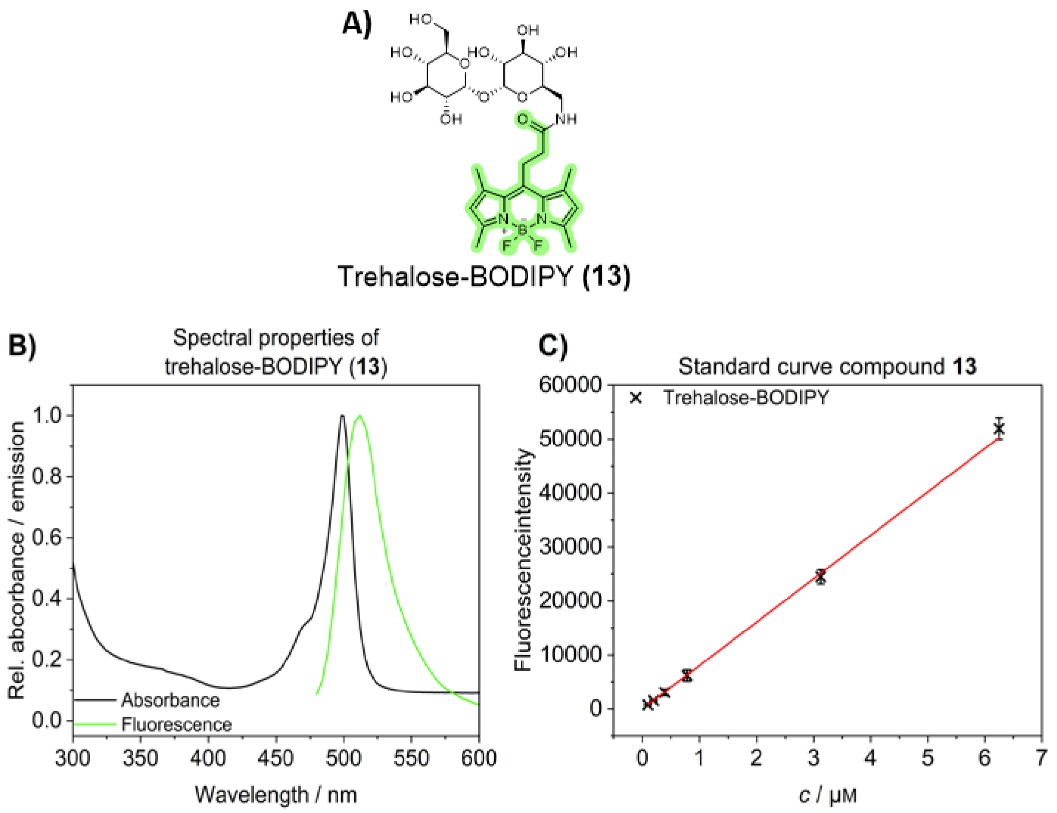

Figure S2: Characterization of trehalose-BODIPY (**13**). A) Molecular structure of **13**. B) Spectral properties of **13**. C) Standard curve generated for determining concentrations of **13** in lipid extracts. Excitation (Ex) = 498 nm; Em = 512 nm.

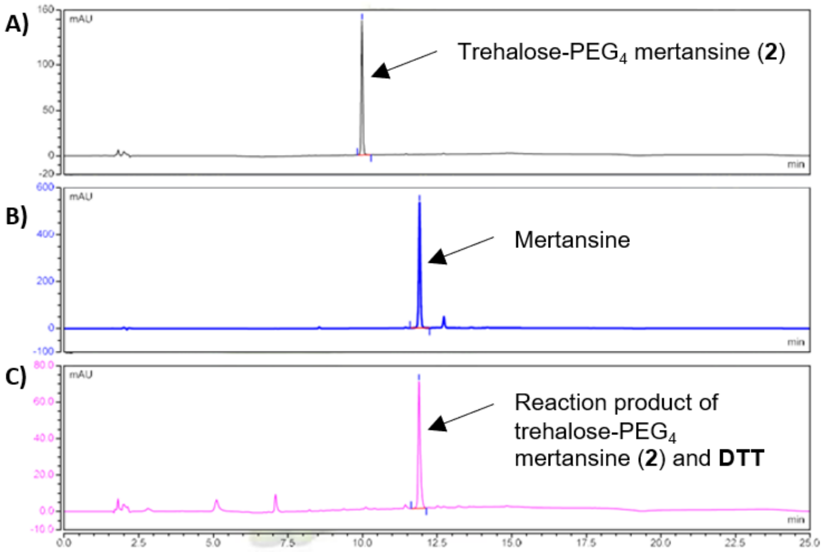

Figure S3: HPLC traces of the drug release study using DTT. A) Trehalose-PEG$_4$ mertansine (**2**) in PBS. B) Mertansine only. C) Trehalose-PEG$_4$ mertansine (**2**) treated with 10 mM DTT for 30 min at 37 °C in PBS.

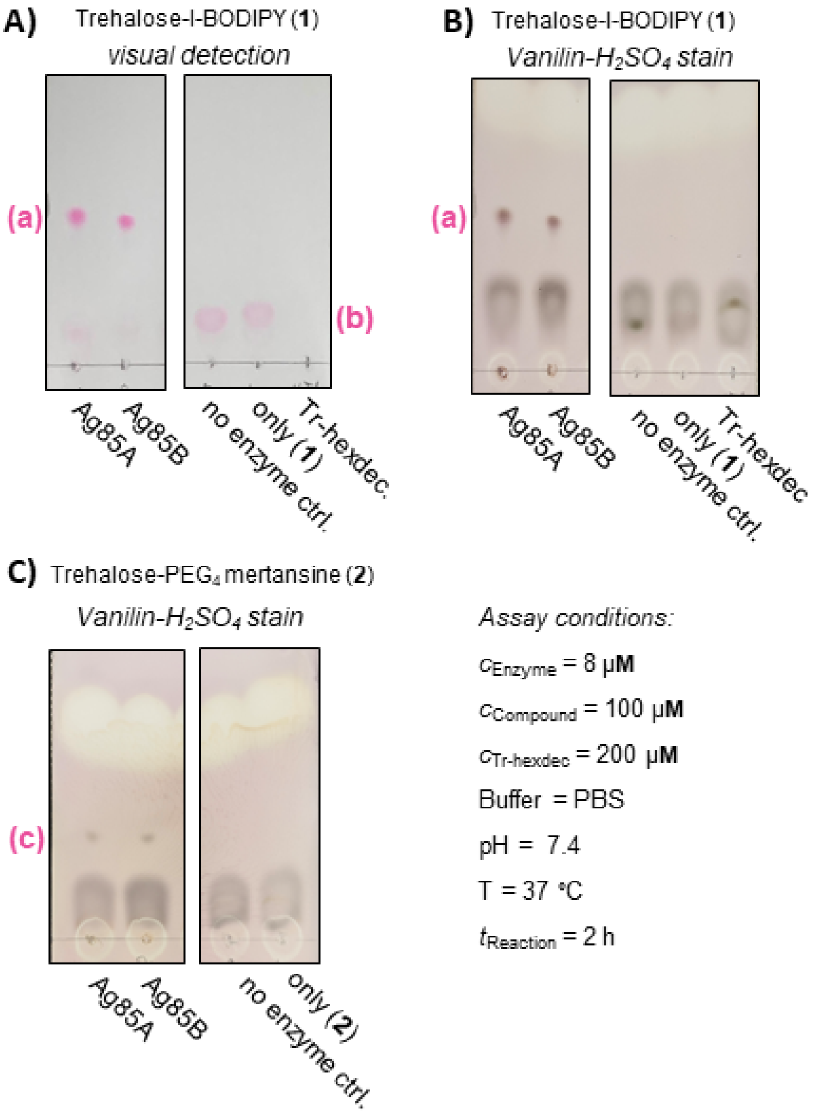

(a) Trehalose-I-BODIPY-hexadecanoate; identified via HR-MS: 1178.2936 Da $(M+COO)^-$
(b) Trehalose-I-BODIPY
(c) Trehalose-PEG_4 mertansine-adduct

Figure S4: Ag85 incorporation assay using trehalose-PEG_4 mertansine (**2**) and trehalose-I-BODIPY (**1**) as substrates and trehalose-hexadecanoate as lipid-donor. A) Unstained TLC plate of the reaction using trehalose-I-BODIPY (**1**) as substrate, to see the difference in retention factors (Rf) between trehalose-I-BODIPY (**1**) and the hexadecanoate modified product. The adduct was identified by HR-MS. Spot (a) showed a mass of 1178.1940 Da in ESI (− mode) which matches the mass of trehalose-I-BODIPY-hexadecanoate. HR-MS calculated ($M + COO^-$): 1178.2936 Da. B) Vanilin-H_2SO_4 stained TLC plate of the reaction using trehalose-I-BODIPY (**1**) as substrate. C) Vanilin-H_2SO_4 stained TLC plate of the reaction using trehalose-PEG_4 mertansine (**2**) as substrate. HR-MS analysis of spot (c), potentially trehalose-PEG_4-mertansine-hexadecanoate was not successful, probably due to the low concentration of the compound in this type of assay. However, spot (c) and (a) show comparable Rf values indicating that (c) is the product trehalose-PEG_4-mertansine-hexadecanoate. No enzyme means whole reaction mixture without enzyme, Tr-hexdec means trehalose-hexadecanoate.

A)

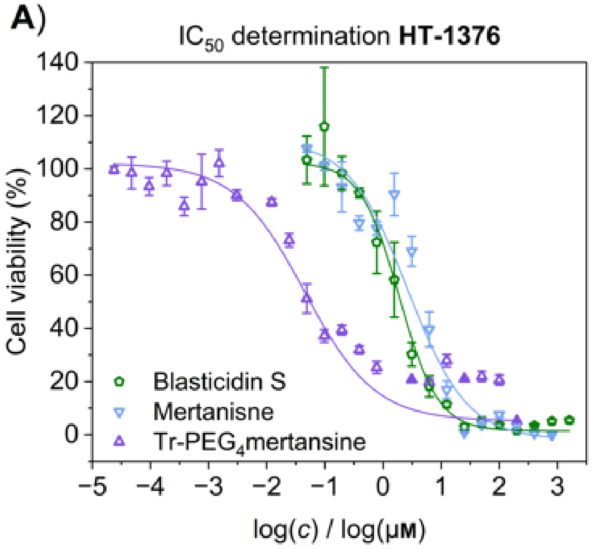

B)

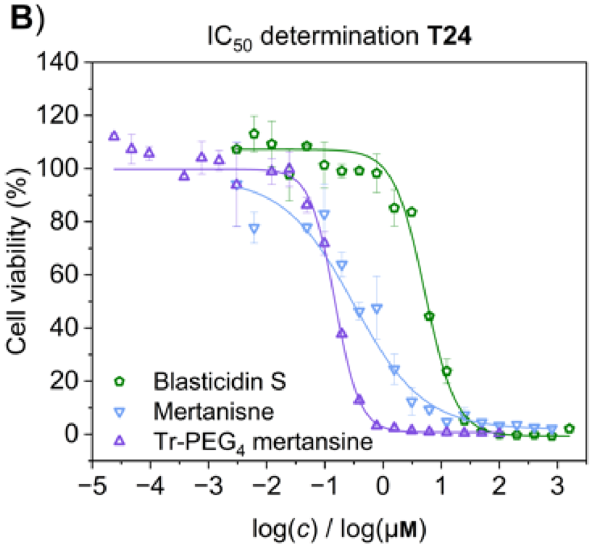

Figure S5: IC_{50} screening of mertansine and trehalose-PEG_4 mertansine (**2,** Tr-PEG_4 mertansine) against bladder cancer cells (Blasticidin S, positive control). A) HT-1376 cell line. B) T24 cell line. Experiments were performed at least in triplicates.

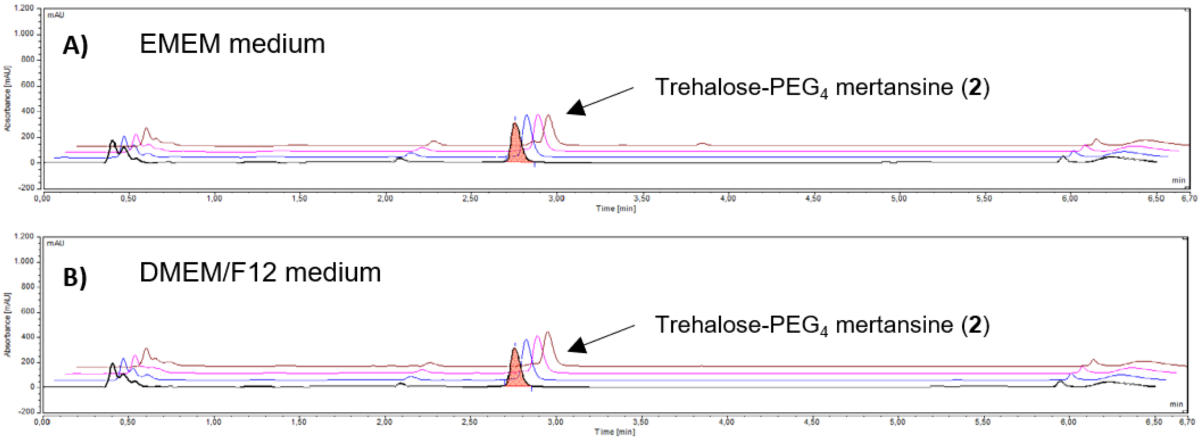

Figure S6: HPLC traces at 258 nm showing the stability of trehalose-PEG$_4$-mertansine (**2**) in cell culture medium. A) In Minimum Essential Medium Eagle (EMEM) at time points 0 h (black), 1 h (blue), 6 h (pink) and 24 h (brown). B) In Dulbecco's Modified Eagle's Medium F12 (DMEM/F12) at time points 0 min (black), 1 h (blue), 6 h (pink) and 24 h (brown).

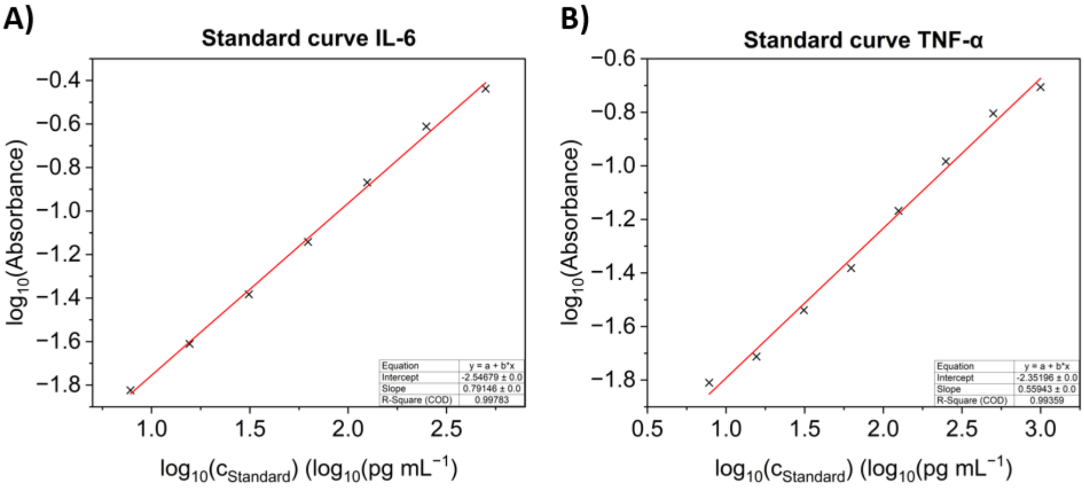

Figure S7: Double logarithmic standard curves for the ELISA concentration determination. A) IL-6 and B) TNF-α.

Material and methods

Chemistry

Chemicals, analytics and general remarks

Reactions were carried out in an open flask equipped with a magnetic stirrer at room temperature, unless otherwise noted.

Reagents were purchased from commercial suppliers (Acros, Sigma Aldrich, Fisher Scientific, Fluka, TCI, BLD Pharma, Carl Roth) and used as received, unless noted otherwise.

Solvents were obtained in analytical grade and used as received.

HPLC solvents were used as obtained for preparative HPLC and MPLC purifications.

Deuterated solvents for NMR were obtained from Euriso-Top (Germany) and Deutero (Germany) in the indicated purity grade and used as received for NMR spectroscopy.

Liquid chromatography

Liquid chromatography was performed using 1) a preparative RP-HPLC equipped with an aQ.C18 column coupled to a LT-ELSD and 2) an automated RP-MPLC, equipped with an aQ.C18 column and coupled to a LT-ELSD.

HPLC analyses

HPLC analyses were performed using a Thermo Scientific (Dionex, Ultimate 3000) analytical HPLC equipped with 1) a Hypersil GOLD aQ.C18 column (3 µ, 3 × 150 mm) coupled to a UV and ESI-MS detector, 2) a Hypersil GOLD C4 column (3 µ, 3 × 150 mm) coupled to a UV and ESI-MS detector and 3) Waters XSelect Premier CSH C18 VanGuard FIT Column, 130 Å, 2.5 µm, 2.1 x 50 mm coupled to a UV/Vis detector.

Analytics

^{1}H-NMR spectra were recorded on Bruker 300 MHz spectrometers, Bruker 400 MHz and Bruker 500 MHz spectrometers in the indicated deuterated solvent. Data are reported as follows: chemical shift (δ, ppm), multiplicity (s, singlet; d, doublet; t, triplet; q, quartet; m, multiplet; br, broad signal), coupling constant(s) (J, Hz), integration. All signals were referenced to the internal solvent signal as standard (CD_3OD, δ 3.31; $(CD_3)_2SO$, δ 2.50; $CDCl_3$, δ 7.26).

^{13}C-NMR spectra were recorded with ^{1}H-decoupling on Bruker 101 MHz (with cryoprobe) spectrometers at 298K in the indicated deuterated solvent. All signals were referenced to the internal solvent signal as standard (CD_3OD, δ 49.0; $(CD_3)_2SO$, δ 39.52; $CDCl_3$, δ 77.5).

Mass spectra were recorded at the mass spectrometry service at the University of Freiburg on Finnigan TSQ 700 MS and Thermo Scientific EXACTIVE spectrometers with Orbitrap analyzer.

Synthesis of 6-amino-trehalose (6)

6-p-Toluenesulfonyl trehalose:

The 6-amino trehalose **(6)** building block was synthesized according to published literature.[1–3] Trehalose (856 mg, 2.5 mmol, 1.0 eq) was dissolved in anhydrous pyridine (80 mL). The mixture was cooled to −35 °C, and a solution of p-toluenesulfonyl chloride (477 mg, 2.5 mmol, 1.0 eq) in anhydrous pyridine (20 mL) was added. The reaction mixture was stirred at −35 °C for 9 h. Subsequently the solvent was removed under reduced pressure, and the obtained residue was isolated using an Interchim Puriflash, a reverse phase column (PuriFlash Column 0120, C18 AQ 30 µm) and a gradient of acetonitrile / UP water from 5% to 40%. 6-p-toluenesulfonyl trehalose was obtained as white solid (372 mg, 0.75 mmol, 30%, lit.[1] 27%). 6-p-toluenesulfonyl trehalose: ^{1}H-NMR (400 MHz, Methanol-d_4) δ = 7.86 – 7.77 (m, 2H), 7.45 (d, *J* = 8.2 Hz, 2H), 5.02 (d, *J* = 3.7 Hz, 1H), 4.99 (d, *J* = 3.7 Hz, 1H), 4.28 – 4.18 (m, 2H), 4.00 (ddd, *J* = 10.1, 4.7, 2.2 Hz, 1H), 3.85 – 3.66 (m, 5H), 3.47 (dd, *J* = 9.8, 3.7 Hz, 1H), 3.41 (dd, *J* = 9.8, 3.7 Hz, 1H), 3.32 – 3.23 (m, 1H), 2.47 (s, 3H). ^{13}C-NMR (101 MHz, $CDCl_3$) δ = 146.9, 134.8, 131.5, 129.6, 95.7, 95.6, 75.0, 74.8, 74.3, 73.6, 73.4, 72.3, 71.8, 71.7, 71.2, 63.1, 22.1. HR-MS (ESI−): m/z calc. for $[M + {}^{35}Cl]^-$: 531.0940, found: 531.0937.

6-Azido-trehalose:

6-Azido-trehalose was synthesized adapted to a procedure described by V. Cucinotta *et al.* (2007).[2] 6-p-toluenesulfonyl trehalose (250 mg, 0.5 mmol, 1.0 eq) was dissolved in dimethylformamide (16 ml) and mixed with an aqueous solution (4 ml) of NaN_3 (100 mg, 1.5 mmol, 3 eq). The reaction mixture was stirred for 20 h at 95 °C. Afterwards the solvent was removed under reduced pressure. The crude product obtained was isolated using an Interchim Puriflash, a reverse phase column and a gradient of methanol / UP water from 0% to 20%. 6-azido trehalose was obtained as white solid (156 mg, 0.425 mmol, 85%, lit.[2] 87%).

^{1}H-NMR (300 MHz, D_2O) δ = 5.22 (t, *J* = 3.5 Hz, 2H), 3.99 (dddd, *J* = 9.8, 5.8, 2.5, 0.0 Hz, 1H), 3.92 – 3.64 (m, 8H), 3.58 (dd, *J* = 13.6, 5.8 Hz, 1H), 3.53 – 3.43 (m, 2H). ^{13}C-NMR (101 MHz, D_2O) δ = 93.6, 93.4, 72.5, 72.3, 72.2, 71.0, 70.9, 70.9, 70.4, 69.6, 60.5, 50.8.

HR-MS: calc. ($C_{12}H_{21}N_3O_{10} - H^+$): 366.1154, found (ESI−): 366.1155.

6-Amino-trehalose (6):

Subsequently, 6-azido trehalose was converted to 6-amino trehalose (**6**) by catalytic hydrogenation on Pd/C with H_2 according to a procedure adapted from F.-P. Rodriguez-Rivera *et al.* (2017).[3] 6-Azido trehalose (1.387 g, 3.776 mmol, 1.0 eq) was dissolved in methanol (189 ml) and 2 mol% Pd/C (10%, 80.37 mg) was added. The reaction mixture was flushed with argon for a few minutes to remove dissolved oxygen before the reaction was started by applying a hydrogen atmosphere. The reaction was stirred over night at room temperature and terminated by flushing with argon. Finally, the heterogeneous catalyst was removed by filtration through Celite®. 6-Amino trehalose **(6)** was obtained as a white crystalline solid (1.279 g, 3.75 mmol, 99.2%).

^{1}H-NMR (500.30 MHz, presat, D_2O): δ = 2.67-2.74 (m, 1H, 12-H or 12’-H), 2.94- 2.99 (m, 1H, 12-H or 12’-H), 3.25-3.30 (m, 1H), 3.36-3.41 (m, 1H), 3.57-3.61 (m, 2H), 3.68-3.81 (m, 6H), 5.13 (d, 1H, *J*1,2 or 7,8 = 4.34 Hz, 1-H or 7-H), 5.14 (d, 1H, *J*1,2 or 7,8 = 4.14 Hz, 1-H or 7-H). ^{13}C-NMR (125.80 MHz, D_2O) δ = 41.4 (1C, C-12), 60.5 (1C, C-6), 69.7 (1C), 71.0 (1C), 71.1 (1C), 71.4 (1C), 72.1 (1C), 72.2 (1C), 72.4 (1C), 72.5 (1C), 93.1 (1C, C-7), 93.2 (1C, C-1). HR-MS: calc. ($C_{12}H_{23}NO_{10} - H^+$): 340.1249, found (ESI−): 340.1248.

Synthesis of trehalose-fluorescein conjugates (C_2 (10), PEG_4 (11) and PEG_{12} (12))

2-(tritylthio)ethan-1-amine (3):

The synthesis procedure was adapted and modified from M. Togashi *et al.* (2014).[4] Cysteamine (772 mg, 10 mmol, 1.0 eq) was added to a solution of dry dichloromethane (5 mL) containing trityl chloride (2.79 g, 10 mmol, 1.0 eq). Subsequently trifluoroacetic acid (2 mL) was added dropwise to the mixture. After incubation for 2 h at rt. under argon atmosphere, a 1 M aqueous sodium hydroxide solution (3 mL) was added to the mixture. The product was extracted with ethyl acetate, and the organic solution was washed with brine, dried with anhydrous Na_2SO_4. The solvent was removed under reduced pressure. The crude product was purified by column chromatography on silica gel (ethyl acetate/methanol = 9/1) to afford 2-(tritylthio)ethan-1-amine **(3)** as a white solid with a yield of 60%.

^{1}H-NMR (300 MHz, $CDCl_3$) δ = 7.48 – 7.36 (m, 6H), 7.35 – 7.17 (m, 9H), 2.57 (t, *J* = 6.5 Hz, 2H), 2.36 (t, *J* = 6.5 Hz, 2H). ^{13}C-NMR (101 MHz, $CDCl_3$) δ = 144.8, 129.6, 128.0, 126.7, 66.7, 41.0, 35.2. Analytical data were identical to literature.

5-(2-Tritylthio-ethyl)carbamoyl fluorescein (4):

Next a mixture of 2-(tritylthio)ethan-1-amine **(3)** (143.6 mg, 0.45 mmol, 1.5 eq) in dimethylformamide (10 mL) was added dropwise to a mixture of 5/6-carboxyfluorescein succinimidyl ester (142 mg, 0.3 mmol, 1.0 eq) in dimethylformamide (10 mL) at rt. After 2 h, the solvent was removed under reduced pressure and the residue was purified by flash chromatography on silica gel (10:1 dichloromethane/methanol v/v). The mixed isomers were obtained as yellow oil with a yield of 70%. The obtained isomers were separated and isolated using a Interchim Puriflash, on a reverse-phase column (C_{18} AQ) with elution of methanol/UP water from 40% to 65%. 5-(2-tritylthio-ethyl)carbamoyl fluorescein **(4)** was obtained with a yield of 35% while 6-(2-tritylthio-ethyl)carbamoyl fluorescein was obtained with a yield of 20%.

5-(2-tritylthio-ethyl)carbamoyl fluorescein **(4)**: ^{1}H-NMR (400 MHz, Methanol-d_4) δ = 8.36 (s, 1H), 8.08 (dd, J = 8.1, 1.4 Hz, 1H), 7.33 (d, J = 7.5 Hz, 6H), 7.22 – 7.07 (m, 11H), 6.66 (d, J = 2.3 Hz, 2H), 6.53 (d, J = 8.7 Hz, 2H), 6.47 (dd, J = 8.7, 2.3 Hz, 2H), 3.31 – 3.27 (m, 2H), 2.45 (t, J = 6.8 Hz, 2H). ^{13}C-NMR (101 MHz, Methanol-d_4) δ = 169.4, 166.9, 166.8, 160.6, 152.8, 144.8, 136.2, 136.1, 134.0, 129.3, 129.2, 129.0, 128.9, 127.8, 127.6, 127.5, 126.8, 126.5, 126.4, 124.5, 123.7, 112.7, 109.6, 102.3, 66.4, 38.8, 31.3. HR-MS (ESI+): m/z calc. for $[M + H]^+$: 678.1945, found: 678.1946.

6-(2-tritylthio-ethyl)carbamoyl fluorescein: ^{1}H-NMR (400 MHz, Methanol-d_4) δ 7.98 (s, 2H), 7.49 (s, 1H), 7.22 (dd, J = 8.2, 1.4 Hz, 6H), 7.14 – 7.02 (m, 9H), 6.64 – 6.52 (m, 4H), 6.45 (dd, J = 8.8, 2.3 Hz, 2H), 3.16 (t, J = 6.6 Hz, 2H), 2.27 (t, J = 6.6 Hz, 2H). ^{13}C-NMR (101 MHz, Methanol-d_4) δ 166.7, 144.7, 129.2, 127.4, 126.4, 102.3, 66.2, 33.4, 25.3. HR-MS (ESI+): m/z calc. for $[M + H]^+$: 678.1945, found: 678.1942.

5-(2-mercaptoethyl)-carbamoyl fluorescein (5):

Triethylsilane (1 mL) in dichloromethane (10 mL) was added dropwise at 0 °C to a mixture of 5-(2-tritylthio-ethyl)carbamoyl fluorescein **(4)** (101.7 mg, 0.15 mmol) in trifluoroacetic acid (10 mL). The reaction mixture was degassed by purging with argon for 5 min, then stirred at rt for 12 h. The solvent was removed under reduced pressure and the crude product was purified by flash chromatography using silica gel (step gradient from 10:1 to 5:1 dichloromethane/methanol v/v). 5-(2-mercaptoethyl)-carbamoyl fluorescein **(5)** was obtained as a yellow amorphous solid with a yield of 90%

^{1}H-NMR (500 MHz, Methanol-d_4) δ = 8.47 (s, 1H), 8.22 (dd, J = 8.0, 1.5 Hz, 1H), 7.33 (d, J = 8.0 Hz, 1H), 6.71 (d, J = 2.3 Hz, 2H), 6.63 (d, J = 8.7 Hz, 2H), 6.56 (dd, J = 8.7, 2.4 Hz, 2H),

3.62 (t, *J* = 7.0 Hz, 2H), 2.79 (t, *J* = 6.9 Hz, 2H). ^{13}C-NMR (126 MHz, Methanol-d_4) δ = 170.7, 168.5, 154.2, 137.7, 135.3, 130.2, 125.9, 125.0, 113.9, 111.0, 103.6, 44.6, 24.3. HR-MS (ESI+): m/z calc. for $[M + H]^+$, 436.0849; found: 436.0847.

Trehalose-C_2 linker (7):

At rt, 6-amino-trehalose **(6)** (25 mg, 72 µmol, 1.2 eq) in dimethylformamide (2 mL) was added to a solution of 3-(2-Pyridyldithio)propionic acid NHS ester (18.7 mg, 0.06 mmol, 1.0 eq) in dimethylformamide (2 mL). The reaction was stirred for 2 h, and the solvent was removed under reduced pressure. The residue was purified by flash chromatography on silica gel (10:1 to 1:1 dichloromethane/methanol v/v). The trehalose-C_2 linker **(7)** was obtained as colorless oil with a yield of 90%.

^{1}H-NMR (400 MHz, Methanol-d_4) δ = 8.50 – 8.33 (m, 1H), 7.93 – 7.73 (m, 2H), 7.25 (ddd, *J* = 6.7, 4.9, 1.8 Hz, 1H), 5.10 (t, *J* = 4.0 Hz, 2H), 3.99 – 3.74 (m, 6H), 3.69 (dd, *J* = 12.0, 5.5 Hz, 1H), 3.57 – 3.43 (m, 4H), 3.25 – 3.15 (m, 1H), 3.08 (t, *J* = 7.0 Hz, 2H), 2.72 – 2.63 (m, 2H). ^{13}C-NMR (101 MHz, Methanol-d$_4$) δ = 174.0, 161.1, 150.4, 139.2, 122.4, 121.2, 95.3, 95.2, 74.6, 74.1, 73.9, 73.2, 73.1, 72.0, 71.9, 62.6, 41.4, 36.0, 35.5. HR-MS (ESI+): m/z calc. for $[M + H]^+$: 539.1364, found: 539.1370.

Trehalose-C_2 fluorescein (10):

5-(2-Mercaptoethyl)-carbamoyl fluorescein **(5)** (2.5 mg, 5.7 µmol, 1.70 eq) in methanol (1 mL) was added dropwise to a solution of trehalose-C_2 linker **(7)** (2.6 mg, 3.3 µmol, 1.0 eq) in methanol (0.5 mL) at rt, the mixture was stirred for 15 h. The solvent was removed under reduced pressure and the residue was purified by flash chromatography on silica gel (step gradient from 5:1 dichloromethane/methanol v/v to pure methanol). Trehalose-C_2 fluorescein **(10)** was obtained as an orange amorphous solid with a yield of 75% and a purity of >95%

^{1}H-NMR (400 MHz, Methanol-d_4) δ = 8.49 (d, *J* = 1.3 Hz, 1H), 8.15 – 8.08 (m, 1H), 7.34 (d, *J* = 8.0 Hz, 1H), 6.93 (d, *J* = 9.0 Hz, 2H), 6.69 (d, *J* = 2.2 Hz, 2H), 6.62 (dd, *J* = 9.0, 2.2 Hz, 2H), 5.09 (d, *J* = 3.6 Hz, 2H), 3.93 – 3.72 (m, 7H), 3.71 – 3.62 (m, 1H), 3.56 – 3.44 (m, 4H), 3.21 (t, *J* = 9.4 Hz, 1H), 3.03 (td, *J* = 5.9, 4.7, 2.6 Hz, 4H), 2.69 (t, *J* = 7.0 Hz, 2H). ^{13}C-NMR (101 MHz, Methanol-d_4) δ = 173.0, 167.7, 155.9, 135.6, 130.3, 127.7, 126.4, 117.7, 112.4, 102.5, 93.9, 93.9, 83.1, 73.2, 72.7, 72.5, 71.9, 71.7, 70.7, 70.5, 61.2, 40.0, 39.2, 37.0, 35.1, 34.1. HR-MS (ESI+): m/z calc. for $[M + Na]^+$: 885.1817, found: 885.1804.

Trehalose-PEG_4 linker (8):

The synthesis of trehalose-PEG_4 linker (**8)** was adapted from R. S. Navath *et al.* (2008).[5] 6-amino trehalose **(6)** (68 mg, 0.20 mmol, 1.1 eq) in dimethylformamide (10 mL) was added dropwise to a solution of SPDP-dPEG®$_4$-NHS ester (100 mg, 0.178 mmol, 1.0 eq) in dimethylformamide (5 mL) at rt. The reaction mixture was stirred overnight at rt until the solvent was removed under reduced pressure. The residue was purified by means of flash chromatography on silica gel (1:1 dichloromethane/methanol v/v) and the trehalose-PEG_4 linker **(8)** was obtained as a colorless oil with a yield of 90%.

^{1}H-NMR (400 MHz, Methanol-d_4) δ = 8.44 – 8.37 (m, 1H), 7.88 – 7.76 (m, 2H), 7.22 (ddd, *J* = 6.7, 4.9, 1.6 Hz, 1H), 5.08 (t, *J* = 3.3 Hz, 2H), 3.88 (dt, *J* = 9.4, 4.4 Hz, 1H), 3.84 – 3.72 (m, 6H), 3.71 – 3.64 (m, 2H), 3.64 – 3.56 (m, 12H), 3.54 (t, *J* = 5.5 Hz, 2H), 3.51 – 3.42 (m, 4H), 3.36 (t, *J* = 5.4 Hz, 2H), 3.20 – 3.11 (m, 1H), 3.06 (t, *J* = 7.0 Hz, 2H), 2.63 (t, *J* = 7.0 Hz, 2H), 2.47 (td, *J* = 6.0, 2.0 Hz, 2H). ^{13}C-NMR (101 MHz, Methanol-d_4) δ = 177.2, 175.9, 163.8, 153.0, 152.9, 141.7, 124.9, 123.7, 97.9, 97.8, 77.2, 76.7, 76.4, 75.9, 75.8, 75.6, 74.5, 74.5, 74.1, 74.1, 74.0, 74.0, 73.9, 73.8, 73.1, 70.8, 65.2, 43.9, 43.1, 40.0, 38.6, 38.0. HR-MS (ESI+): m/z calc. for $[M + H]^+$: 786.2784, found: 786.2775.

Trehalose-PEG_4 fluorescein (11):

5-(2-Mercaptoethyl)-carbamoyl fluorescein **(5)** (2.5 mg, 5.7 µmol, 1.7 eq) in methanol (1 mL) was added dropwise to a stirred solution of trehalose-PEG_4 linker **(8)** (2.6 mg, 3.3 µmol, 1.0 eq) in methanol (0.5 mL) at rt. After 15 h the solvent was removed under reduced pressure and the residue was purified using flash chromatography on silica gel (step gradient from 5:1 to 2:1 dichloromethane/methanol v/v, 2:1 dichloromethane/methanol v/v, then 1:1 dichloromethane/methanol v/v to eluate the product). The trehalose-PEG_4 fluorescein **(11)** was obtained as an orange amorphous solid with a yield of 75% and a purity of >98%

^{1}H-NMR (400 MHz, Methanol-d_4) δ = 8.36 (s, 1H), 7.90 (dt, *J* = 7.9, 2.0 Hz, 1H), 7.23 (d, *J* = 7.9 Hz, 1H), 6.92 (dd, *J* = 9.1, 2.9 Hz, 2H), 6.51 – 6.35 (m, 4H), 4.98 (q, *J* = 5.2, 4.8 Hz, 2H), 3.79 (dd, *J* = 6.5, 3.3 Hz, 1H), 3.75 – 3.43 (m, 24H), 3.38 (ddq, *J* = 11.1, 8.0, 3.7 Hz, 4H), 3.34 – 3.27 (m, 2H), 3.06 (td, *J* = 9.5, 2.9 Hz, 1H), 2.95 (dt, *J* = 16.8, 7.0 Hz, 3H), 2.85 (t, *J* = 7.1 Hz, 1H), 2.54 (dt, *J* = 21.1, 7.1 Hz, 2H), 2.44 – 2.35 (m, 2H). ^{13}C-NMR (101 MHz, Methanol-d_4) δ = 173.3, 172.5, 171.9, 171.8, 168.2, 168.2, 140.8, 136.4, 134.9, 134.9, 130.8, 129.9, 127.9, 127.4, 122.7, 111.6, 111.6, 103.0, 93.9, 93.9, 73.2, 72.7, 72.5, 71.9, 71.8, 71.7, 70.5, 70.0, 69.8, 69.8, 67.0, 61.2, 39.9, 39.1, 39.0, 37.2, 35.2, 35.1, 33.9, 33.7. HR-MS (ESI+): m/z calc. for $[M + Na]^+$: 1132.3237, found: 1132.3260.

Trehalose-PEG$_{12}$ linker (9):

This synthesis of the trehalose-PEG$_{12}$ linker **(9)** was adapted from R. S. Navath *et al.* (2008).[5] 6-Amino trehalose **(6)** (37.5 mg, 0.11 mmol, 1.1 eq) in dimethylformamide (10 mL) was added dropwise to a solution of SPDP-dPEG®$_{12}$-NHS ester (91.2 mg, 0.10 mmol, 1.0 eq) in dimethylformamide (5 mL) at rt. The reaction mixture was stirred overnight at rt until the solvent was removed under reduced pressure. The residue was purified by means of flash chromatography on silica gel (2:1 dichloromethane/methanol v/v) and the trehalose-PEG$_{12}$ linker **(9)** was obtained as a colorless oil with a yield of 84%.

^{1}H-NMR (300 MHz, Methanol-*d*$_4$) δ = 8.50 – 8.41 (m, 1H), 7.87 (dd, *J* = 6.4, 1.6 Hz, 2H), 7.27 (ddd, *J* = 6.7, 4.9, 2.0 Hz, 1H), 5.13 (dd, *J* = 3.6, 2.3 Hz, 2H), 4.00 – 3.60 (m, 53H), 3.59 (t, *J* = 5.4 Hz, 2H), 3.58 – 3.48 (m, 4H), 3.41 (t, *J* = 5.2 Hz, 2H), 3.27 – 3.14 (m, 1H), 3.11 (t, *J* = 7.0 Hz, 2H), 2.68 (t, *J* = 7.0 Hz, 2H), 2.60 – 2.45 (m, 2H). ^{13}C-NMR (101 MHz, Methanol-*d*$_4$) δ = 173.3, 172.0, 159.8, 149.1, 137.8, 121.0, 119.7, 93.9, 93.9, 73.2, 72.7, 72.5, 71.9, 71.8, 71.6, 70.6, 70.5, 70.2, 70.1, 70.1, 70.0, 69.9, 69.9, 69.1, 66.9, 61.2, 39.9, 39.1, 36.1, 34.7, 34.1. HR-MS (ESI+): m/z calc. for $[M + H]^+$: 1138.4881, found: 1138.4881.

Trehalose-PEG$_{12}$ fluorescein (12):

5-(2-mercaptoethyl)-carbamoyl fluorescein **(5)** (2.5 mg, 5.7 µmol, 1.6 eq) dissolved in methanol (1 mL) was added to a solution of trehalose-PEG$_{12}$ linker **(9)** (4.0 mg, 3.5 µmol, 1.0 eq) in methanol (0.5 mL) at rt. The solvent was removed under reduced pressure after 20 h of stirring. The residue was purified by flash chromatography on silica gel (step gradient from 5:1 dichloromethane/methanol v/v to pure methanol). Trehalose-PEG$_{12}$ fluorescein **(12)** was obtained as an orange amorphous solid with a yield of 70% and a purity of >94%.

^{1}H-NMR (400 MHz, Methanol-*d*$_4$) δ = 8.35 (s, 1H), 7.94 – 7.88 (m, 1H), 7.23 (d, *J* = 7.9 Hz, 1H), 6.92 (dd, *J* = 9.1, 3.3 Hz, 2H), 6.42 (d, *J* = 12.0 Hz, 4H), 5.03 – 4.94 (m, 2H), 3.89 – 3.41 (m, 56H), 3.38 (s, 4H), 3.29 (t, *J* = 5.7 Hz, 3H), 3.06 (t, *J* = 9.4 Hz, 1H), 3.01 – 2.89 (m, 3H), 2.85 (t, *J* = 7.1 Hz, 1H), 2.54 (dt, *J* = 22.5, 7.1 Hz, 2H), 2.40 (q, *J* = 7.7, 5.9 Hz, 2H). ^{13}C-NMR (101 MHz, Methanol-*d*$_4$) δ = 173.2, 172.4, 172.4, 171.7, 168.1, 158.3, 140.9, 140.9, 136.4, 134.8, 130.8, 129.9, 127.9, 127.4, 122.7, 111.5, 111.5, 103.1, 93.9, 93.9, 73.2, 72.7, 72.5, 71.9, 71.8, 71.7, 70.6, 70.5, 70.0, 69.9, 69.8, 69.7, 69.6, 69.3, 66.9, 61.2, 39.9, 39.1, 39.0, 37.2, 35.9, 35.2, 35.0, 34.0, 33.7. HR-MS (ESI+): m/z calc. for $[M + Na]^+$: 1484.5334, found: 1484.5353.

Synthesis of trehalose-PEG$_4$ mertansine (2)

Acetic acid (1 mL) in dimethylformamide (10 mL) was purged with argon for 10 min. Mertansine (81.2 mg, 0.11 mmol, 1.1 eq) dissolved in the previously degassed acetic

acid/dimethylformamide mixture (1:10 v/v, 6 mL) was added dropwise to a solution of trehalose-PEG_4 linker (**8)** (78.6 mg, 0.1 mmol, 1.0 eq) in the previously degassed acetic acid/dimethylformamide mixture (1:10 v/v, 1 mL) at 0 °C. Afterwards the reaction mixture was stirred for 12 h at rt. The solvent was removed under reduced pressure and the corresponding residue was purified by means of flash chromatography on silica gel (2:1 dichloromethane/methanol v/v). The resulting compound was repurified using a Interchim Puriflash, on a C_{18} column with elution of acetonitrile/UP water from 5% to 40%. Trehalose-PEG_4 mertansine (**2**) was obtained as a colorless HPLC pure oil with a yield of 70%.

^{1}H-NMR (400 MHz, Methanol-d_4) δ = 7.13 (d, *J* = 1.8 Hz, 1H), 6.78 – 6.59 (m, 3H), 5.72 (dd, *J* = 15.0, 9.0 Hz, 1H), 5.58 – 5.44 (m, 1H), 5.11 (q, *J* = 2.8, 2.0 Hz, 2H), 4.69 (dt, J = 12.0, 2.3 Hz, 1H), 4.22 (td, J = 10.0, 4.4 Hz, 1H), 4.08 – 3.95 (m, 3H), 3.96 – 3.88 (m, 1H),3.87 – 3.46 (m, 27H), 3.41 – 3.35 (m, 5H), 3.30 – 3.14 (m, 5H), 3.04 – 2.87 (m, 8H), 2.87 – 2.62 (m, 4H), 2.56 – 2.45 (m, 4H), 2.22 – 2.12 (m, 1H),1.71 (s, 3H), 1.62 – 1.43 (m, 3H), 1.33 (dd, *J* = 6.8, 1.8 Hz, 3H), 1.26 (dd, *J* = 6.4, 1.9 Hz, 3H), 0.87 (d, *J* = 1.9 Hz, 3H). ^{13}C-NMR (101 MHz, Methanol-d_4) δ = 173.2, 172.2, 171.6, 170.8, 169.8, 156.1, 153.9, 141.7, 141.5, 139.1, 133.2, 128.2, 125.3, 121.8, 118.0, 113.7, 93.9, 93.9, 88.5, 80.3, 78.1, 74.3, 73.2, 72.7, 72.5, 71.9, 71.8, 71.6, 70.6, 70.5, 70.2, 70.1, 70.1, 69.9, 69.9, 69.1, 67.5, 66.9, 61.3, 60.0, 55.9, 55.6, 52.5, 45.9, 39.9, 39.1, 38.2, 36.5, 36.1, 35.1, 35.0, 33.1, 32.7, 32.0, 29.7, 14.3, 13.4, 12.3, 11.2. HR-MS (ESI+): m/z calc. for $[M + Na]^+$: 1434.5209, found: 1434.5209.

Synthesis of trehalose-BODIPY (13) and trehalose-I-BODIPY (1)

Synthesis of trehalose-BODIPY (**13**) and trehalose-I-BODIPY (**1**) was performed as described earlier.[6,7] Analytical data were in accordance to the literature.

<u>Trehalose-BODIPY (13):</u>

^{1}H-NMR (500.30 MHz, DMSO-d_6): δ = 2.39 (s, 6H, C-10’-H_3, C-10’’-H_3), 2.42, (m, 2H, 2’-H), 2.45 (s, 6H, C-7’-H_3, C-7’’-H_3), 2.93-2,98 (m, 1H, 10-H), 3.05-3.27 (m, 6H, 3’-H (2H), 12_A-H (1H), three other sugar protons), 3.44-3.50 (m, 1H, 6_A-H), 3.50-3.60 (m, 4H, 6_B-H, 12_B-H, two other sugar protons), 3.63-3.68 (m, 1H), 3.71-3.76 (m, 1H, 11-H), 4.29 (t, 1H, *J* = 5.60 Hz, 6-OH), 4.60-4.66 (m, 2H), 4.75 (d, 2H, *J* = 4.44 Hz), 4,83 (d, 1H, *J* = 4.40 Hz), 4.85-4.90 (m, 3H, 1-H, 7-H, one other sugar proton), 6.23 (s, 2H, 8’-H, 8’’-H), 7.94 (t, 1H, *J* = 5.66 Hz, N-H). ^{13}C-NMR (125.80 MHz, DMSO-d_6) δ = 14.1 (2C, C-10’, C-10’’), 16.0 (2C, C-7’, C-7’’), 24.2 (1C, C-3’), 36.1 (1C, C-2’), 39.8 (1C, C-12), 60.8 (1C, C-6), 70.1 (1C), 70.7 (1C), 71.5 (1C), 71.6 (1C), 72.5 (1C), 72.5 (1C), 72.8 (1C), 93.3 (1C, C-1 or C-7), 93.5 (1C, C-1 or C-7), 121.7 (2C, C-8’, C-8’’), 130.6 (2C, C-5’, C-5’’), 141.1 (2C, C-6’, C-6’’), 145.8 (1C,

C-4’), 153.3 (2C, C-9’, C-9’’), 170.4 (1C, C-1’). ^{11}B-NMR (160.52 MHz, $CDCl_3$): δ = 0.39 (t, 1B, $J_{1,2}$ = 32.39 Hz). ^{19}F-NMR (470.75 MHz, $CDCl_3$): δ = −143.86 (m, 2F). HR-MS: calc. ($C_{19}H_{28}O_{13}S\ Cl^-$): 666.2616, found (ESI−): 666.2632. Purity HPLC pure.

Trehalose-I-BODIPY (1):

^{1}H-NMR (500.30 MHz, DMSO-d6): δ = 2.40-2.47 (m, 2H, 2’-H), 2.50 (s, 6H, C- 10’-H3, C-10’’-H3), 2.52 (s, 6H, C-7’-H3, C-7’’-H3), 2.93-2.99 (m, 1H, 10-H), 3.04-3.15 (m, 2H), 3.20-3.28 (m, 4H), 3.44-3.60 (m, 5H, 6A-H, 6B-H, 12B-H, two other sugar protons), 3.64-3.69 (m, 1H), 3.71-3.76 (m, 1H, 11-H), 4.28 (t, 1H, *J* = 5.63 Hz, 6-OH), 4.63-4.66 (m, 2H), 4.74 (d, 1H, *J* = 5.43 Hz), 4.76 (d, 1H, *J* = 4.94 Hz), 4.82 (d, 1H, *J* = 4.74 Hz), 4.86-4.89 (m, 3H, 1-H, 7-H, one other sugar proton), 7.92 (t, 1H, *J* = 5.74 Hz, N-H). ^{13}C-NMR (125.80 MHz, DMSO-d6) δ = 15.9 (2C, C-10’, C-10’’), 18.6 (2C, C-7’, C-7’’), 24.4 (1C, C-3’), 33.3 (1C, C-2’), 39.8 (1C, C-12), 60.8 (1C, C-6), 70.1 (1C), 70.7 (1C), 71.5 (1C), 71.5 (1C), 71.6 (1C), 72.5 (1C), 72.8 (1C), 87.5 (2C, C-8’, C-8’’), 93.4 (1C, C-1 or C-7), 93.5 (1C, C-1 or C-7), 130.7 (2C, C-5’, C-5’’), 143.1 (2C, C-6’, C-6’’), 146.0 (1C, C-4’), 154.6 (2C, C-9’, C-9’’), 170.2 (1C, C-1’). ^{11}B-NMR (160.52 MHz, DMSO-d6): δ = 0.24 (t, 1B, *J*1,2 = 32.04 Hz). ^{19}F-NMR (470.75 MHz, DMSO-d6): δ = −143.33 (m, 2F). HR-MS: calc. ($C_{28}H_{38}BF_2I_2N_3O_{11}\ Na^+$): 918.06, found (ESI+): 918.05. Purity >91%.

Biology

Bacterial strains and growth conditions

All necessary safety documentation and approvals for handling *M. bovis* BCG and *M. smegmatis* were secured from the relevant authorities. Experimental work involving this strains was conducted under Biosafety Level 2 (BSL-2) containment conditions, adhering to established safety protocols. The strains were routinely cultured in 7H9 broth supplemented with 10% (v/v) OADC, which contains 20 g L^{-1} glucose monohydrate, 50 g L^{-1} albumin, and 8.5 g L^{-1} sodium chloride, 0.5 g L^{-1} oleic acid and 40 mg L^{-1} catalase, along with 0.05% (v/v) Tween 80. For primary bacteria cultures 15 mg mL^{-1} cycloheximide and 50 mg mL^{-1} carbenicillin were added. In case of BCG::GFP additionally 50 mg mL^{-1} kanamycin was added. Chemicals and media components were purchased from Carl Roth GmbH + Co. KG, BLDpharm®, VWR International, LLC, Thermo Fisher Scientific Inc. and Sigma-Aldrich® – Merck KGaA.

Mammalian cells and growth conditions

Early passage HT-1376 or T24 cells were grown in T-25 or T-75 flasks in EMEM medium with 10% FBS or DMEM/F12 medium with 10% FBS and 1% L-Glutamine solution containing

1000 U mL^{-1} Penicillin-Streptomycin till they reached ~80% confluency (37 °C humidified incubator, 5% CO_2). For assays the same media compositions without Penicillin-Streptomycin solution were used. Media and ELISA kits were purchased Gibco Thermo Fisher Scientific, Thermo Fisher Scientific, ATTC, Cytiva, ImmunoTools GmbH and InvivoGen.

**Name**	**Source**
M. smegmatis mc^{2}155	Gift from Laboratory of Dr. William R. Jacobs Jr., Albert Einstein College Medicine, England
M. bovis BCG Tice	ATCC-35743, TIMC 1028 BCG Tice
M. bovis BCG::GFP	Robert Koch Institute (Berlin, Germany)
HT-1376	ATCC catalog CRL-1472
T24	Obtained from the cell bank at BIOSS (Center for Biological Signalling Studies) University of Freiburg, Germany
BMDMs	Isolated from mice (27 weeks old male C57BL6/N). Animals were euthanized in accordance with the respective European, federal, and local regulations and were approved by the Regierungspräsidium Freiburg (X-19/02A).
pET23b-fbpA, pET23b-fbpB	Gift from the Laurent Kremer Group[8] Université de Montpellier, France

MIC assay against *M. bovis* BCG

The MIC values were determined by performing a dilution series of the tested substance in 96-well plate format. Compound stocks were prepared to 5 mM in dimethyl sulfoxide and stored at −20 °C. Trehalose-PEG$_4$-mertansine **(2)** and SQ109 as control were each diluted in duplicates from a final concentration of 200 µM to 1.56 µM in growth medium (7H9 with 10% OADC and 0.05% Tween 80) in a 96-well round-bottom plate. Subsequently, 150 µL and 100 µL of medium were added to columns 2 and 11, respectively. Next, 50 µL of a previously prepared bacterial culture was added to a final OD of 0.1 and the plate was incubated sealed for 24 h at 37 °C. The plate was centrifuged at 3486 xg and 4 °C for 6 min, the medium was removed and bacteria were washed twice with 150 µL of 1x phosphate buffered saline (PBS) containing 10% dimethyl sulfoxide, after which the plate was also centrifuged at 3486 x g and 4 °C for 6 min. Then 150 µL of fresh medium was added and incubated for another 24 h at 37 °C. Finally, 20 µL of resazurin solution (0.15 mg mL^{-1}) was added in each well and incubated for 16 h. The test was evaluated by absorbance measurement at 570 nm and 600 nm with a plate reader

(Tecan® SPARK 10M). The raw date was analyzed using the Alamar Blue assay protocol from Thermo Fisher. The processed and baseline corrected data were then plotted and analyzed via Dose Response Fit using Origin Pro 2025.

Mertansine release from trehalose-PEG$_4$-mertansine (2) with dithiothreitol (DTT)

Trehalose-PEG$_4$-mertansine **(2)** (100 µM) was incubated with 10 mM DTT, dissolved in PBS at pH 7.6, at 37 °C for 30 min. The reaction, as well as the control – mertansine in PBS without DTT – were analyzed by HPLC (Hypersil Gold C_{18}, 150 x 3 mm, 3 µm). For HPLC analysis a solvent mixture of ultra-pure water (A) and acetonitrile (B) was used, and the following elution gradient. Isocratic at 5% B for 2 min, followed by a linear gradient of 5% to 95 % B over 10 min, and finally 95% solvent B for 3 min.

Medium stability assay of trehalose-PEG$_4$-mertansine (2)

100 µM Trehalose-PEG$_4$ mertansine (**2**) was incubated in fully supplemented EMEM and DMEM/F12 for 24 h. After 1 h, 6 h and 24 h, an aliquot of 50 µL was diluted with 50 µL methanol and analyzed by HPLC using a Waters XSelect Premier CSH C18 VanGuard FIT Column, 130 Å, 2.5 µm, 2.1 x 50 mm column. For elution a solvent mixture of ultra-pure water (A) and acetonitrile (B) was used, described by the following gradient. Isocratic at 5% B for 0.25 min, followed by a linear gradient from 5% to 100 % B over 3.5 min, and finally 100% B for 1 min.

IC_{50} assay against HT-1376 and T24 cell lines

Cells were seeded in 96-well flat-bottom plates (HT-1376 with 4000 cells well^{-1} and T24 with 1100 cells well^{-1}) in a volume of 200 µL. To allow attachment of the cells, they were cultured for 24 h at 37 °C and 5% CO_2. After 24 hours, the medium was removed and the cells were washed with medium. A dilution series of the compounds was prepared in a separate 96-well plate using fresh cell culture medium, here initial concentrations of each compound (varies for different compounds and cell lines) were diluted sequentially. In columns 2 and 11 only 150 µL medium were prepared. Subsequently, the dilution series was applied to the pre-seeded cells. After 48 h of incubation at 37 °C and 5% CO_2, the medium was removed. The cells were washed twice with 100 µL PBS and 120 µL fresh medium containing 0.5 mg mL^{-1} 3-(4,5-Dimethylthiazol-2-yl)-2,5-diphenyltetrazoliumbromid (MTT) was added in each well. After 4 h incubation at 37 °C and 5% CO_2, the medium was removed carefully and the generated formazan crystals were dissolved in 120 µL dimethyl sulfoxide and the absorbance at 595 nm was measured with a plate reader (Tecan® SPARK 10M) for analysis of the cell viability.

The resulting data were baseline corrected using the blank control, normalized to the live control (no compound), and plotted against the corresponding concentrations (logarithmic). The resulting data sets were analyzed using Dose Response Fit (Origin Pro 2025) and IC_{50} values were determined. For HT-1376 and compound 2, the values for log concentration 0.1938 and −2.214 were not considered for IC_{50} determination. For T24 and compound **2**, the values for log concentration −2.214 and −3.720, and for mertansine the values at −1.61 and −1.91 were not considered for IC_{50} determination. The values differed significantly from the surrounding data and were not included in the dose-response fit because the fit could not be processed if the outlayers were respected.

Lipid extraction of labeled *M. bovis* BCG

Labeled lipids were extracted using a lipid extraction protocol adapted from Dutta *et al.* (2019) and G. Degiacomi *et al.* (2017).[7,9] For this purpose three cultures of 4 mL *M. bovis* BCG, each, were incubated at 37 °C and 150 rpm (New Brunswick shaking incubator) for 24 h with 50 µM trehalose-BODIPY (**13**) at an optical density (OD) of 0.1. Subsequently, the bacteria were centrifuged at 7,500 xg at 4 °C for 6 min and washed with 4 mL PBS containing 10% dimethyl sulfoxide. This washing step was pursued twice, to remove unbound **13**. After washing, the bacterial pellets were extracted once with 900 µL methanol and chloroform in a ratio of 2:1 at 56 °C and 600 rpm (Eppendorf Thermoblock) for 24 h. After incubation the tubes were centrifuged at 4 °C and 17,000 xg for 6 min. The supernatant was collected in fresh tubes and the pellet extracted twice with 900 µL methanol and chloroform in a ratio of 1:2 at 56 °C and 600 rpm for 4 h respectively 2 h. The combined supernatants were evaporated and cleaned by biphasic washing with chloroform, methanol and water in a ratio of 4:2:1. The organic phases were dried at 56 °C and 600 rpm. The dry lipids were dissolved in 100 µL dimethyl sulfoxide and the fluorescence intensity of all samples was determined using a plate reader (Tecan® SPARK 10M, excitation wavelength: 470 nm, emission wavelength: 514 nm). The amount of incorporated Trehalose-BODIPY **(13)** was calculated using a calibration curve of dilutions of Trehalose-BODIPY (**13**). Therefore, the data was plotted against the concentration and a linear regression was performed using Origin Pro 2025. This curve was used for calculation of the concentration of incorporated compound.

Lipid extraction of *M. smegmatis* for linker length screening

This assay was performed using the same lipid extraction protocol as used for BCG, adapted from G. Degiacomi *et al.* (2017).[9] After the lipid extraction the dry lipids were dissolved in a mixture of chloroform and methanol (1:1) and analyzed by TLC (solvent: 20:4:0.5,

chloroform:methanol:water). TLC plates were scanned by fluorescence scanning on a gel imager (Vilber Lourmat, Fusion SL) with an emission wavelength of 590 nm or stained with $CuSO_4$ (10% $CuSO_4$ in 8% phosphoric acid solution) followed by heating.

Lipid analysis by TLC assay

a) Without addition of DTT: Pre-cultures of *M. smegmatis* (5 mL) were grown in 7H9 broth supplemented with 0.05% (v/v) Tween 80 from a single colony and harvested by centrifugation after two days' culture. Cells were diluted in culture broth to an OD_{600} of 0.1 and incubated with the fluorophore conjugates or the control compound trehalose-I-BODIPY **(1)** (10 µM) for 6 h and harvested. Afterwards, the cell pellets were washed twice with ultra-pure water to remove any nonspecifically bound compound. Subsequently, the lipids were extracted with chloroform/methanol (1:2 and 2:1), resolved by thin-layer chromatography (TLC) using chloroform/methanol/water (20:4:0.5) as solvent. TLC plates were scanned by fluorescence scanning on a gel imager (Vilber Lourmat, Fusion SL) with an emission wavelength of 590 nm.

b) With addition of DTT: Briefly, pre-cultures of *M. smegmatis* (5 mL) were grown in 7H9 broth supplemented with 0.05% (v/v) Tween 80 from a single colony and harvested by centrifugation after two days' culture. Cells were diluted in culture broth to an OD_{600} of 0.1 and incubated with fluorophore conjugates or the control compound trehalose-I-BODIPY **(1)** (10 µM) for 6 h and harvested. The cell pellets were washed twice with ultra-pure water to remove any nonspecifically bound compound. Then the cell pellets were resuspended in PBS buffer, incubated with or without DTT for 30 min. Afterwards, the cell pellets were centrifuged and washed with water three times, extracted with chloroform/methanol (1:2 and 2:1), resolved by thin-layer chromatography (TLC) using chloroform/methanol/water (20:4:0.5) as solvent. TLC plates were scanned by fluorescence scanning on a gel imager (Vilber Lourmat, Fusion SL) with an emission wavelength of 590 nm.

Purification of Ag85 enzymes

The Ag85 enzymes were expressed in *E. coli* BL21 Star. 5 mL of preculture (in LB medium) was used to inoculate 2 L of autoinduction medium. The culture was incubated at 24 °C for 18 h at 150 rpm. Cells were harvested at 3320 xg for 90 min at 4 °C and lysed with lysis buffer (20 mM NaH_2PO_4, 500 mM NaCl, 20 mM Imidazol, 1 mg mL^{-1} Lysozym, 1 mM PMSF, 1 mM DTT, 1 U mL^{-1} DNaseI, pH 7.4) and sonication (4 x 3 min with 40% amplitude, 1 min break, each). Cells were then centrifuged at 8000 xg at 4 °C for 15 min and sonicated again. The combined lysate was centrifuged at 15000 xg for 60 min at 4 °C. The enzymes were purified using a His-trap HP (Cytiva) with a step gradient at a flow rate of 1 ml min^{-1}: 15 CV washing

with binding buffer (20 mM Na_3PO_4, 500 mM NaCl, 20 mM Imidazol), followed by a stepwise gradient (5 CV 25% elution buffer (20 mM NaH_2PO_4, 500 mM NaCl, 500 mM Imidazol), 50% elution buffer, 100% elution buffer). The enzymes were dialyzed into a 200 mM Tris, 300 mM NaCl buffer. The pre-purified enzymes were then polished by size exclusion chromatography (HiLoad 16/600 Superdex 200pg, flow rate 0.5 ml min^{-1}). Enzymes were concentrated to 1.3 mg mL^{-1} (Ag85A) and 3.6 mg mL^{-1} (Ag85B) using a Vivaspin concentrating column 10.000 MWCO.

Ag85 *in vitro* processing assay

An assay mixture of 100 µL was set up containing either trehalose-PEG_4 mertansine (**2**), or trehalose-I-BODIPY **(1)** in PBS in the presence of either 8 µM Ag85A or Ag85B and 200 µM trehalose-hexadecanoate. The mixture was incubated for 2 h at 37 °C. After the incubation, 50 µL of the reaction mixture was diluted with 50 µL of methanol, cooled on ice and 10 µL of each reaction was analyzed by TLC (solvent: 20:4:0.5, chloroform:methanol:water). TLC plates were prepared in duplicate, one of which was stained with vanillin-H_2SO_4 (52 g L^{-1} dissolved in a mixture of ethanol and 3 M H_2SO_4, 87:13) the other one was used for mass spectrometry.

Cell inactivation assay with labeled bacteria

120,000 HT-1376 and 33,000 T24 cells per well were seeded in a 6-well plate and incubated for 24 h at 37 °C and 5% CO_2, respectively. Two plates per condition were prepared, one in which cells were seeded to all six wells and one were cells were only seeded in four wells, whilst two wells were filled with medium only. In parallel, 3.5 mL of BCG at OD 0.1 per replicate and compound trehalose-PEG_4 mertansine (**2**) was incubated for 24 h at 37 °C at 150 rpm, in a final concentration of 50 µM of each compound. The medium was removed and the bacteria were washed twice with 1 mL PBS containing 10% dimethyl sulfoxide, centrifuged at 7500 xg and 4 °C for 6 min, each. Subsequently, the bacterial pellet was resuspended in 1 mL of fresh medium (EMEM with 10% FBS or DMEM/F12 with 10% FBS and 1% 200 mM L-Glutamine solution). The medium in the 6-well plates was removed after 24 h and the labeled and washed bacteria were applied to the cells at the corresponding MOI's of 200, 100, 50 and 10. In the control assays the free cytostatic agent was added to the wells directly after the non-labeled bacteria were added, wild-type bacteria only were added or no bacteria were added. For HT-1376, 9.5 pmol, 4.7 pmol, 2.4 pmol and 0.47 pmol compound were added at the corresponding MOIs of 200, 10, 50 and 10, respectively. In case of T24, 2.6 pmol, 1.3 pmol, 0.65 pmol and 0.13 pmol compound were added at the corresponding MOIs of 200, 10, 50 and 10, respectively. The cells were incubated for another 24 h at 37 °C and 5% CO_2, after which

the medium was removed, and the cells were washed twice with 1 mL PBS and 1.5 mL fresh medium was added. After 48 h of incubation at 37 °C and 5% CO_2, the medium was removed and the cells were washed again twice with PBS. 300 µL crystal violet solution (0.5% in 25% methanol) was added to the cells and incubated for 30 min at room temperature. The dye was removed and the cells were washed three times with 8 mL ultrapure water, each and dried at 37 °C. 1 mL ethanol per well was used to solubilize the incorporated dye, and undissolved cells were scraped off with a scraper. 100 µL of the supernatant was transferred in triplicate to a 96-well plate and the absorbance was measured at 595 nm to evaluate the assay using the plate reader. Blasticidin S at concentrations of 100 µM, 50 µM, 25 µM, 12.5 µM, 6.25 µM was used as a control. The results were baseline corrected using the blank controls, normalized to the live control (no bacteria/compound), and plotted against the MOIs used using Origin Pro 2025's paired comparison plot function to calculate statistical significance.

IL-6 and TNF-α ELISA assay of BCG infected BMDMs

The bone marrow-derived macrophages (BMDMs) used in this assay were isolated and differentiated according to the protocol published by O. Gross (2015).[10] For the preparation of 1 mL BCG∗mertansine or BCG, see subsection "Cell inactivation assay with labeled bacteria". The washed BCG were resuspended in RPMI Medium 1640 containing GlutaMAX™-I, 10% fetal calf serum (FCS), and 100 ng mL^{-1} of recombinant human macrophage colony-stimulating factor (M-CSF). The medium of the previously seeded BMDMs, was removed and the cells were infected at MOIs of 1 or 5 with BCG and BCG∗mertansine for 24 h at 37 °C and 5% CO_2. As control three dilutions of *E. coli* K12 ultrapure lipopolysaccharide (LPS, InvivoGen), 100 ng mL^{-1}, 10 ng mL^{-1} and 1 ng mL^{-1} were applied. These controls were also incubated for 24 h at 37 °C and 5% CO_2. Subsequently the plate was centrifuged at 400 xg for 5 min at 4 °C, and the supernatants were transferred into a new 96 well plate. Each sample was then sterile-filtered into another 96 well plate. The ELISA assays for TNF-α and IL-6 were performed according to the manufacturer's protocols (Thermo Fisher Scientific, Invitrogen, Mouse TNF alpha Uncoated ELISA Kit (Catalog No. 88-7324-88) and Mouse IL-6 Uncoated ELISA Kit (Catalog No. 88-7064-88)). 100,000 BMDMs per well were seeded in a 96 well plate and incubated over night at 37 °C and 5% CO_2. The data points were recorded using a plate reader and analyzed using logarithmic standard curves generated with the ELISA standards provided in the kits (see Figure S7). The concentrations of TNF-α and IL-6 were plotted using Origin Pro 2025's paired comparison plot function to calculate statistical significance. The concentrations of the LPS controls, the control with only cells and medium,

as well as the ELISA blank (ELISA assay without sample) were plotted next to the paired comparison plot of the BCG samples using Origin Pro 2025.

Fluorescence microscopy of internalized bacteria

HT-1367 and T24 cells were seeded at different numbers of cells per well. For HT-1376, 25,000 and 12,500 cells per well were seeded into 8-well IBIDI slides (Ibidi GmbH). 25,000 cells were used for the overview images and 12,500 cells for the single cell images. For T24, 12,500 cells per well were seeded into 8-well IBIDI slides (Ibidi GmbH) and used for the overview images as well as for the single cell images. The cells were treated as above, except that for fluorescence microscopy the BCG bacteria were labelled with trehalose-BODIPY (**13**). BCG::GFP was used as a control. A MOI of 200 was used for HT-1376 and an MOI of 100 for T24 to obtain z-stack fluorescence microscopy images using a Zeiss LSM880 AiryScan. Before imaging the cells were fixed and stained. Cells were first stained with CellMask Orange™ by washing once with PBS and staining with a 1:1000 dilution (5 µg mL^{-1}) of a purchased dye stock solution (5 mg mL^{-1}) in PBS for 5 minutes at 37 °C and 5% CO_2. The cells were then washed once with PBS and fixed with 4% formalin solution in PBS for 20 minutes at rt. After fixation, the cells were washed once with PBS and stained with 2 mg mL^{-1} DAPI in PBS for 15 minutes at rt. Finally, the cells were washed once with PBS and stored in PBS at 4 °C until use.

Literature

[1] Y.-M. Wang, C.-Q. Zhou, J.-X. Chen, W.-H. Chen, *Bull. Korean Chem. Soc.* **2013**, *34*, 749.

[2] V. Cucinotta, A. Giuffrida, G. Maccarrone, M. Messina, A. Puglisi, G. Vecchio, *Electrophor.* **2007**, *28*, 2580.

[3] F. P. Rodriguez-Rivera, X. Zhou, J. A. Theriot, C. R. Bertozzi, *J. Am. Chem. Soc.* **2017**, *139*, 3488.

[4] M. Togashi, T. Terai, H. Kojima, K. Hanaoka, K. Igarashi, Y. Hirata, Y. Urano, T. Nagano, *Chem. Commun. (Cambridge, U. K.)* **2014**, *50*, 14946.

[5] R. S. Navath, Y. E. Kurtoglu, B. Wang, S. Kannan, R. Romero, R. M. Kannan, *Bioconjugate Chem.* **2008**, *19*, 2446.

[6] Sara Bonacchi, Marco Montalti, Luca Prodi, Nelsi Zaccheroni, Riccardo Juris, Damiano Genovese, Enrico Rampazzo, US9616141B2.

[7] A. K. Dutta, E. Choudhary, X. Wang, M. Záhorszka, M. Forbak, P. Lohner, H. J. Jessen, N. Agarwal, J. Korduláková, C. Jessen-Trefzer, *ACS Cent. Sci.* **2019**, *5*, 644.

[8] a) L. Kremer, W. N. Maughan, R. A. Wilson, L. G. Dover, G. S. Besra, *Lett. Appl. Microbiol.* **2002**, *34*, 233; b) A. Viljoen, M. Richard, P. C. Nguyen, P. Fourquet, L. Camoin, R. R. Paudal, G. R. Gnawali, C. D. Spilling, J.-F. Cavalier, S. Canaan, M. Blaise, L. Kremer, *J. Biol. Chem.* **2018**, *293*, 2755.
[9] G. Degiacomi, A. Benjak, J. Madacki, F. Boldrin, R. Provvedi, G. Palù, J. Kordulakova, S. T. Cole, R. Manganelli, *Sci. Rep.* **2017**, *7*, 43495.
[10] O. Gross, *Methods Mol. Biol.* **2012**, *844*, 199.

Appendix: Analytical Data (^{1}H-NMR, ^{13}C-NMR, ^{11}B, ^{19}F, 2D-NMR, HR-MS and HPLC Chromatograms)

Appendix

6-*p*-Toluenesulfonyl trehalose

^{1}H-NMR:

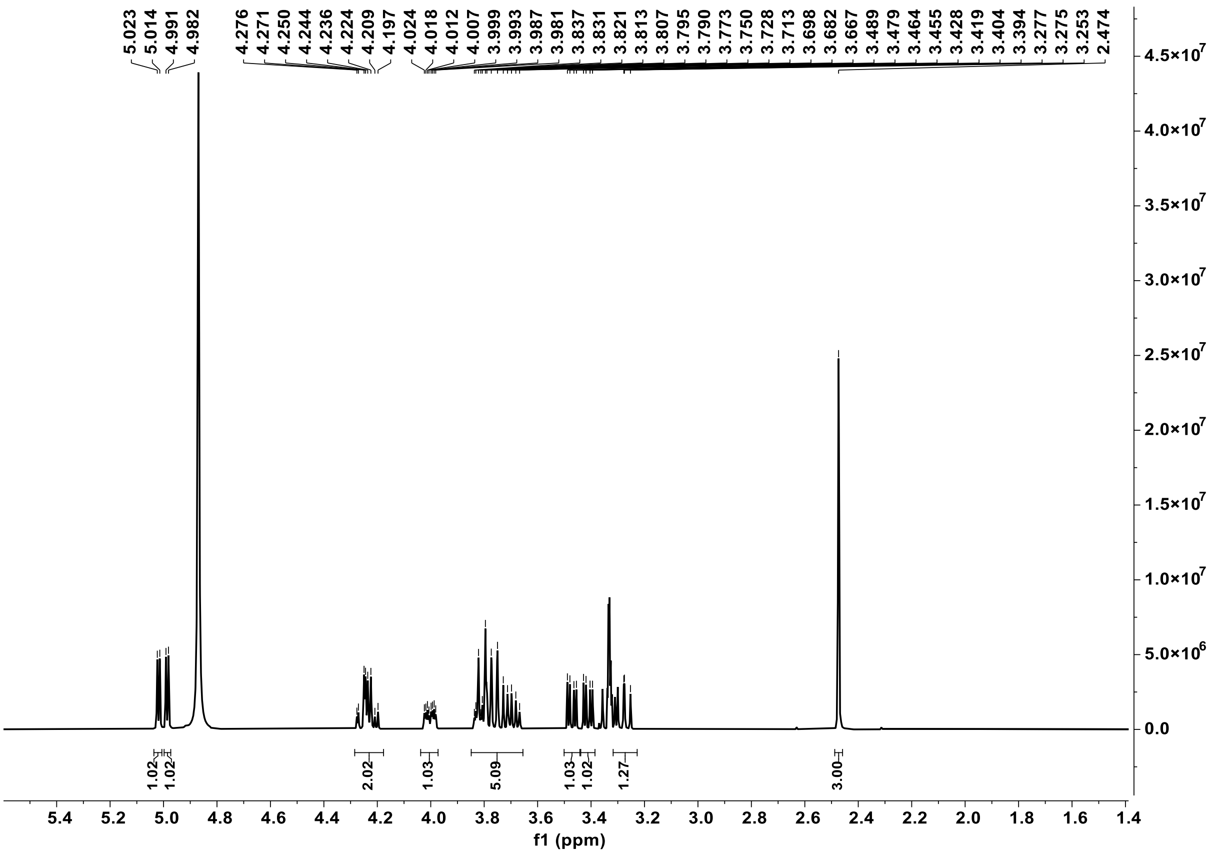

^{13}C-NMR:

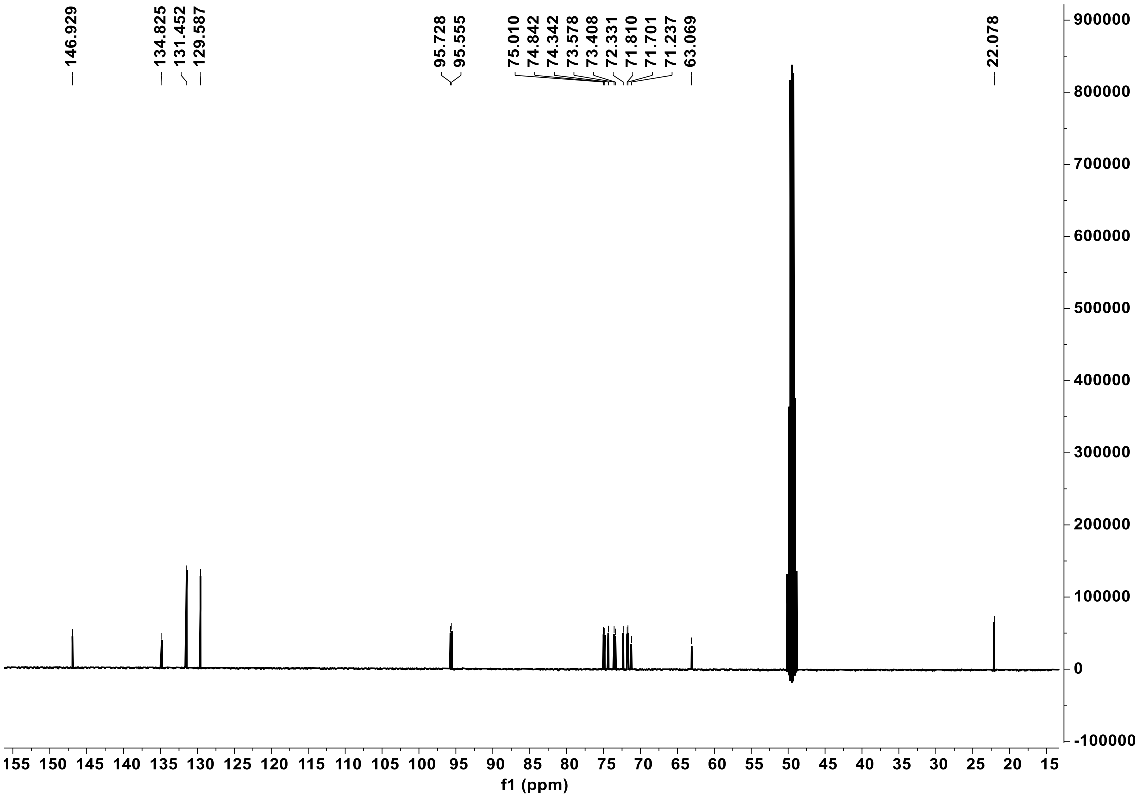

NOESI:

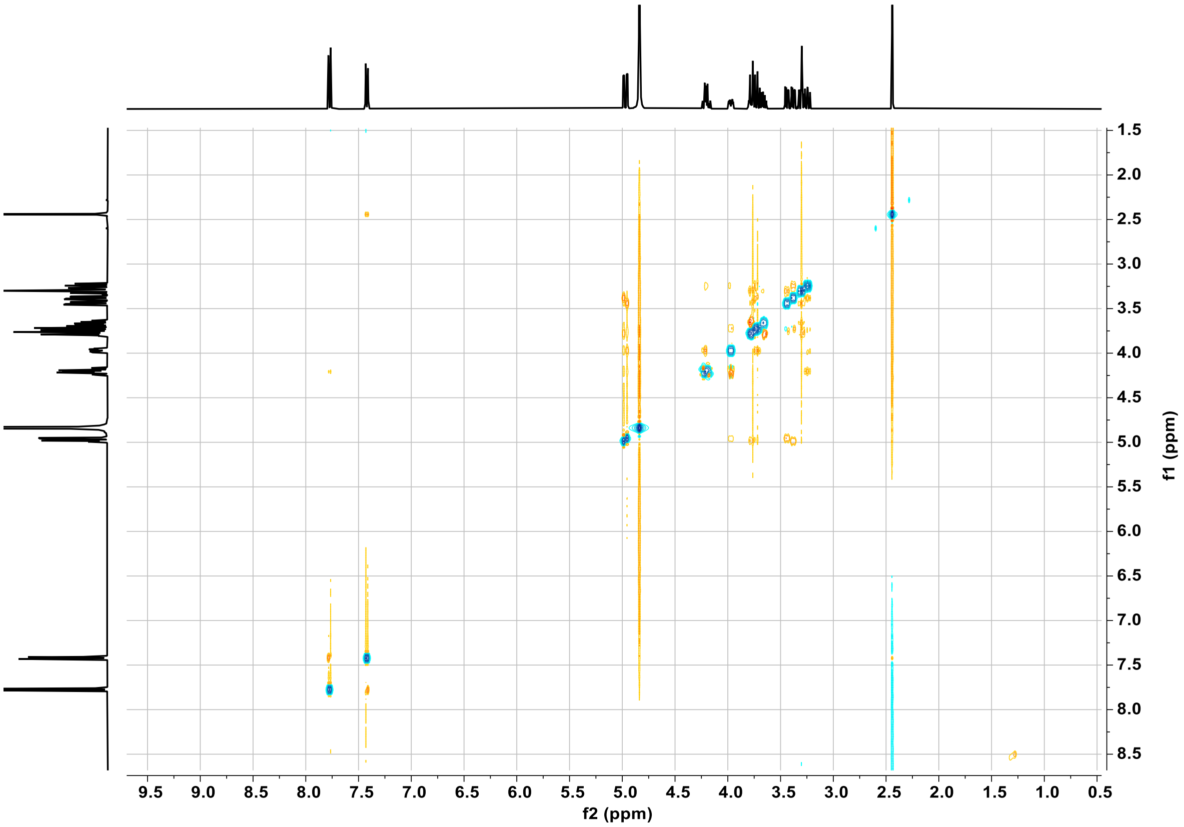

HR-MS:

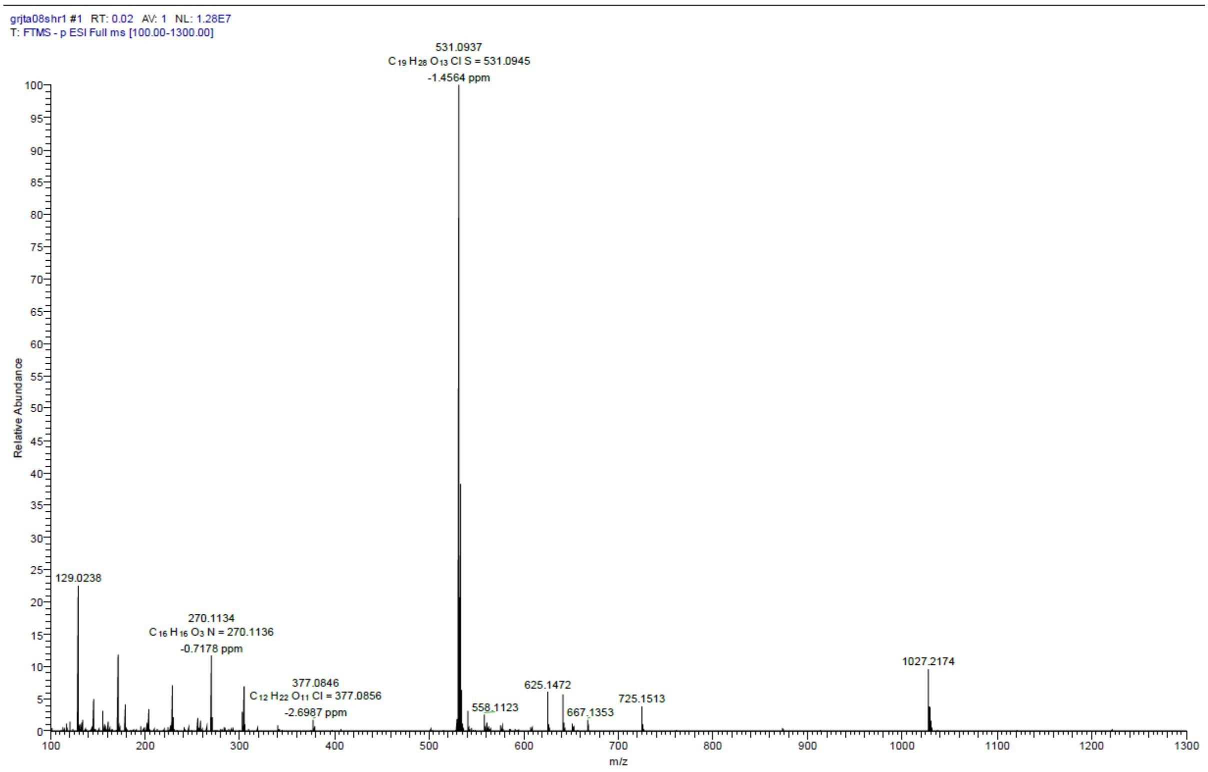

6-Azido-trehalose

^{1}H-NMR:

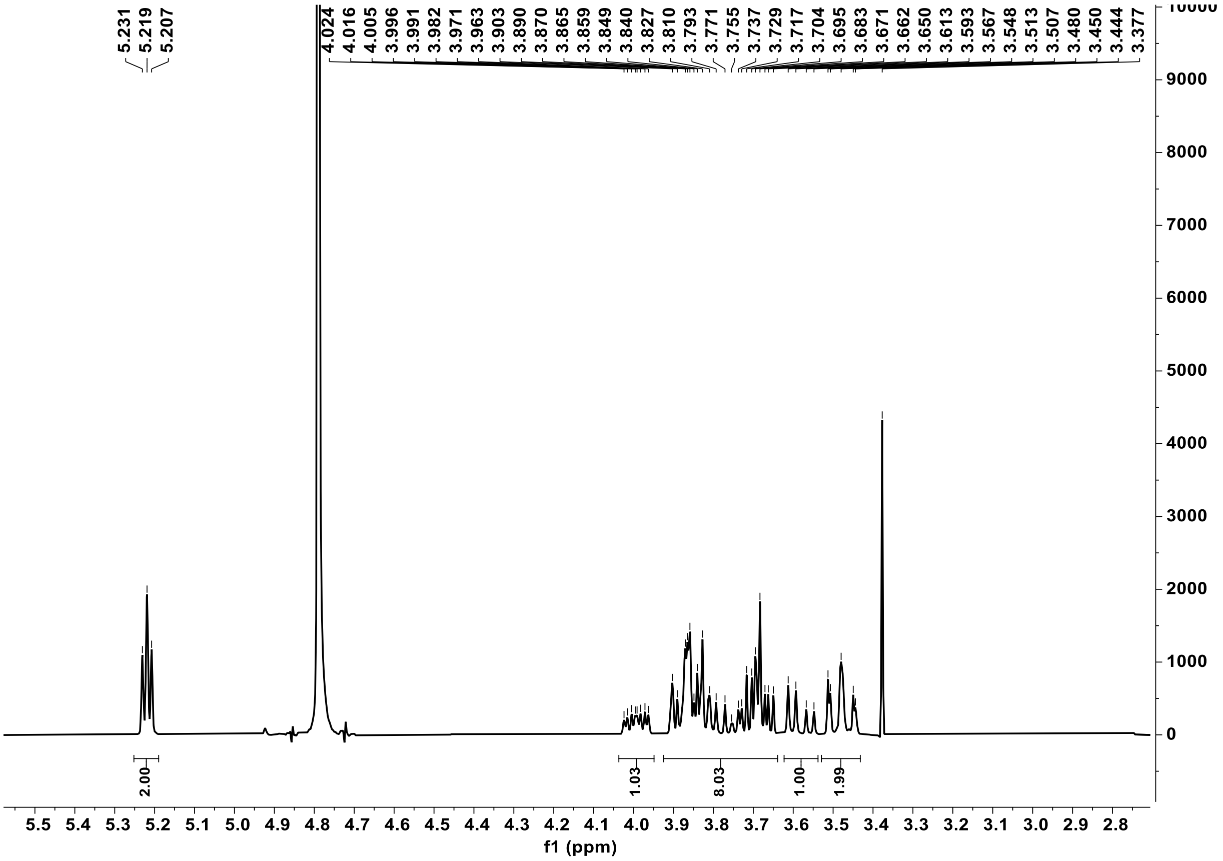

^{13}C-NMR:

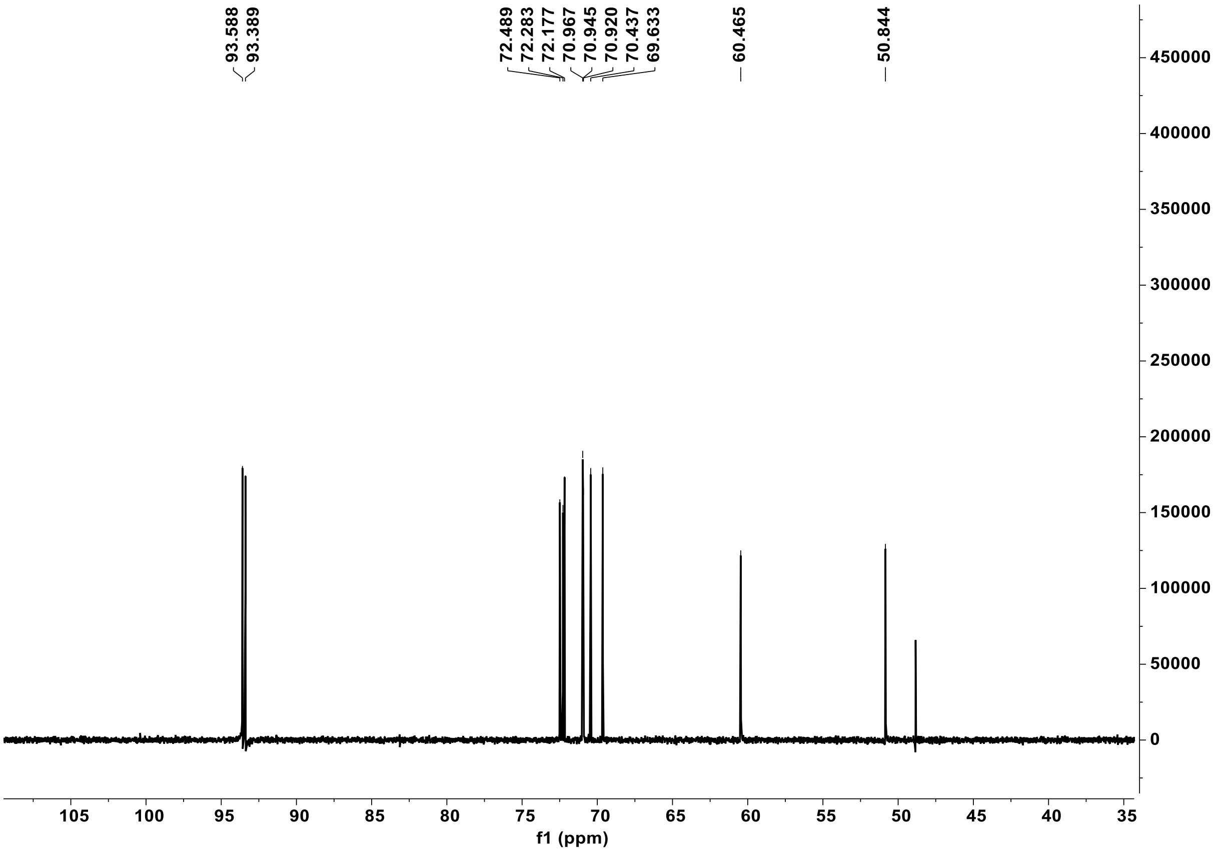
93.588
93.389
72.489
72.283
72.177
70.967
70.945
70.920
70.437
69.633
60.465
50.844
f1 (ppm)

HR-MS:

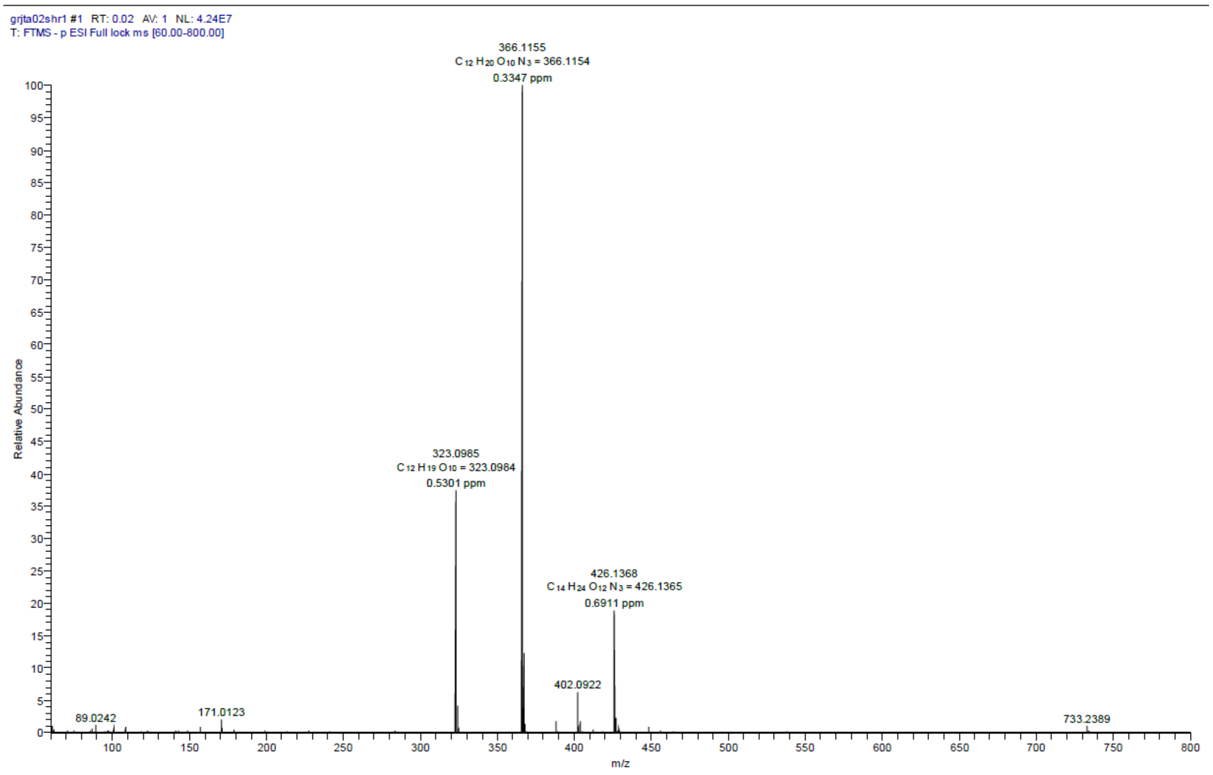
grjta02shr1 #1 RT: 0.02 AV: 1 NL: 4.24E7
T: FTMS - p ESI Full lock ms [60.00-800.00]
366.1155
C 12 H 20 O 10 N 3 = 366.1154
0.3347 ppm
323.0985
C 12 H 19 O 10 = 323.0984
0.5301 ppm
426.1368
C 14 H 24 O 12 N 3 = 426.1365
0.6911 ppm
402.0922
89.0242
171.0123
733.2389
Relative Abundance
m/z

6-Amino trehalose (6)

^{1}H-NMR:

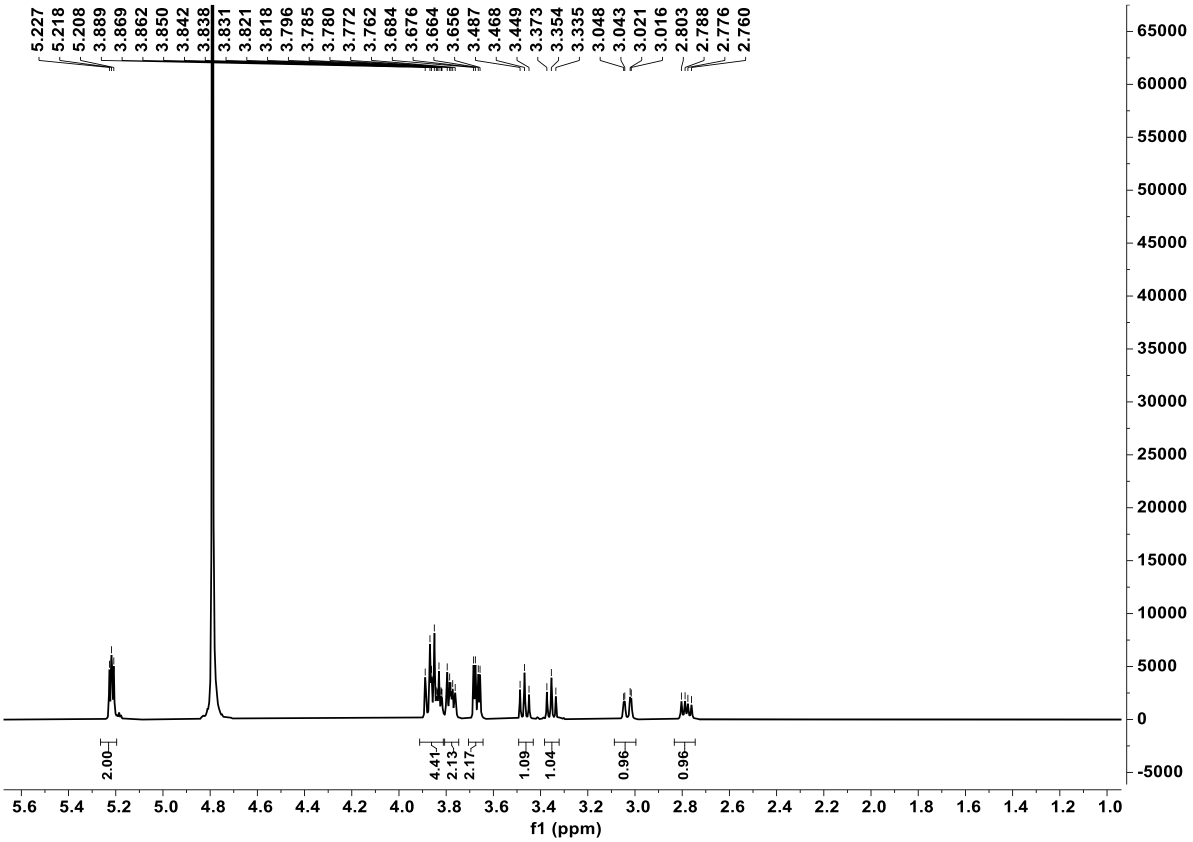

^{13}C-NMR:

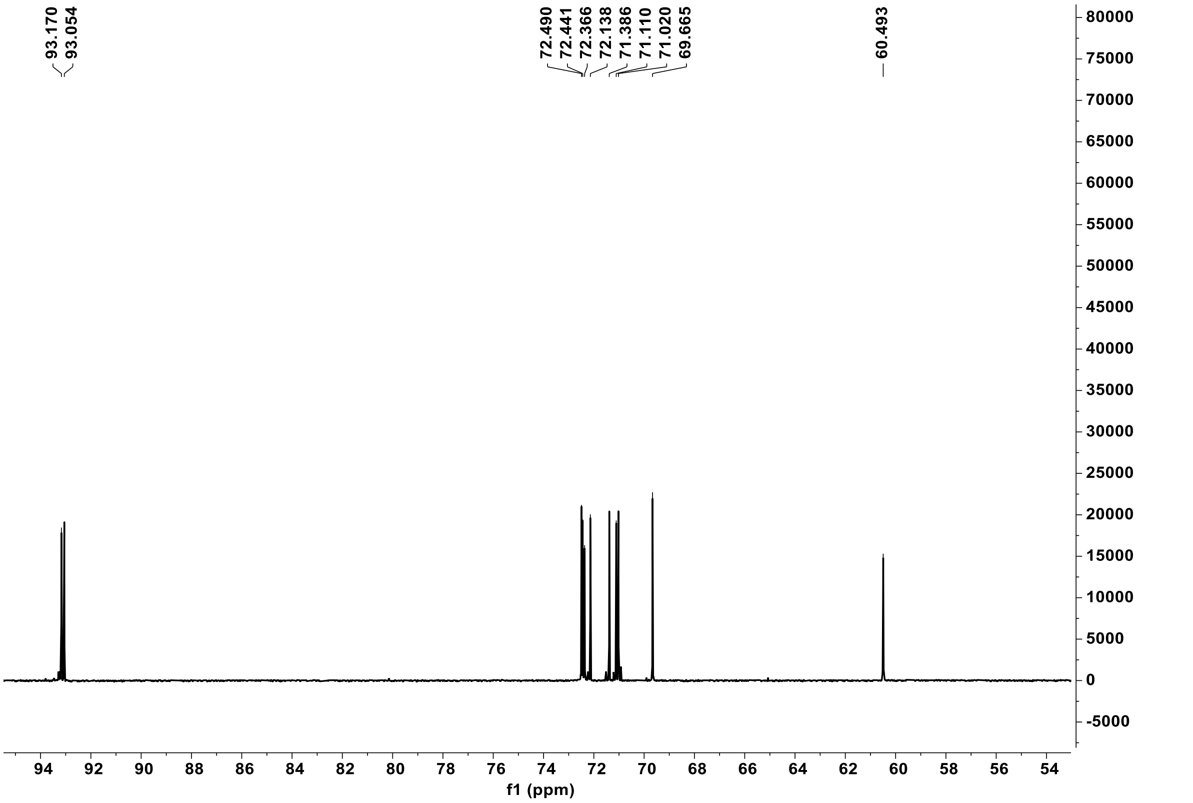

HR-MS:

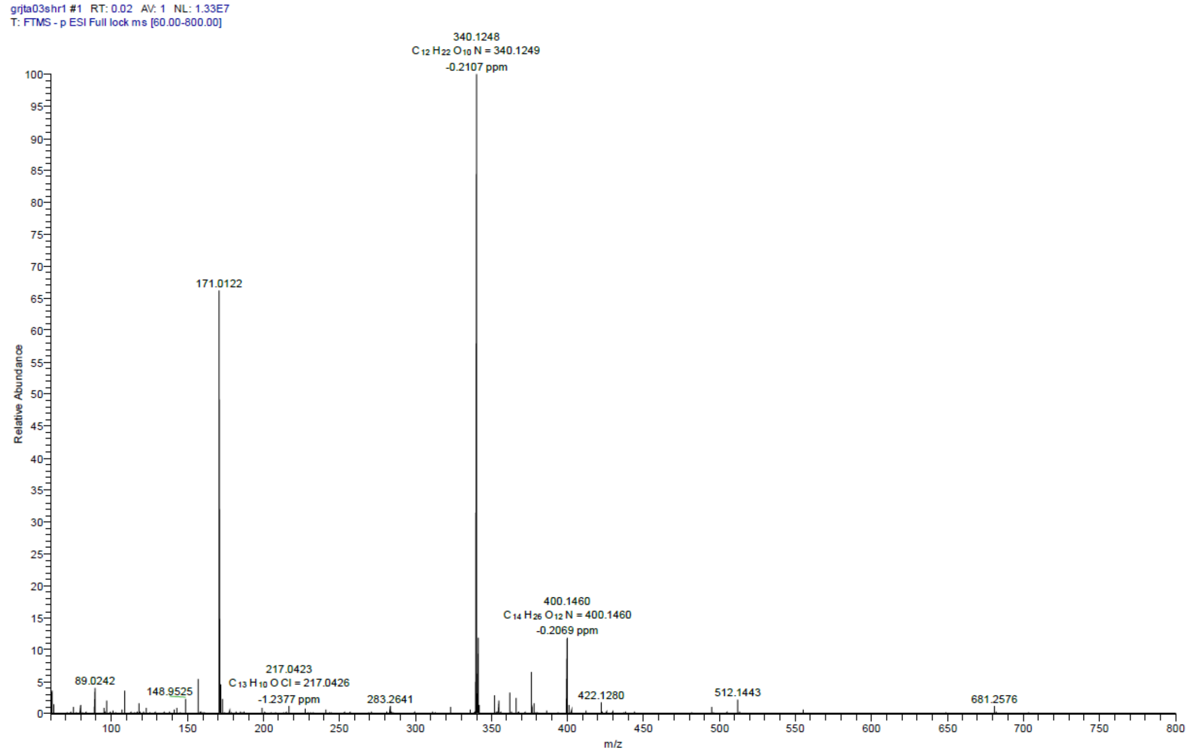

2-(Tritylthio)ethan-1-amine (3)

^{1}H-NMR:

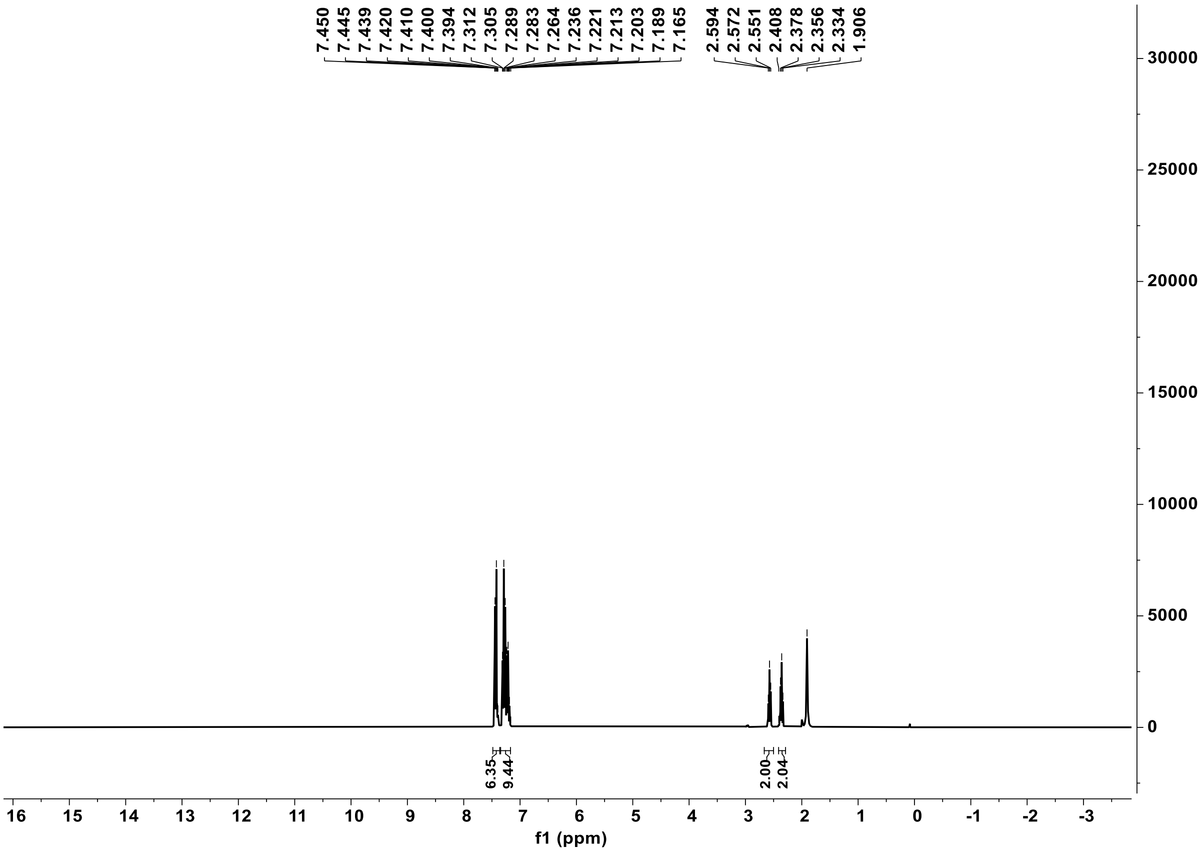

^{13}C-NMR:

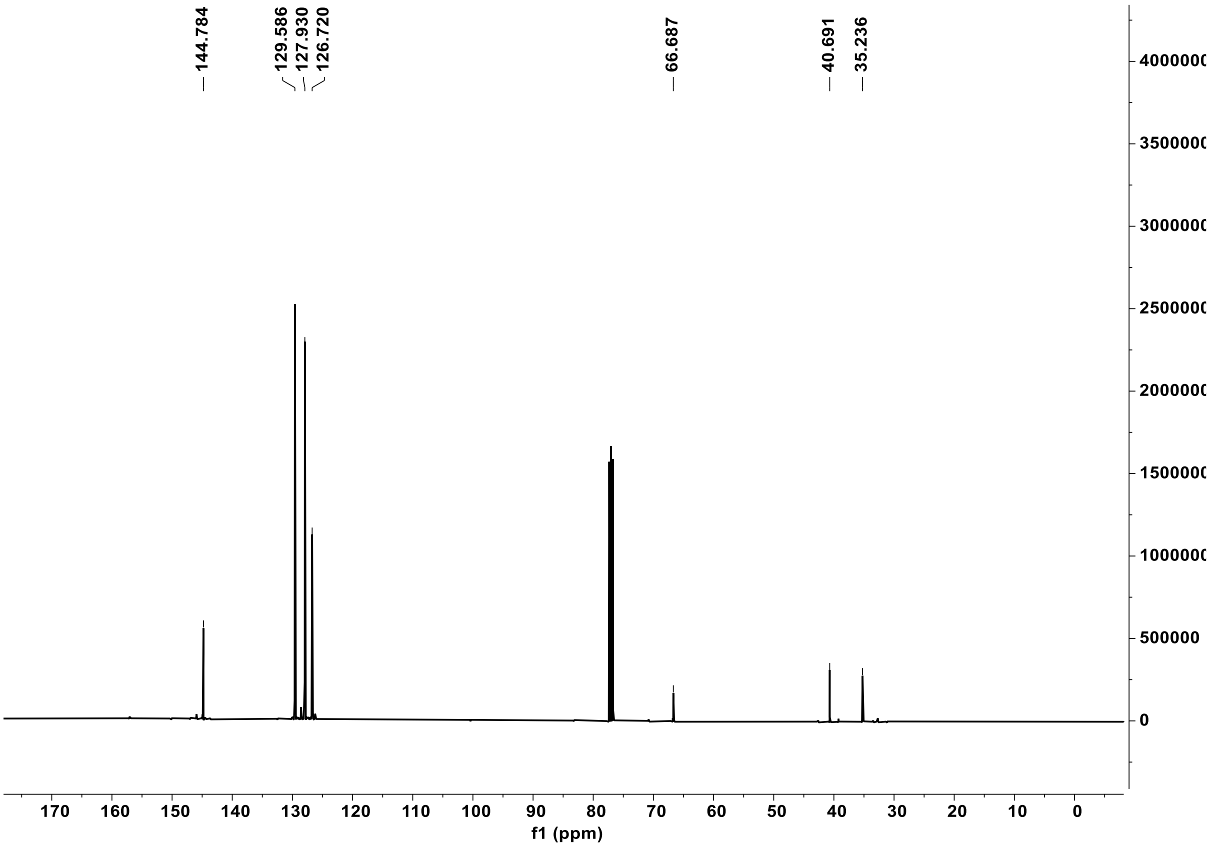

5-(2-Tritylthio-ethyl)carbamoyl fluorescein (4)

^{1}H-NMR:

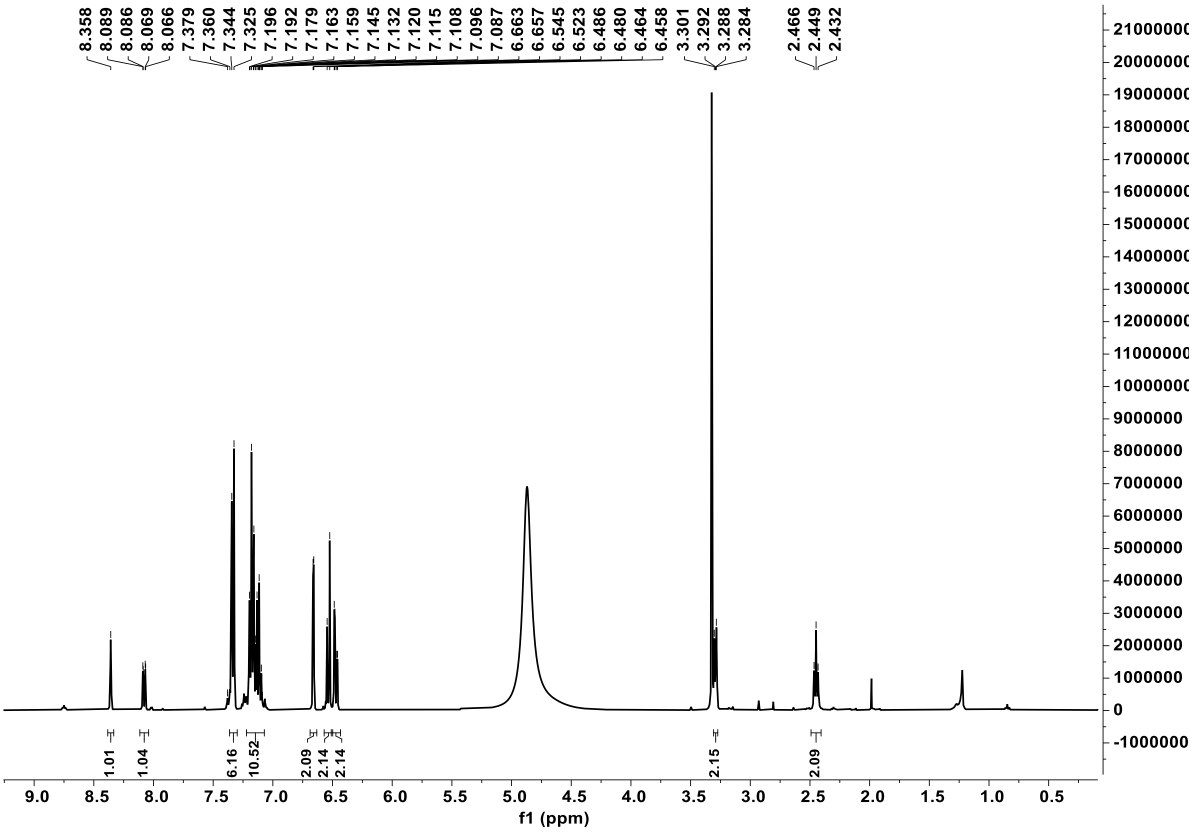

^{13}C-NMR:

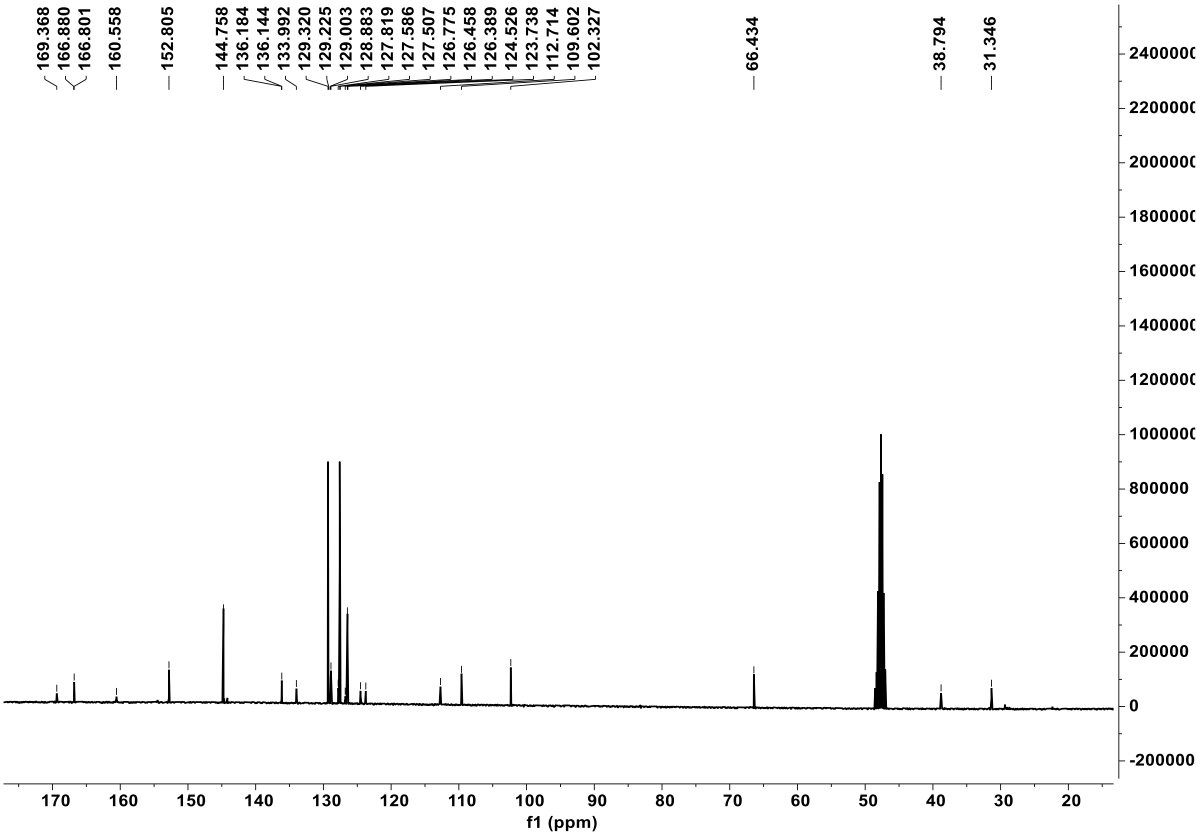

169.368
166.880
166.801
160.558
152.805
144.758
136.184
136.144
133.992
129.320
129.225
129.003
128.883
127.819
127.586
127.507
126.775
126.458
126.389
124.526
123.738
112.714
109.602
102.327
66.434
38.794
31.346
f1 (ppm)

HMBC:

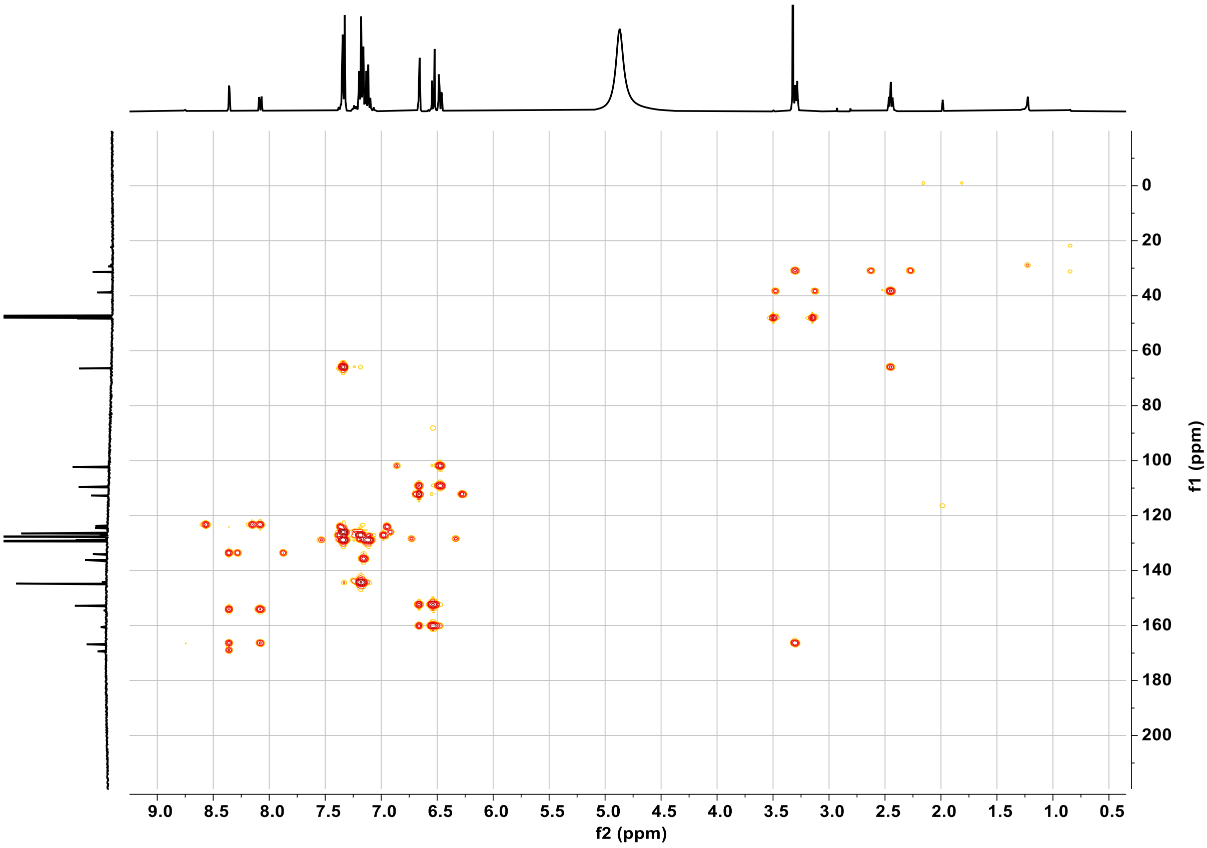

f2 (ppm)
f1 (ppm)

HR-MS:

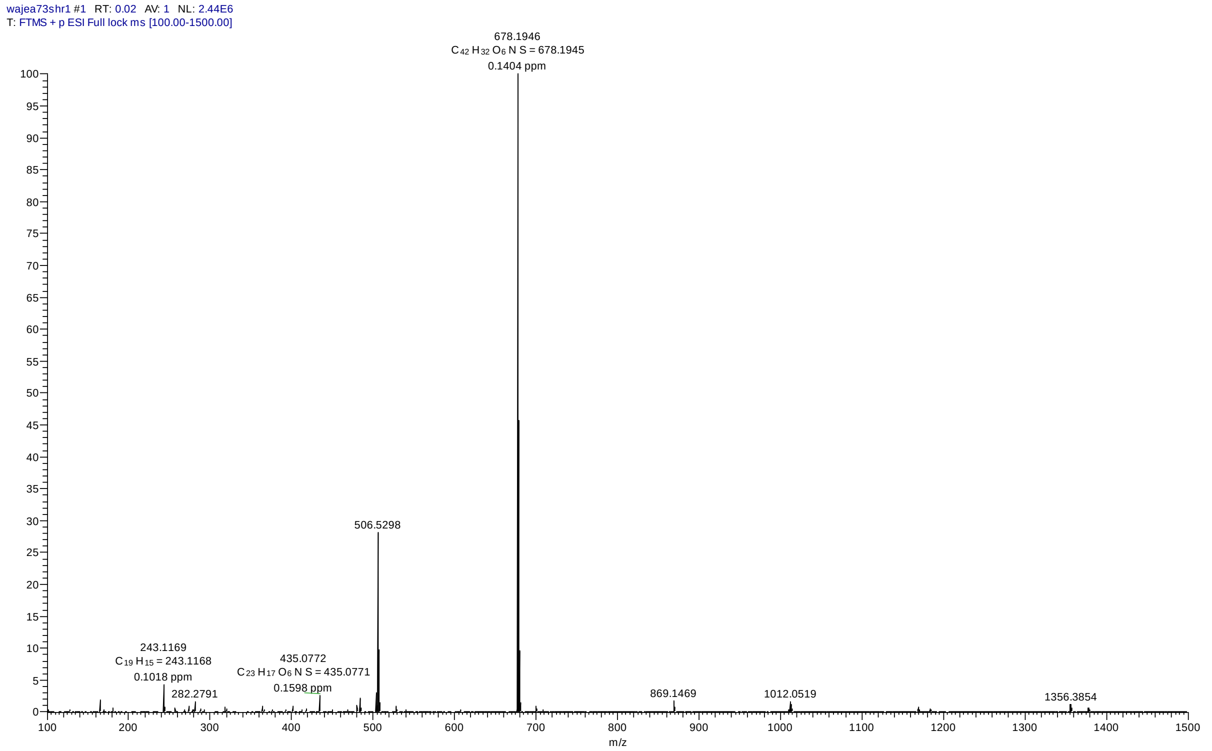

6-(2-tritylthio-ethyl)carbamoyl fluorescein

^{1}H-NMR:

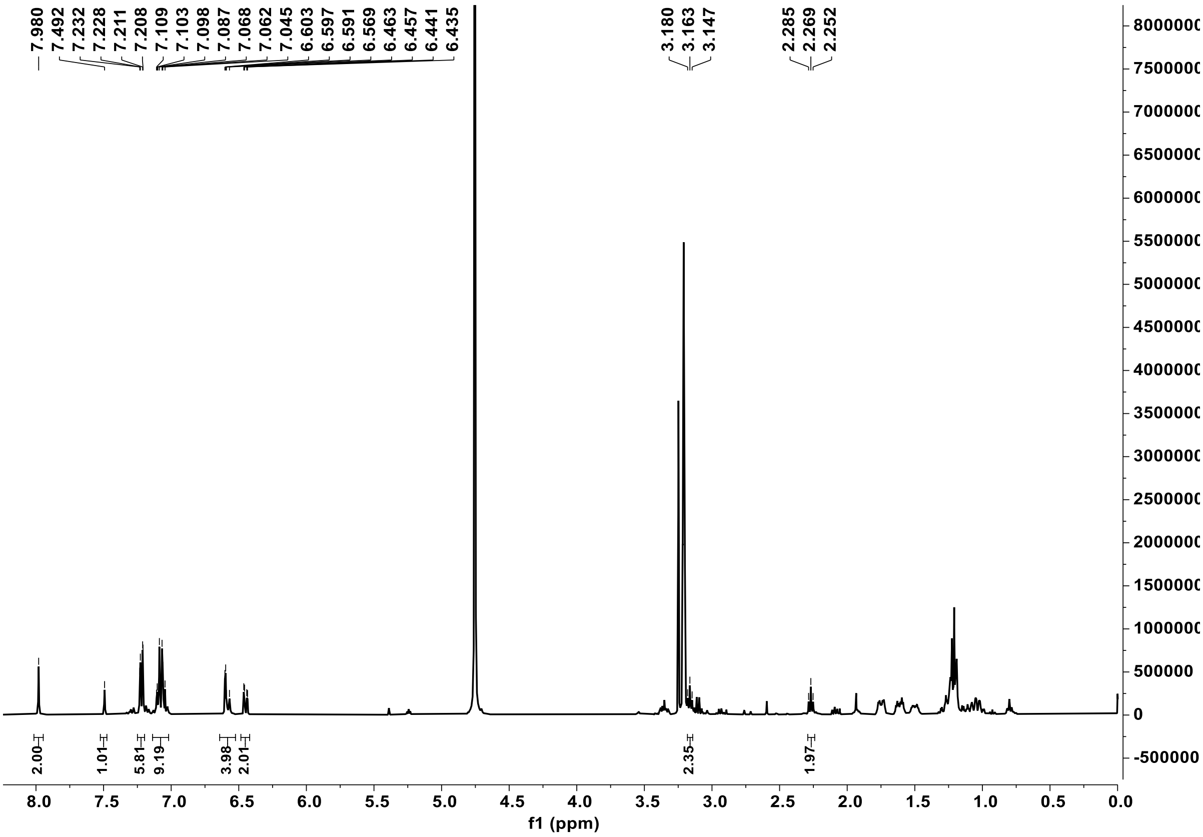

^{13}C-NMR:

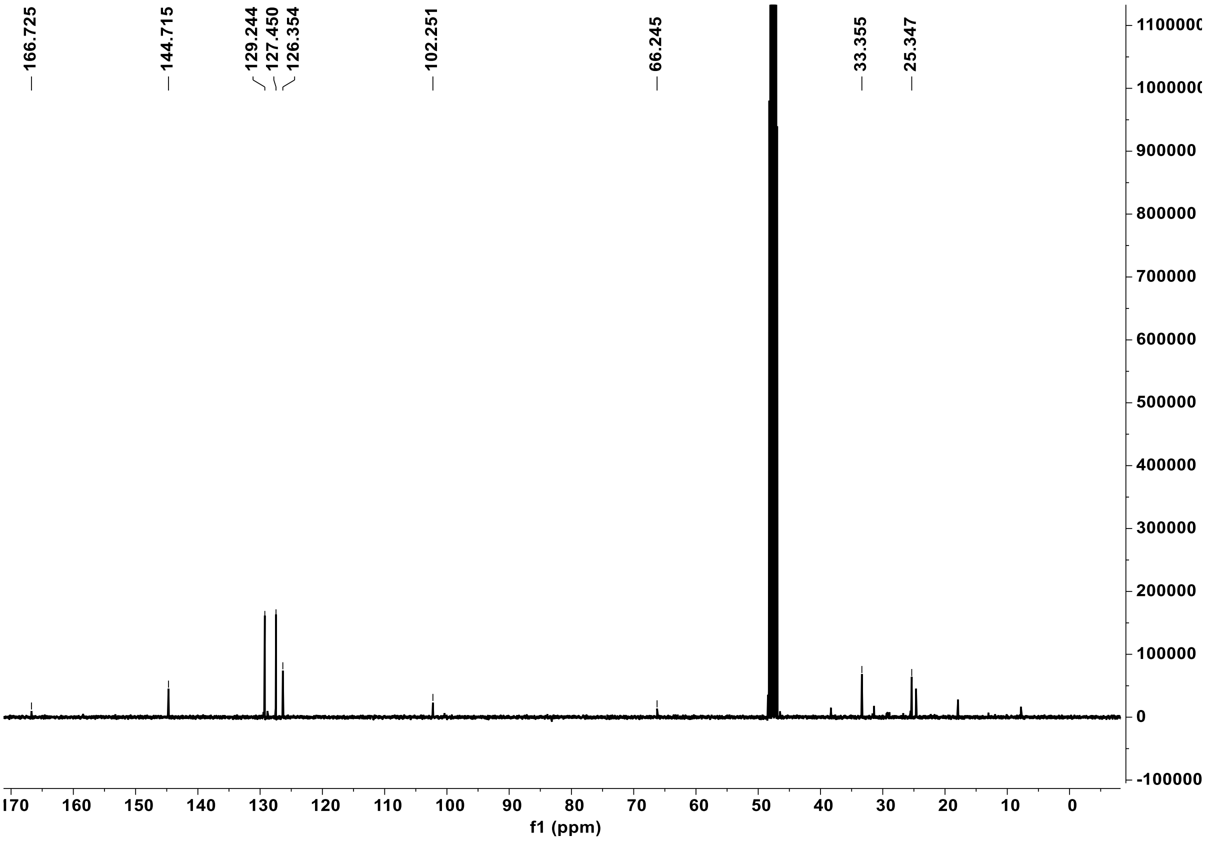

166.725
144.715
129.244
127.450
126.354
102.251
66.245
33.355
25.347
f1 (ppm)

HMBC:

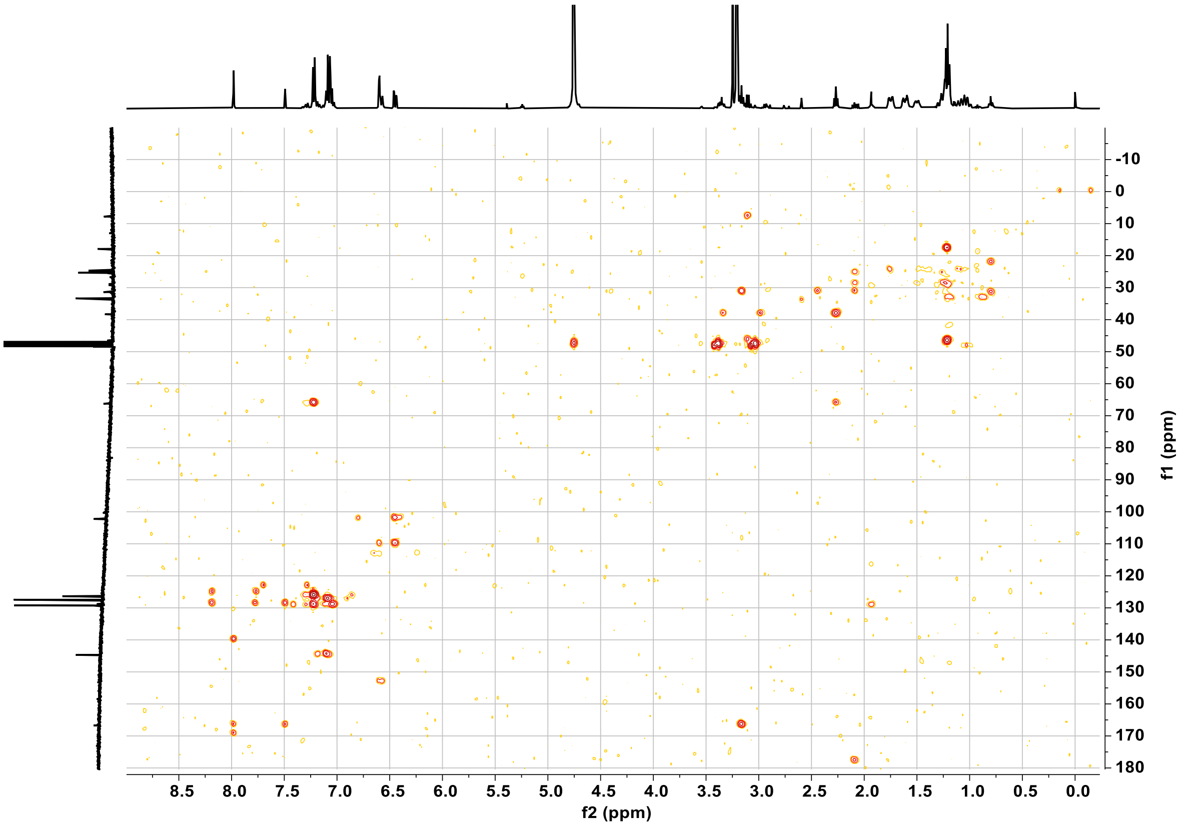

f2 (ppm)
f1 (ppm)

HR-MS:

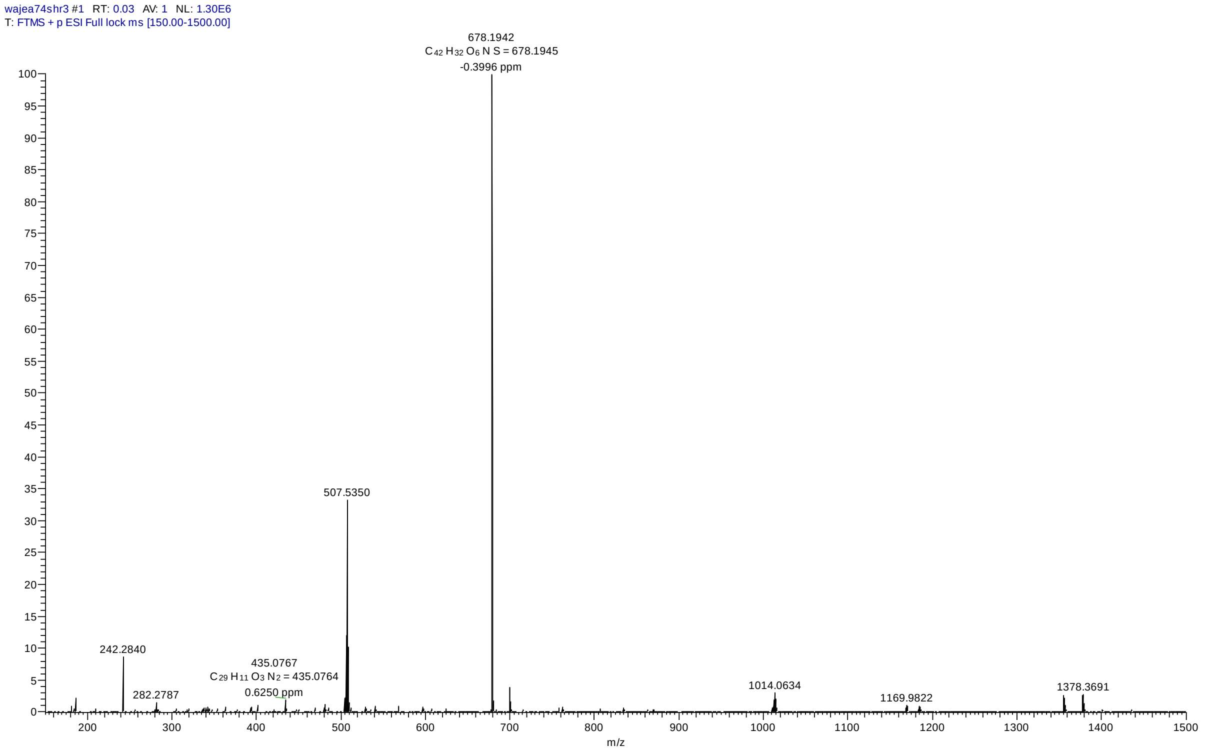

5-(2-mercaptoethyl)-carbamoyl fluorescein (5)

^{1}H-NMR:

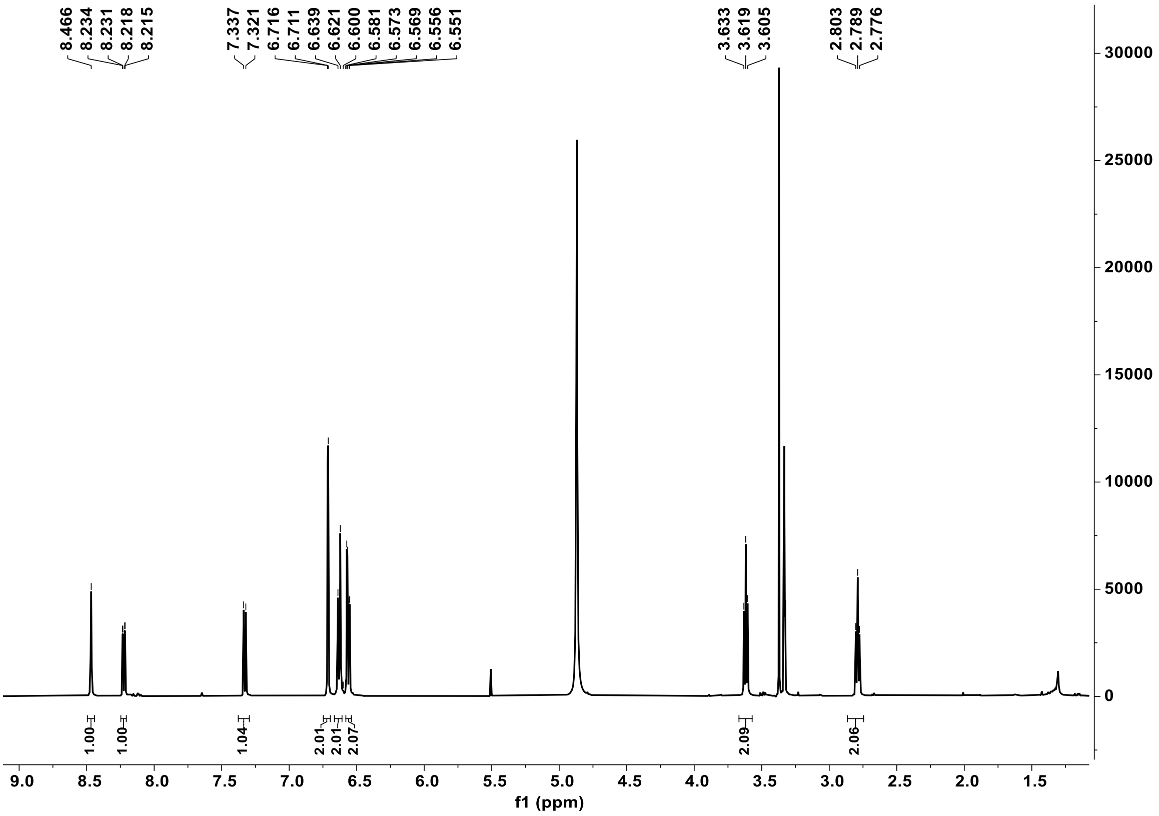

^{13}C-NMR:

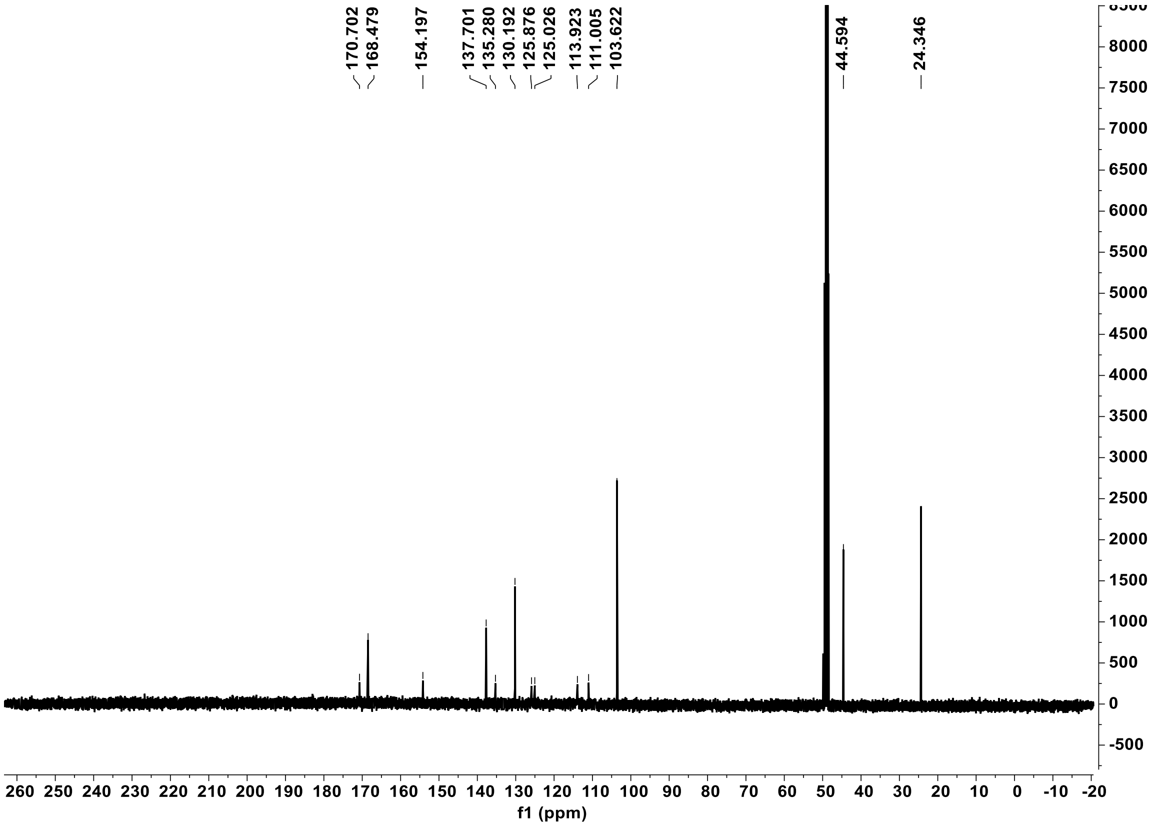
170.702
168.479
154.197
137.701
135.280
130.192
125.876
125.026
113.923
111.005
103.622
44.594
24.346
f1 (ppm)

HMBC:

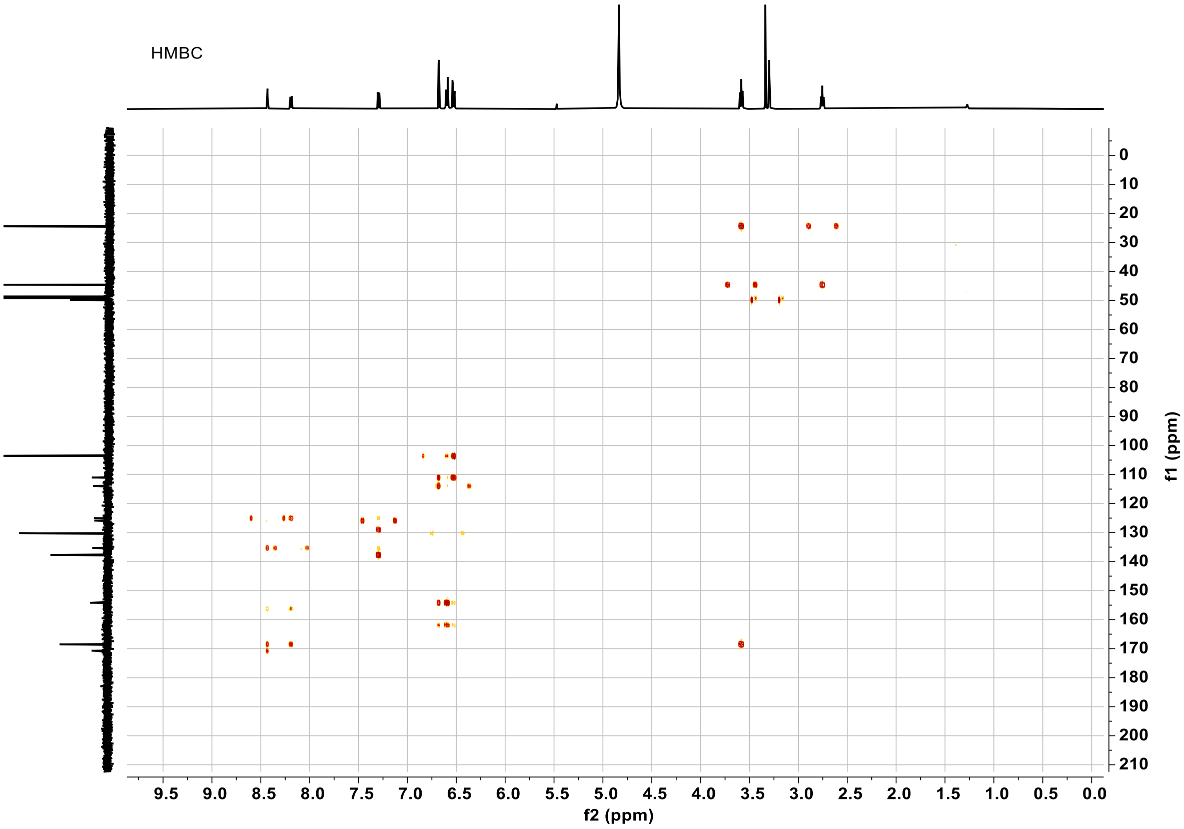
HMBC
f2 (ppm)
f1 (ppm)

HR-MS:

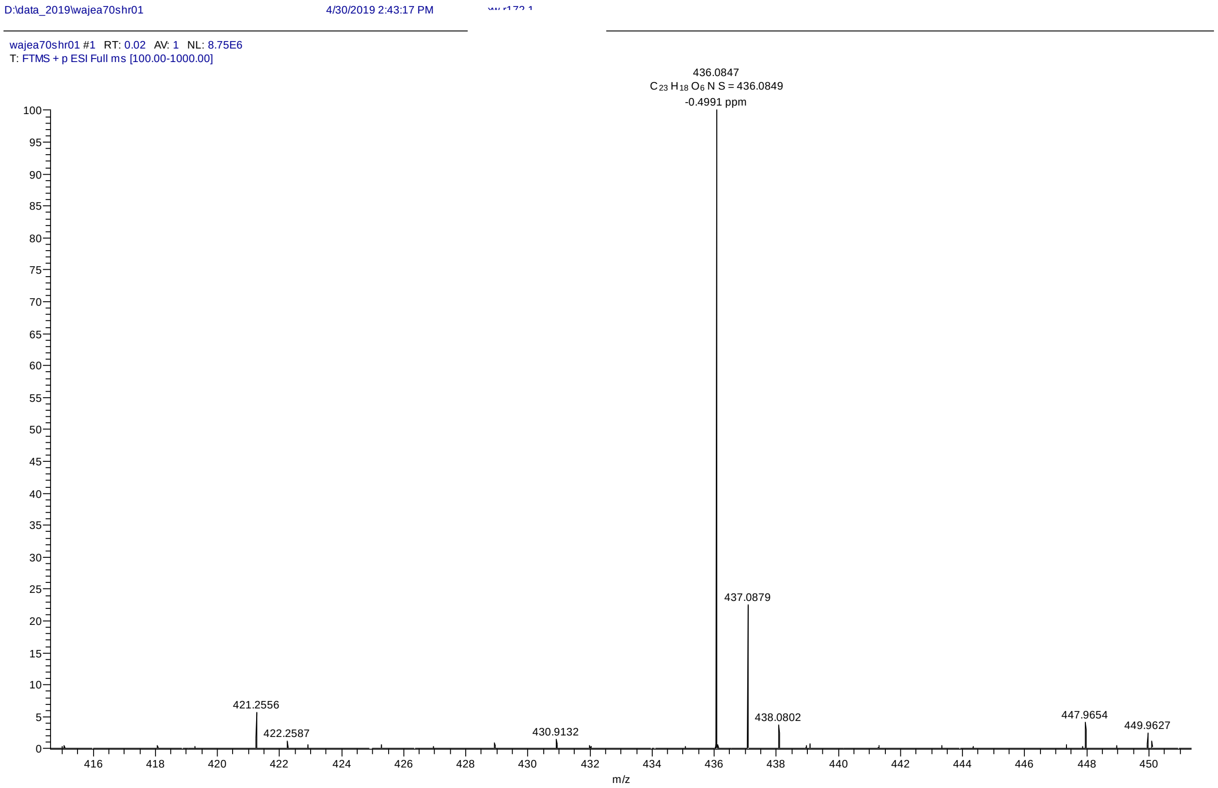

Trehalose-C_2 linker (7)

^{1}H-NMR:

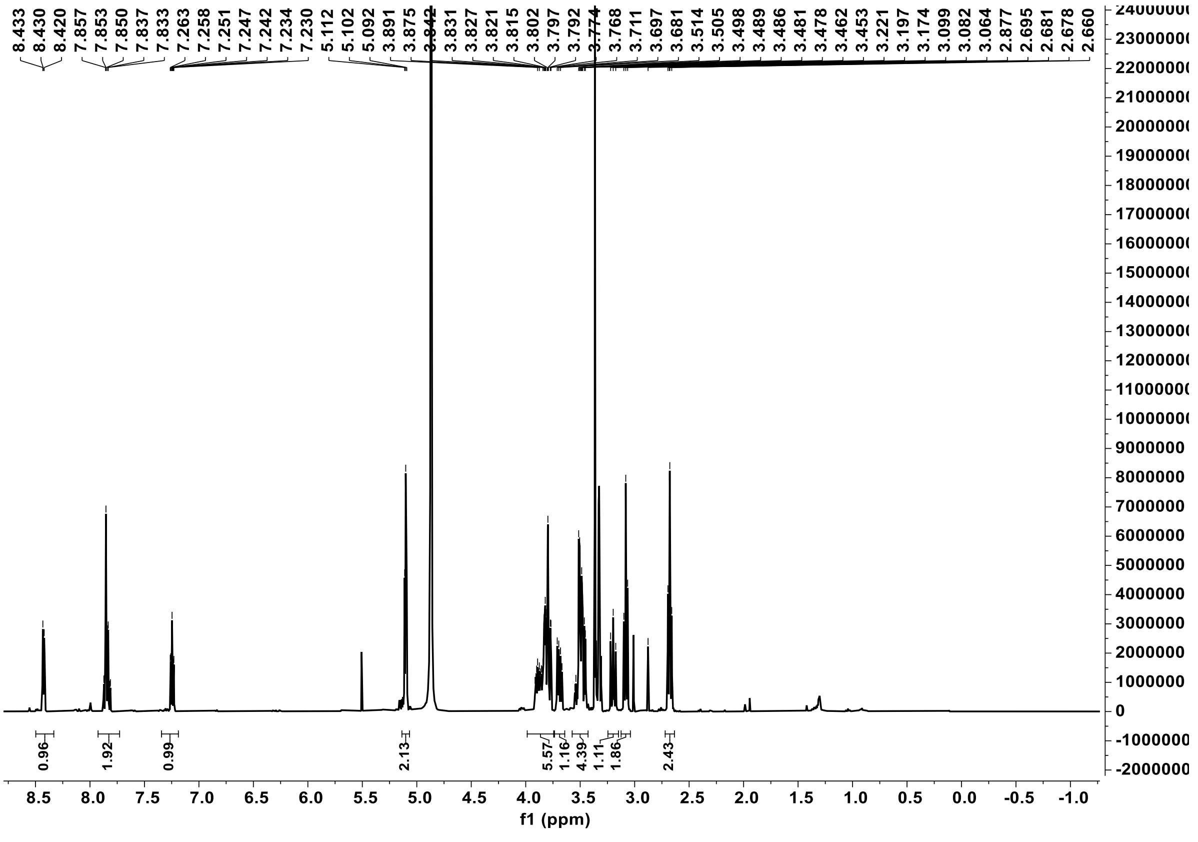

^{13}C-NMR:

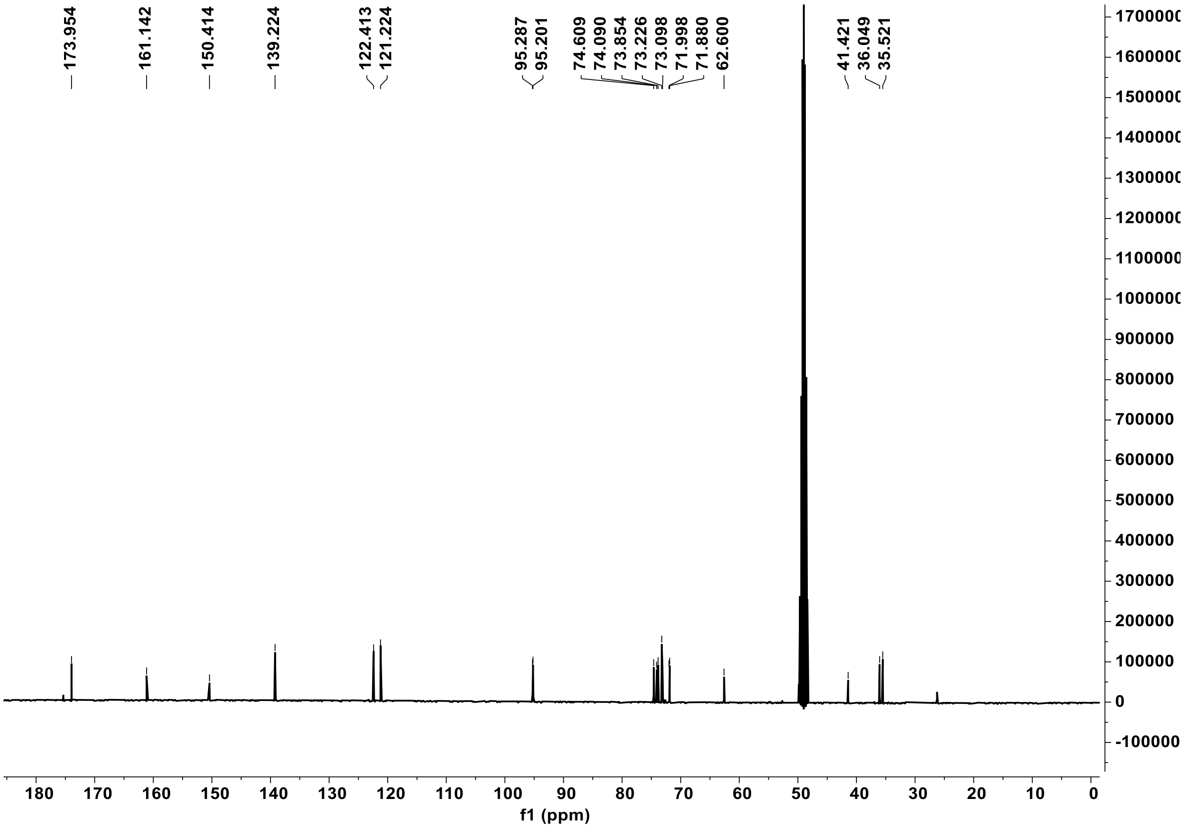
173.954
161.142
150.414
139.224
122.413
121.224
95.287
95.201
74.609
74.090
73.854
73.226
73.098
71.998
71.880
62.600
41.421
36.049
35.521
f1 (ppm)

HR-MS:

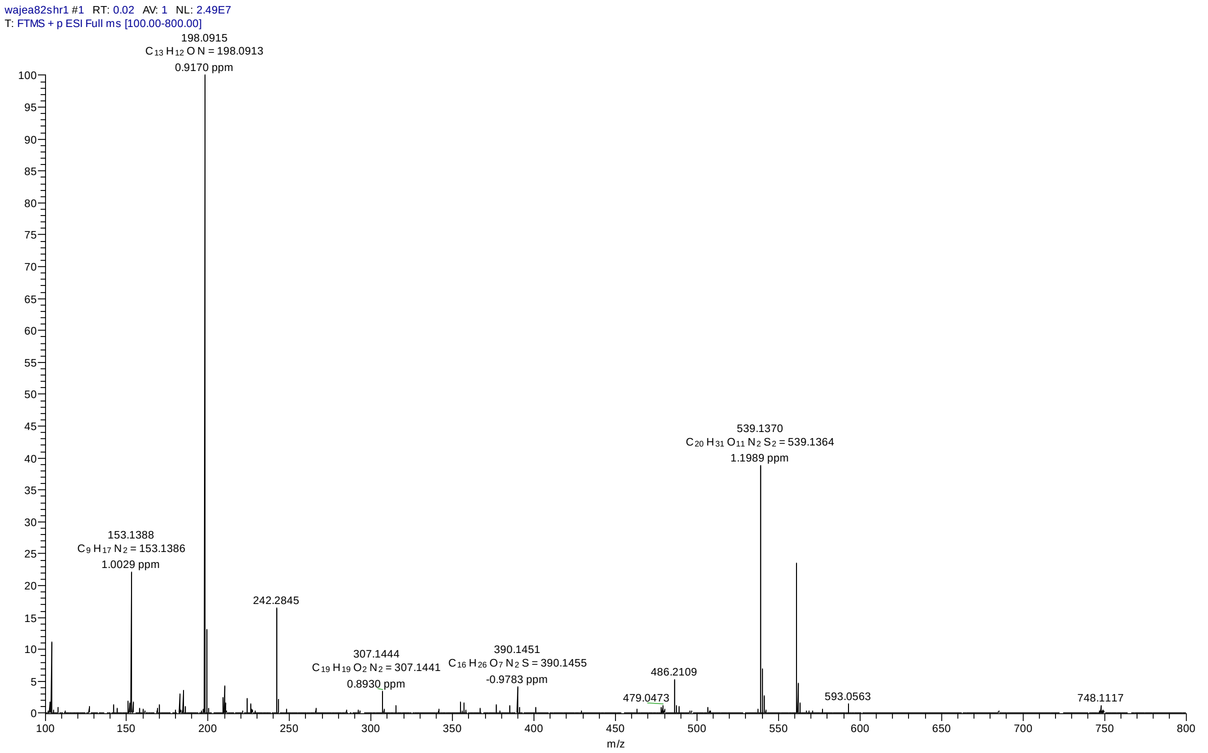
wajea82shr1 #1 RT: 0.02 AV: 1 NL: 2.49E7
T: FTMS + p ESI Full ms [100.00-800.00]
198.0915
C13 H12 O N = 198.0913
0.9170 ppm
153.1388
C9 H17 N2 = 153.1386
1.0029 ppm
242.2845
307.1444
C19 H19 O2 N2 = 307.1441
0.8930 ppm
390.1451
C16 H26 O7 N2 S = 390.1455
-0.9783 ppm
486.2109
479.0473
539.1370
C20 H31 O11 N2 S2 = 539.1364
1.1989 ppm
593.0563
748.1117
m/z

Trehalose-C2-fluorescein (10)

^{1}H-NMR:

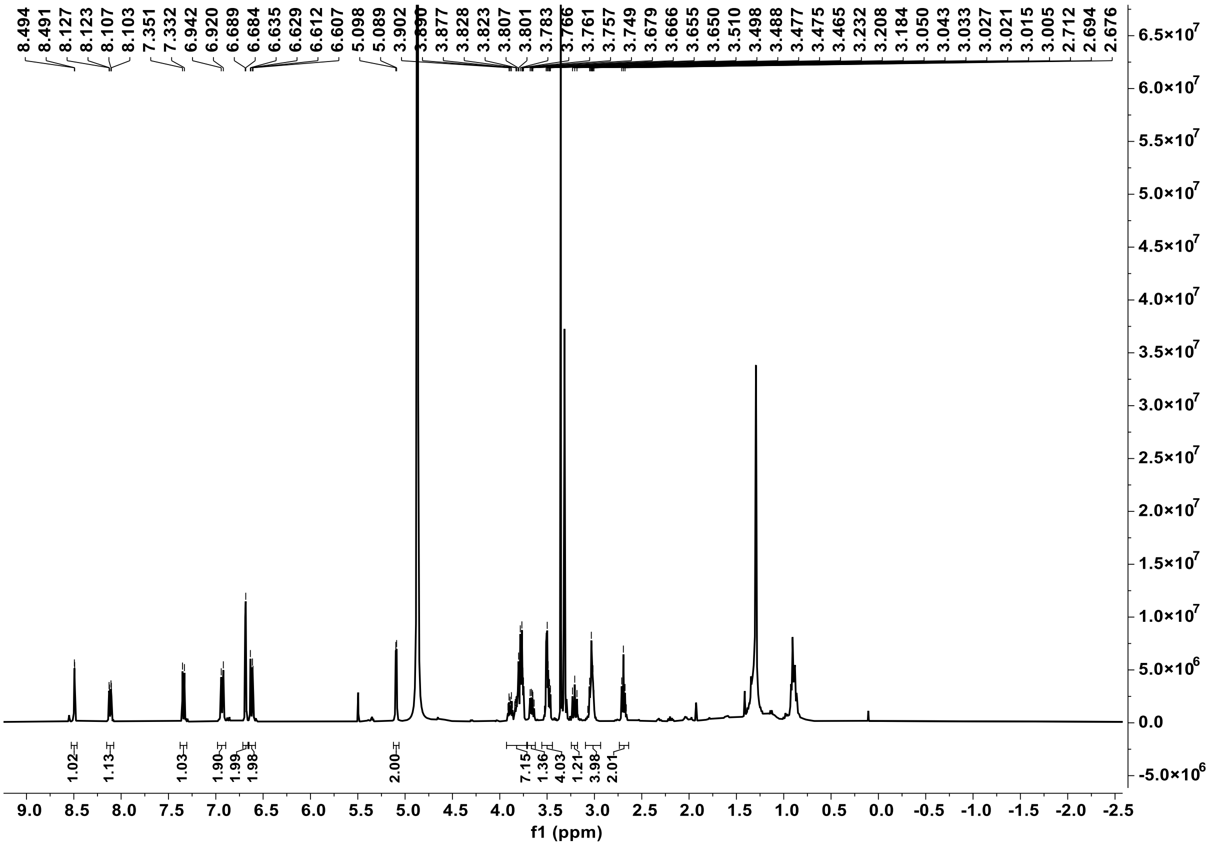

^{13}C-NMR:

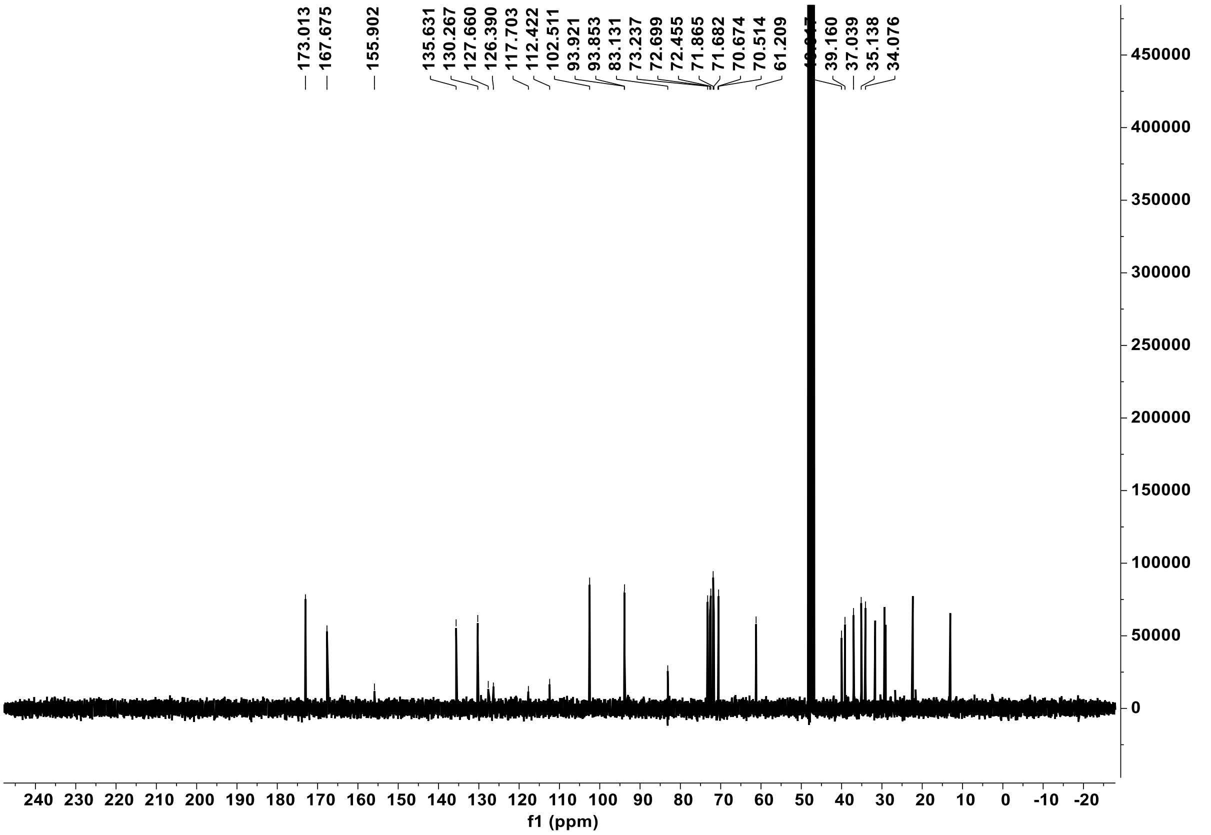

HR-MS:

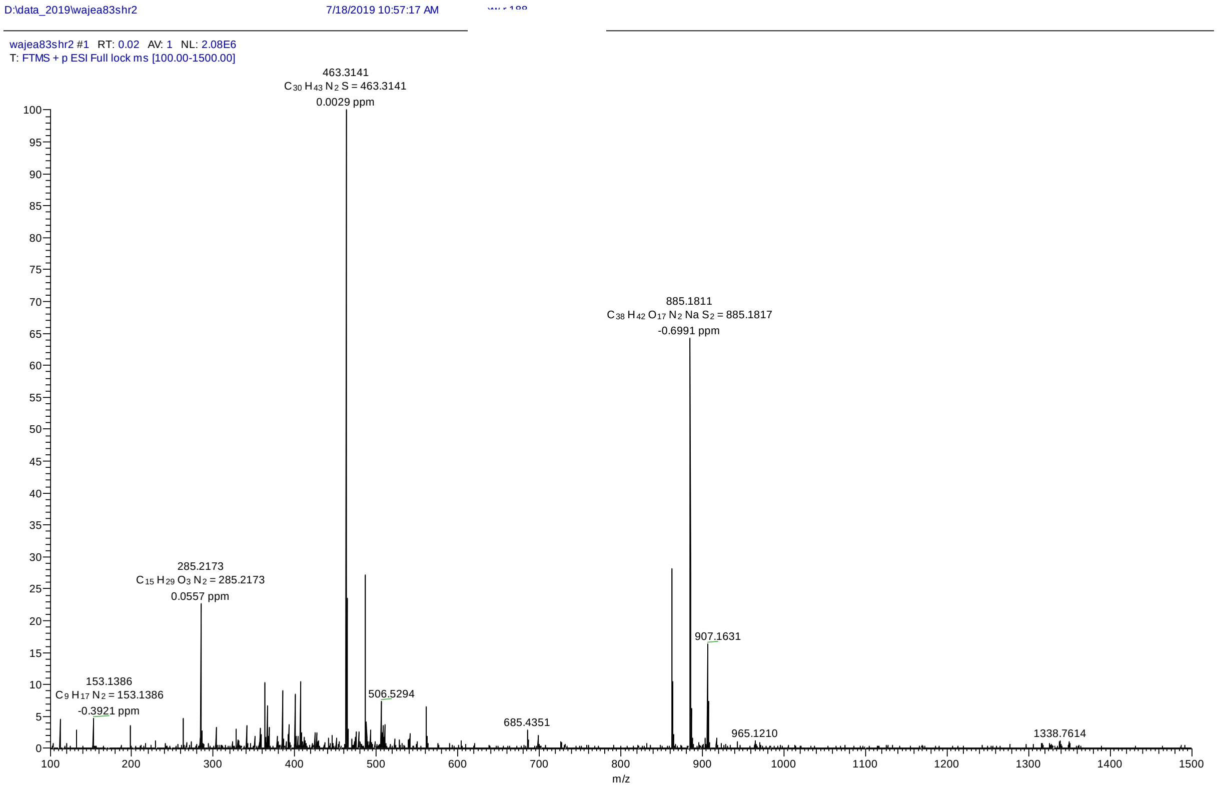

HPLC-UV (254 nm):

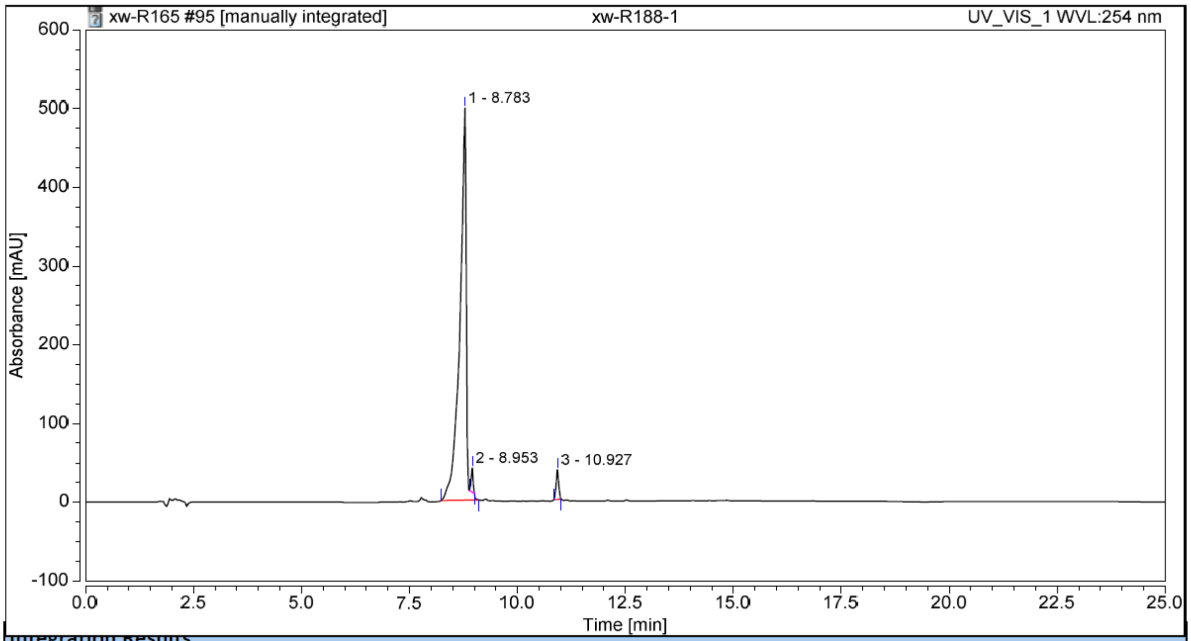

Integration Results

No.	Peak Name	Retention Time min	Area mAU*min	Height mAU	Relative Area %	Relative Height %	Amount n.a.
1		8.783	85.075	498.695	95.36	87.93	n.a.
2		8.953	1.649	30.833	1.85	5.44	n.a.
3		10.927	2.490	37.645	2.79	6.64	n.a.
**Total:**			**89.213**	**567.173**	**100.00**	**100.00**	

Trehalose-PEG4 linker (8)

^{1}H-NMR:

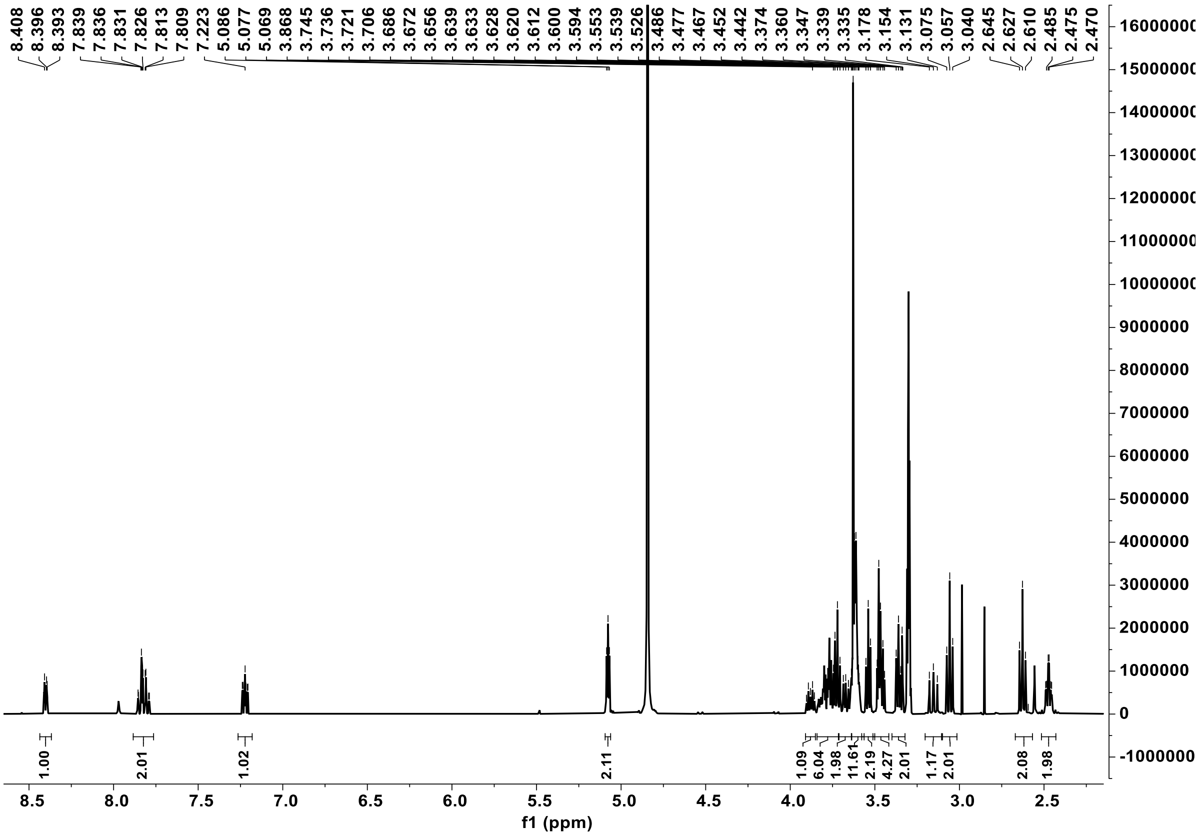

^{13}C-NMR:

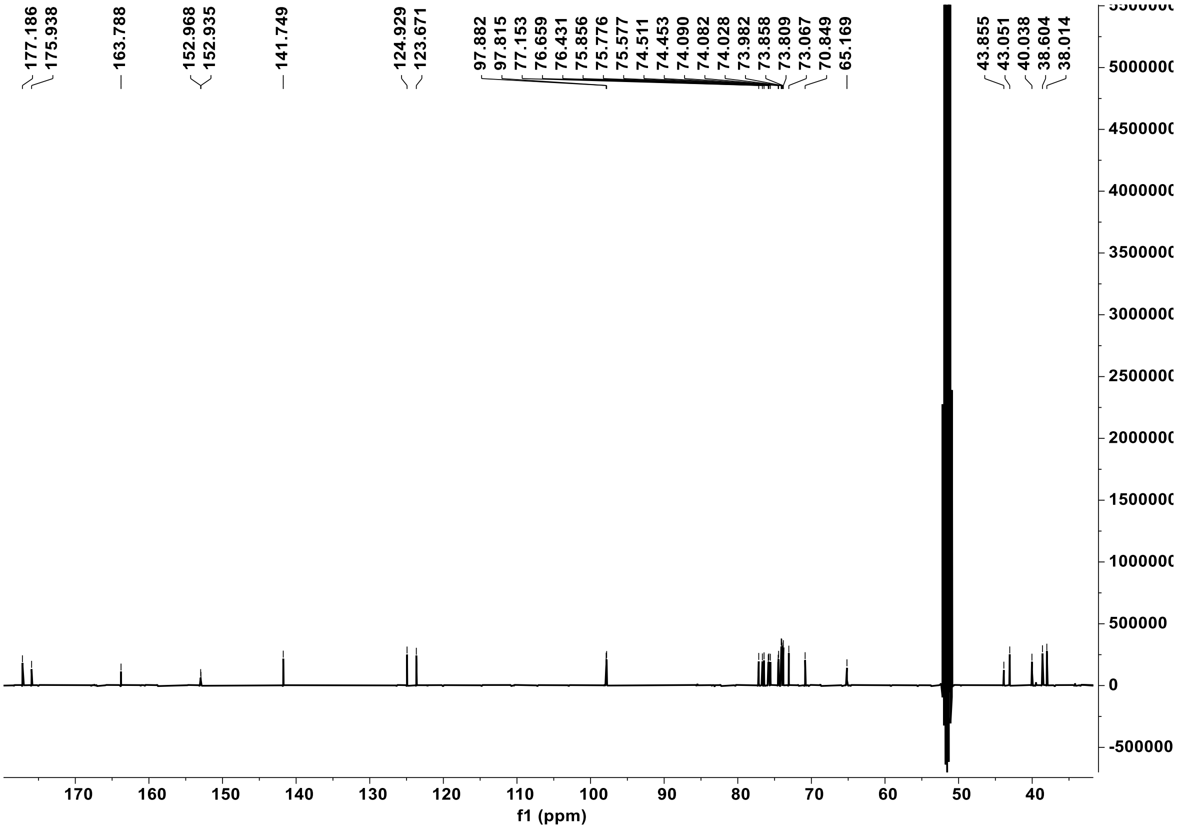

HR-MS:

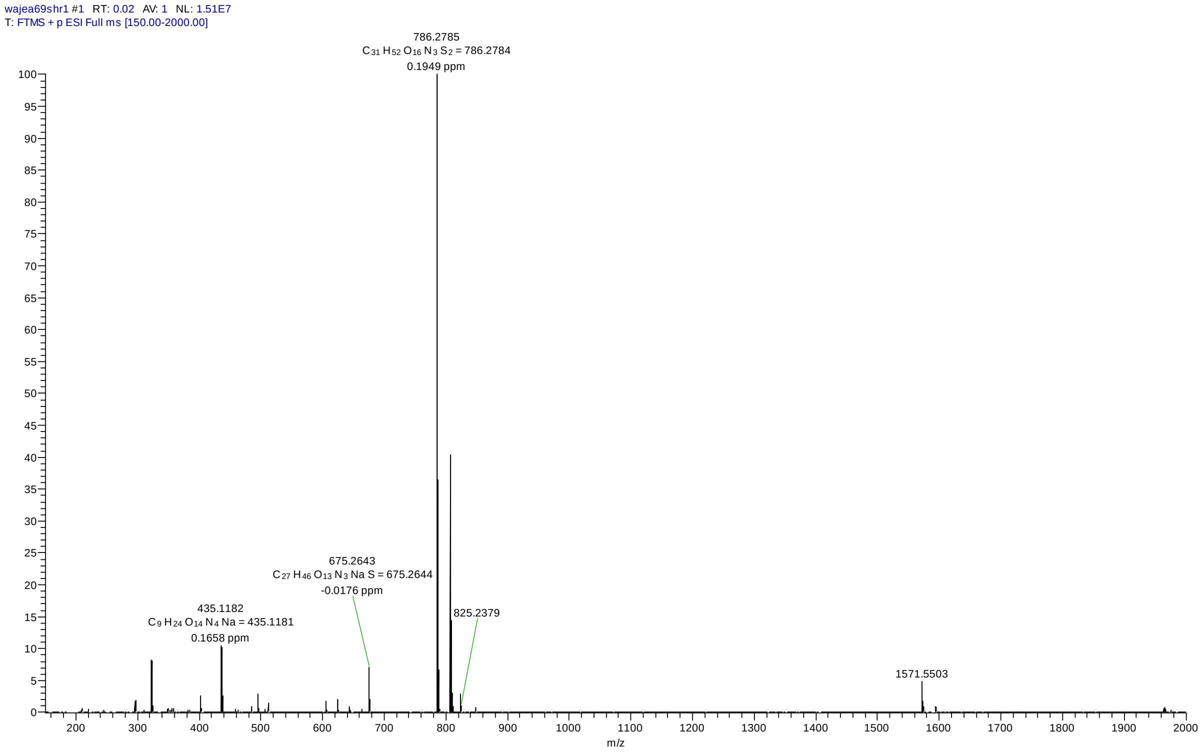

Trehalose-PEG4-fluorescein (11)

^{1}H-NMR:

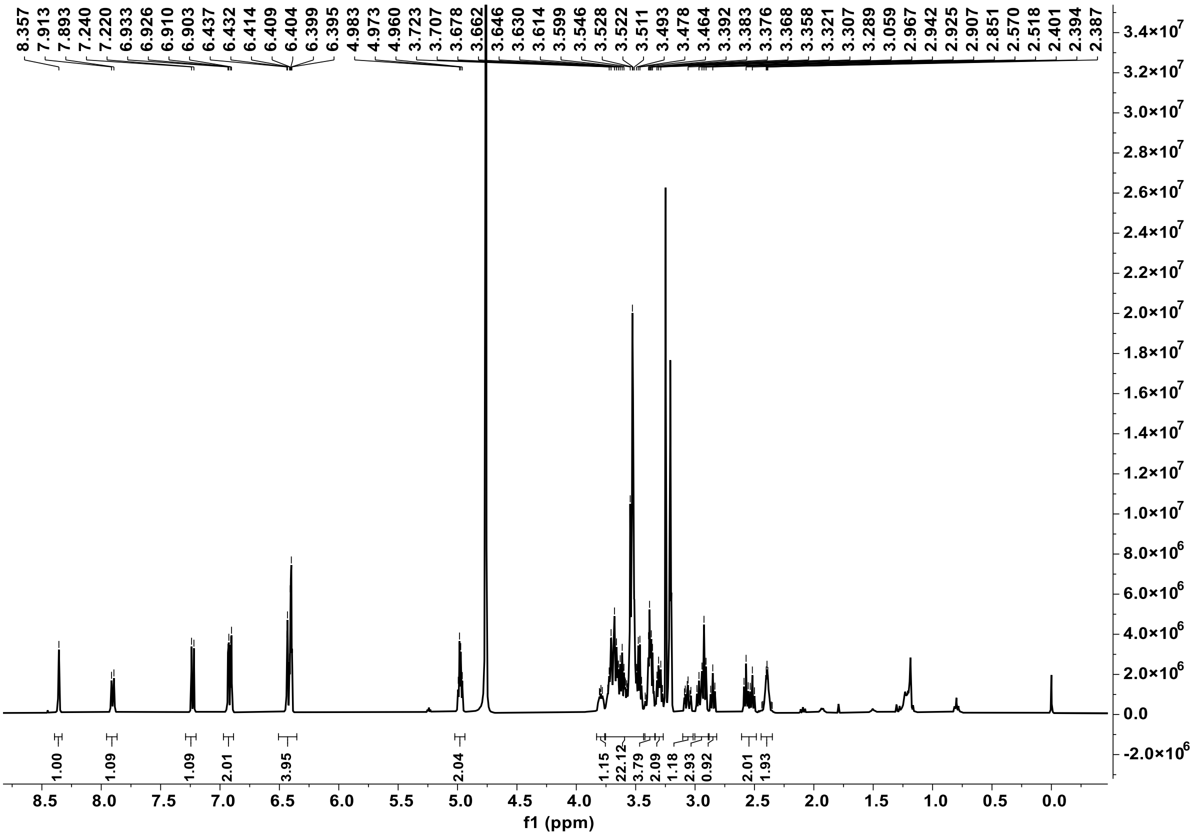

^{13}C-NMR:

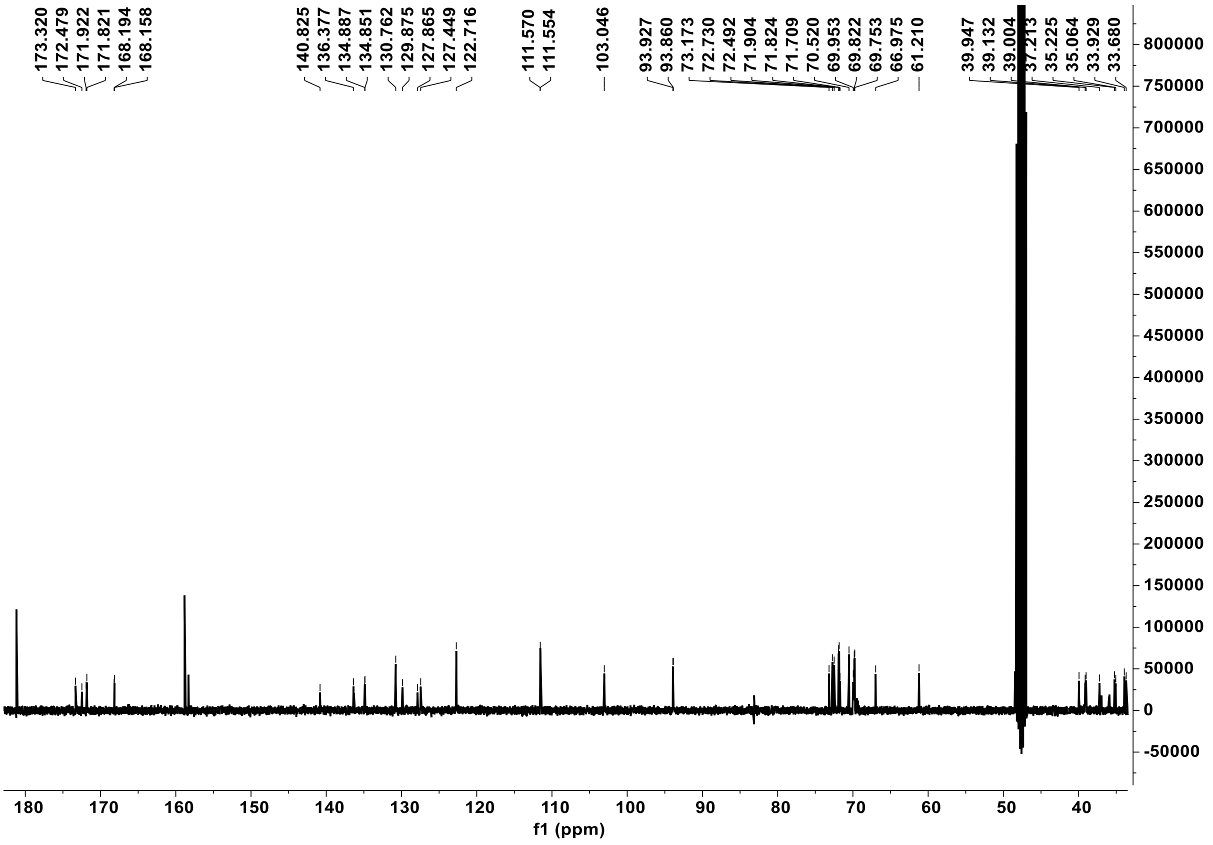
173.320
172.479
171.922
171.821
168.194
168.158
140.825
136.377
134.887
134.851
130.762
129.875
127.865
127.449
122.716
111.570
111.554
103.046
93.927
93.860
73.173
72.730
72.492
71.904
71.824
71.709
70.520
69.953
69.822
69.753
66.975
61.210
39.947
39.132
39.004
37.213
35.225
35.064
33.929
33.680
f1 (ppm)

HR-MS:

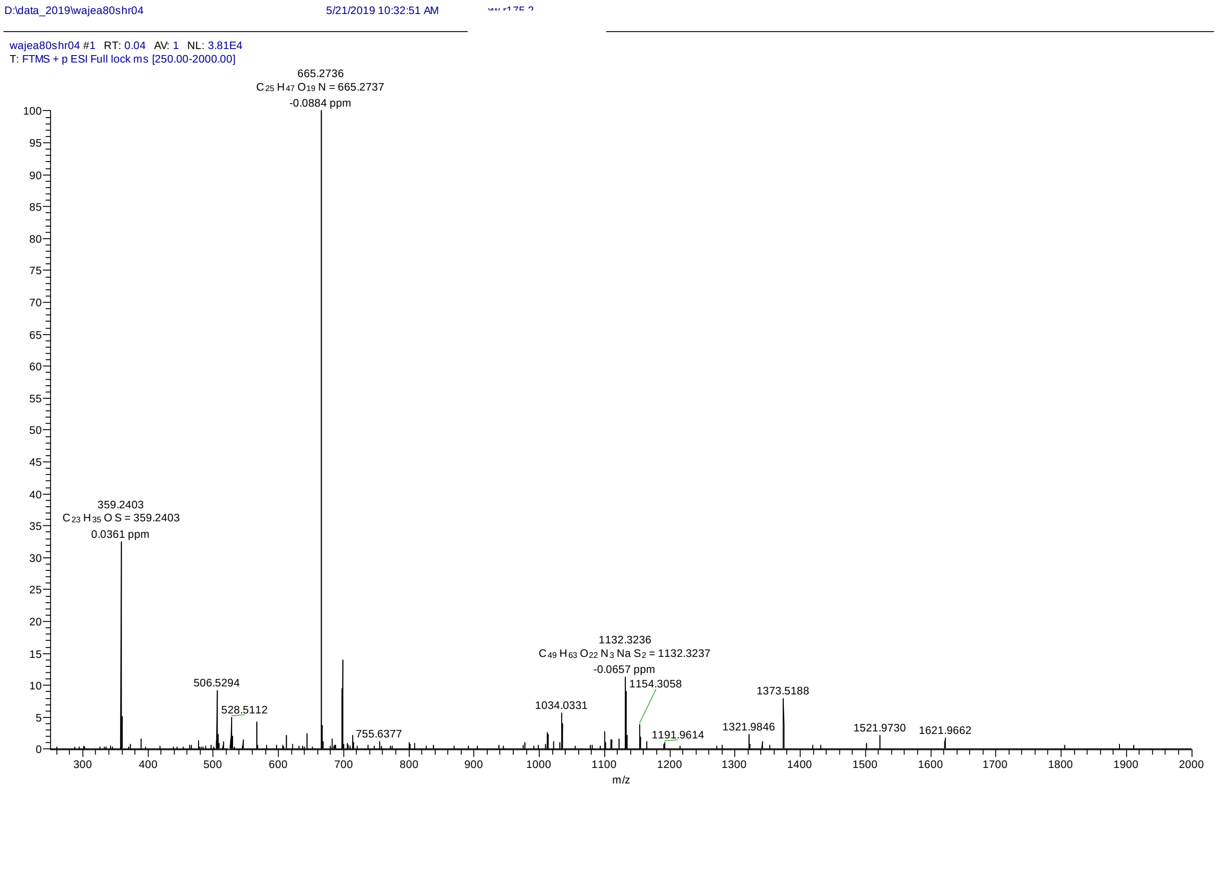
D:\data_2019\wajea80shr04
5/21/2019 10:32:51 AM
wajea80shr04 #1 RT: 0.04 AV: 1 NL: 3.81E4
T: FTMS + p ESI Full lock ms [250.00-2000.00]
665.2736
C25 H47 O19 N = 665.2737
-0.0884 ppm
359.2403
C23 H35 O S = 359.2403
0.0361 ppm
506.5294
528.5112
755.6377
1132.3236
C49 H63 O22 N3 Na S2 = 1132.3237
-0.0657 ppm
1154.3058
1034.0331
1191.9614
1321.9846
1373.5188
1521.9730
1621.9662
m/z

HPLC-UV (254 nm):

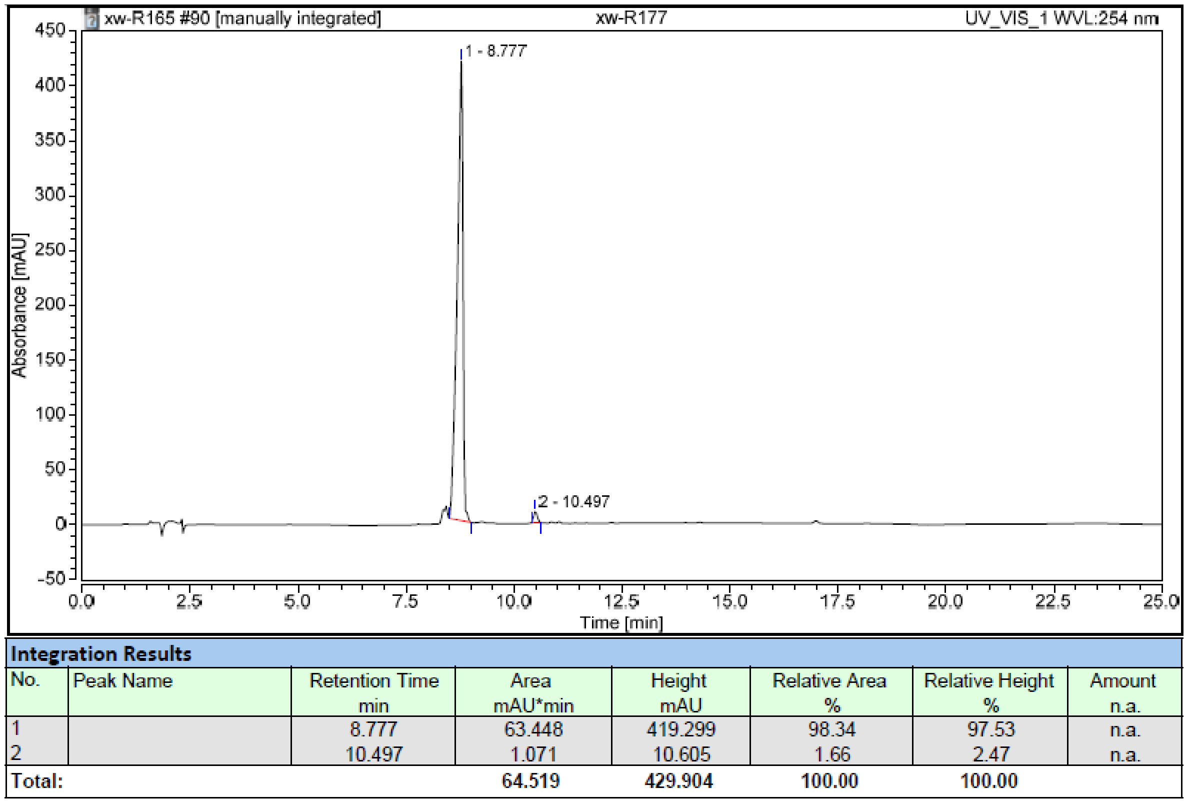

Integration Results							
No.	Peak Name	Retention Time<br>min	Area<br>mAU*min	Height<br>mAU	Relative Area<br>%	Relative Height<br>%	Amount<br>n.a.
1		8.777	63.448	419.299	98.34	97.53	n.a.
2		10.497	1.071	10.605	1.66	2.47	n.a.
Total:			64.519	429.904	100.00	100.00	

Trehalose-PEG$_{12}$ linker (9)

^{1}H-NMR:

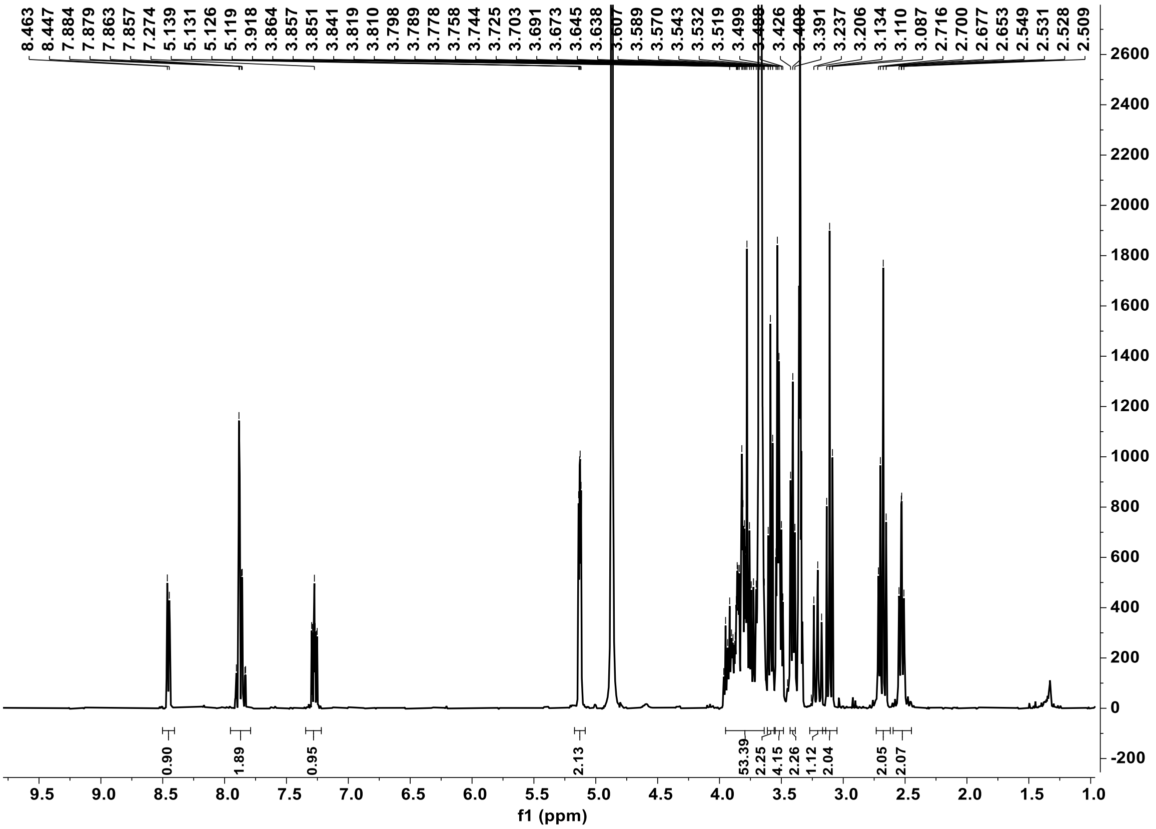

^{13}C-NMR:

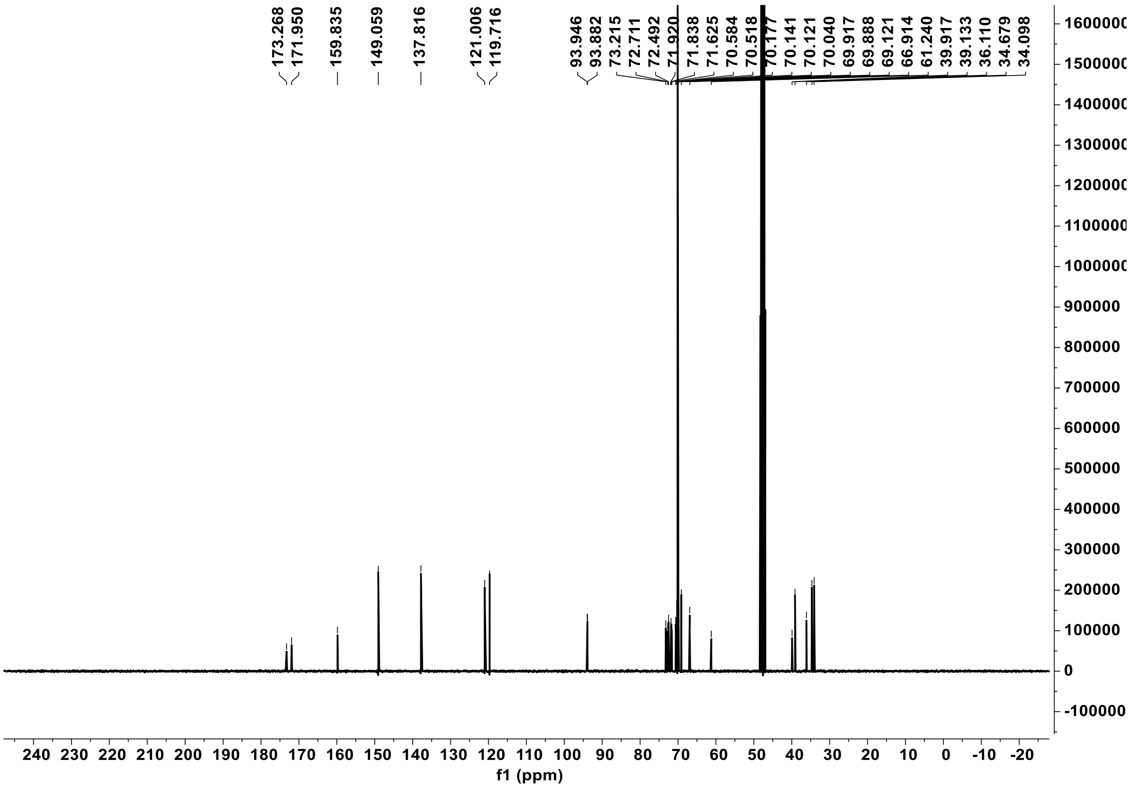

Trehalose-PEG12 fluorescein (12)

^{1}H-NMR:

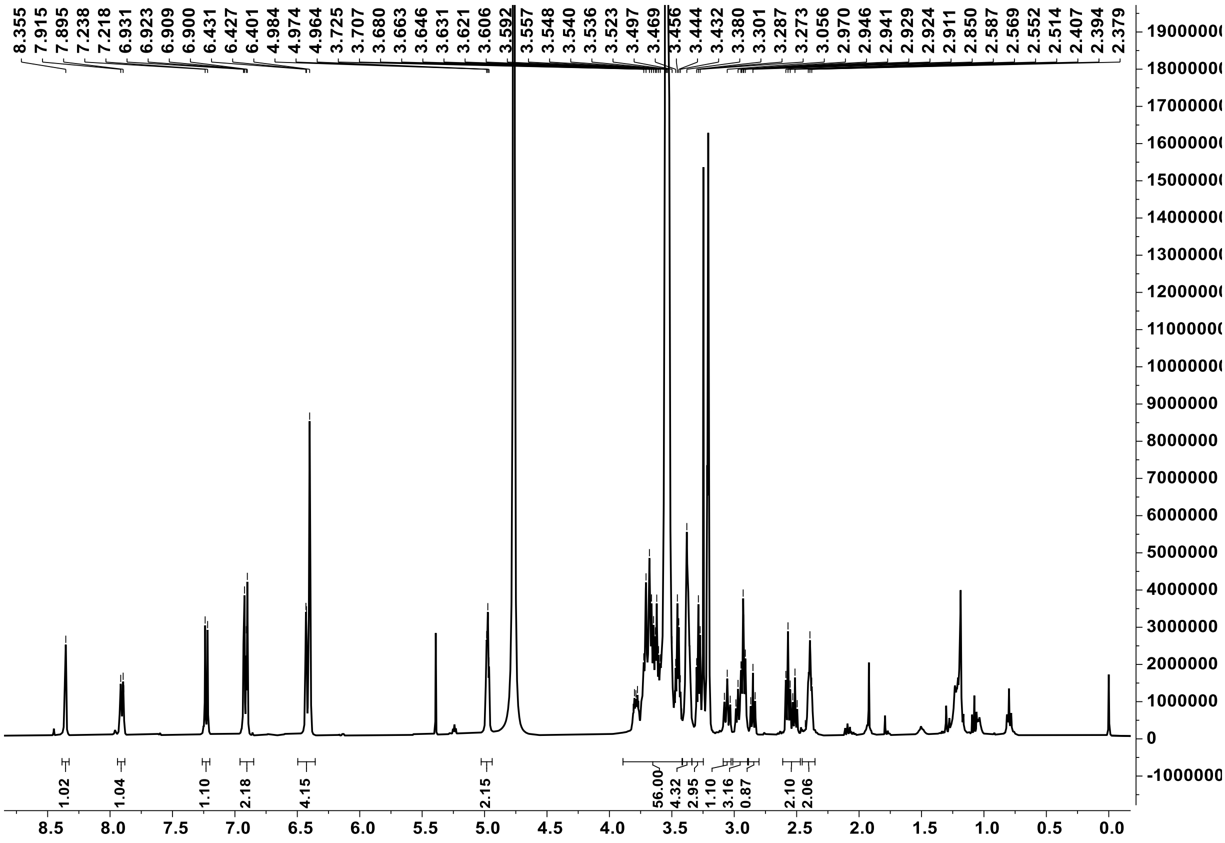

^{13}C-NMR:

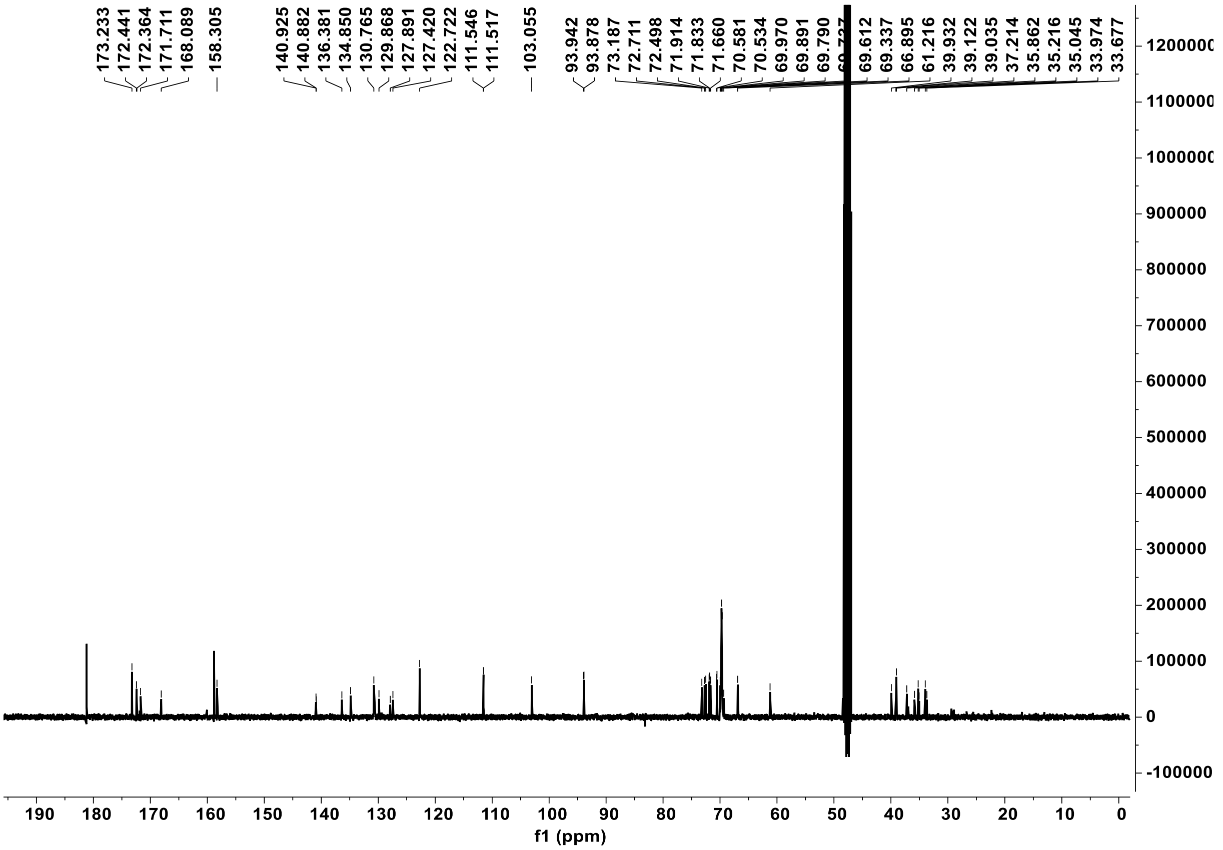
173.233
172.441
172.364
171.711
168.089
158.305
140.925
140.882
136.381
134.850
130.765
129.868
127.891
127.420
122.722
111.546
111.517
103.055
93.942
93.878
73.187
72.711
72.498
71.914
71.833
71.660
70.581
70.534
69.970
69.891
69.790
69.612
69.337
66.895
61.216
39.932
39.122
39.035
37.214
35.862
35.216
35.045
33.974
33.677
f1 (ppm)

HR-MS:

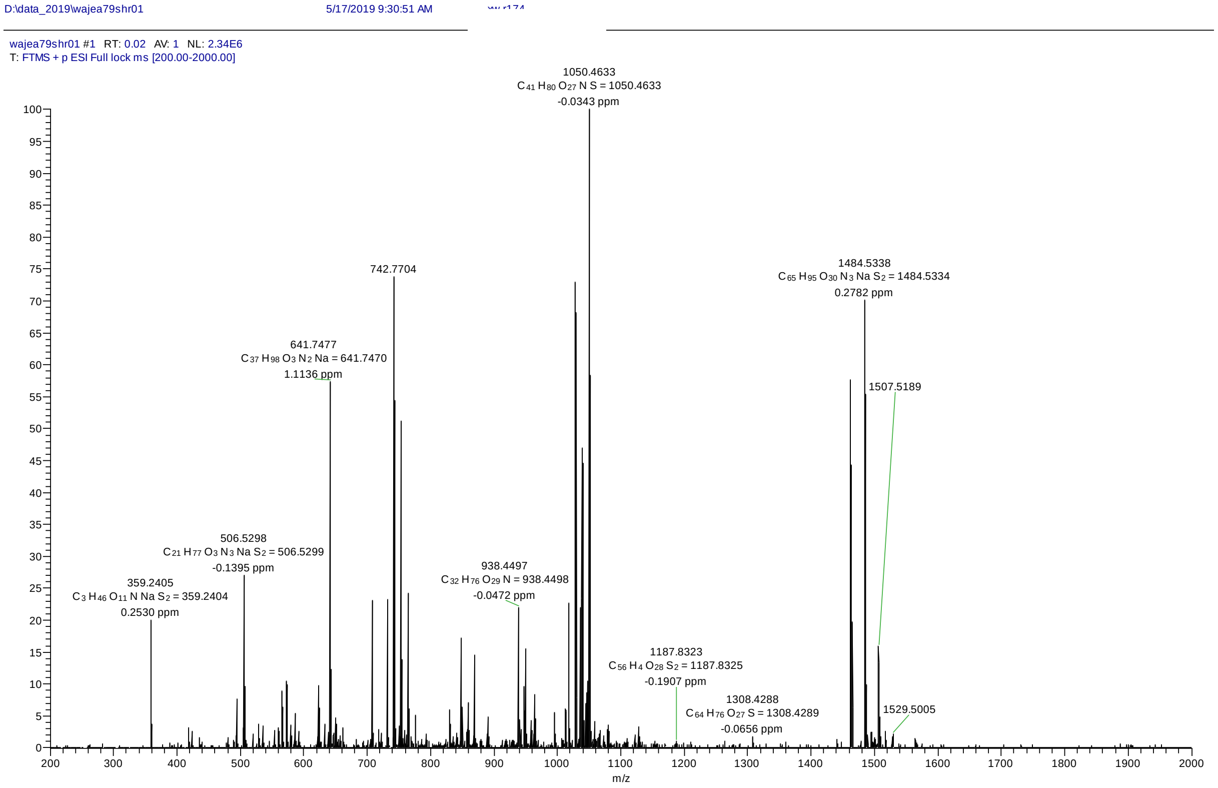
D:\data_2019\wajea79shr01
5/17/2019 9:30:51 AM
wajea79shr01 #1 RT: 0.02 AV: 1 NL: 2.34E6
T: FTMS + p ESI Full lock ms [200.00-2000.00]
1050.4633
C41 H80 O27 N S = 1050.4633
-0.0343 ppm
742.7704
641.7477
C37 H98 O3 N2 Na = 641.7470
1.1136 ppm
1484.5338
C65 H95 O30 N3 Na S2 = 1484.5334
0.2782 ppm
1507.5189
506.5298
C21 H77 O3 N3 Na S2 = 506.5299
-0.1395 ppm
359.2405
C3 H46 O11 N Na S2 = 359.2404
0.2530 ppm
938.4497
C32 H76 O29 N = 938.4498
-0.0472 ppm
1187.8323
C56 H4 O28 S2 = 1187.8325
-0.1907 ppm
1308.4288
C64 H76 O27 S = 1308.4289
-0.0656 ppm
1529.5005
m/z

HPLC-UV (254 nm):

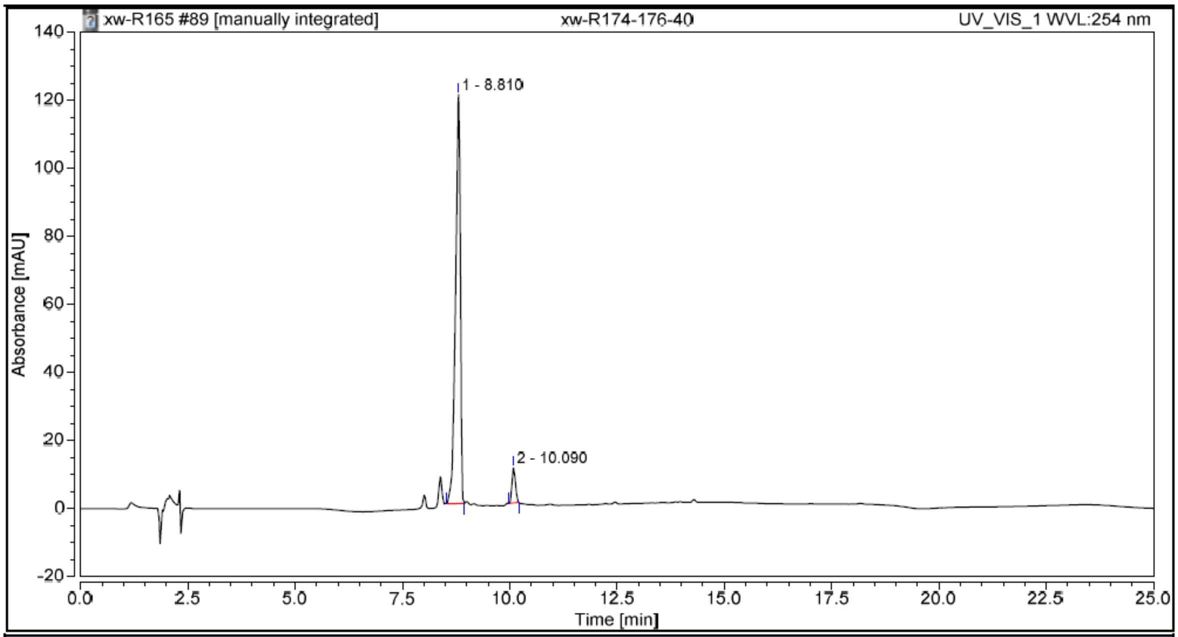

Integration Results

No.	Peak Name	Retention Time min	Area mAU*min	Height mAU	Relative Area %	Relative Height %	Amount n.a.
1		8.810	15.533	120.224	94.26	92.11	n.a.
2		10.090	0.946	10.299	5.74	7.89	n.a.
**Total:**			**16.478**	**130.523**	**100.00**	**100.00**	

Trehalose-PEG$_4$ mertansine (2)

^{1}H-NMR:

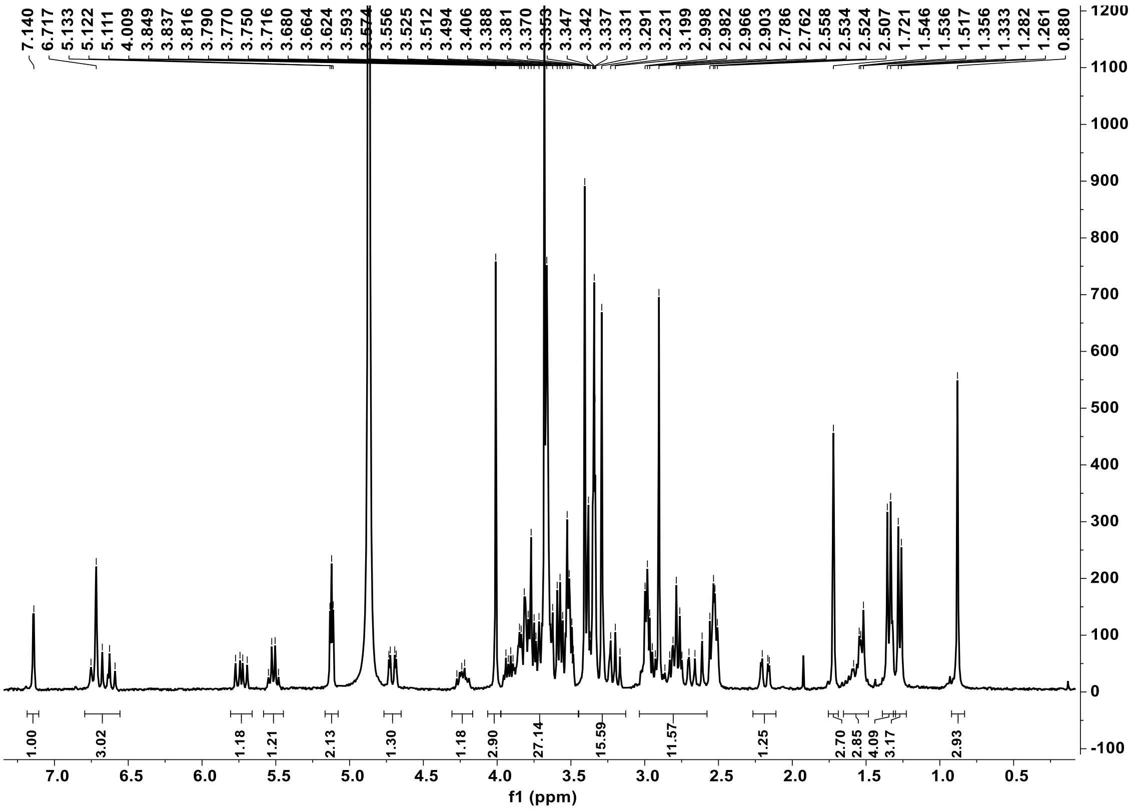

^{13}C-NMR:

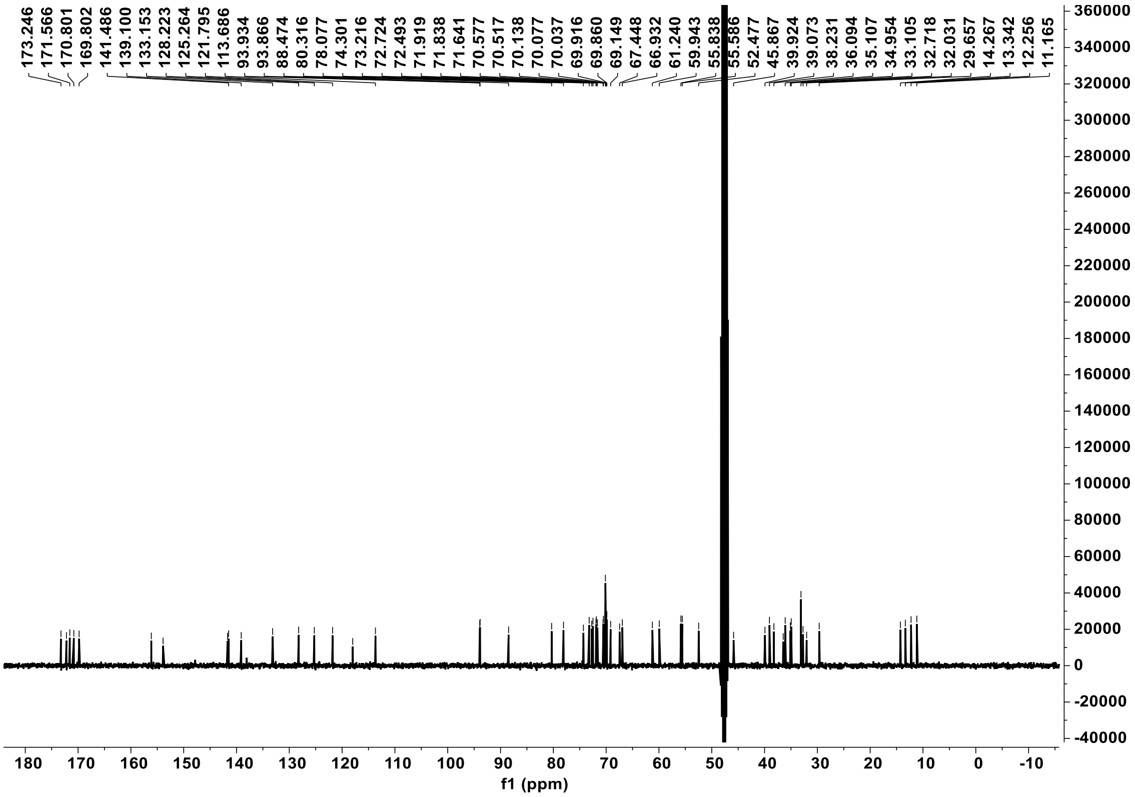

^{1}H- ^{1}H-COSY:

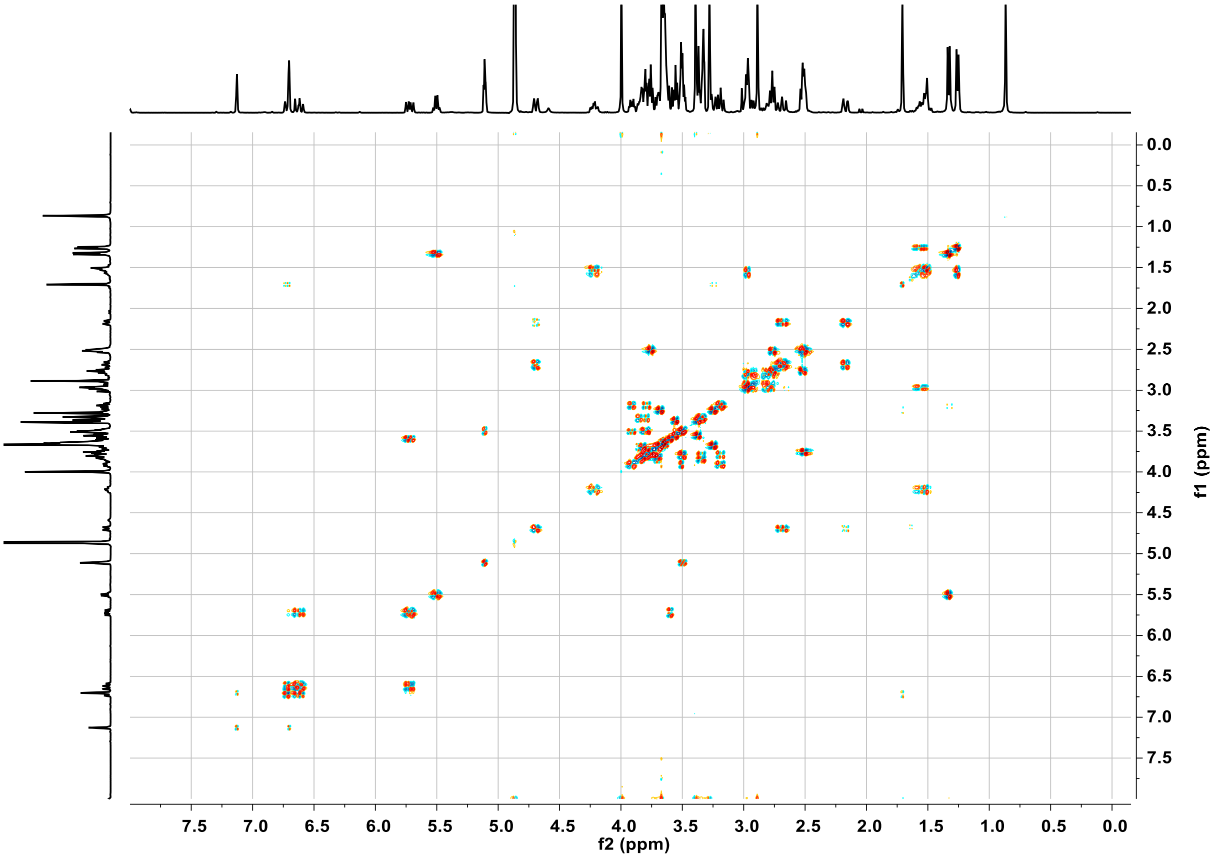

^{1}H- ^{13}C-HSQC:

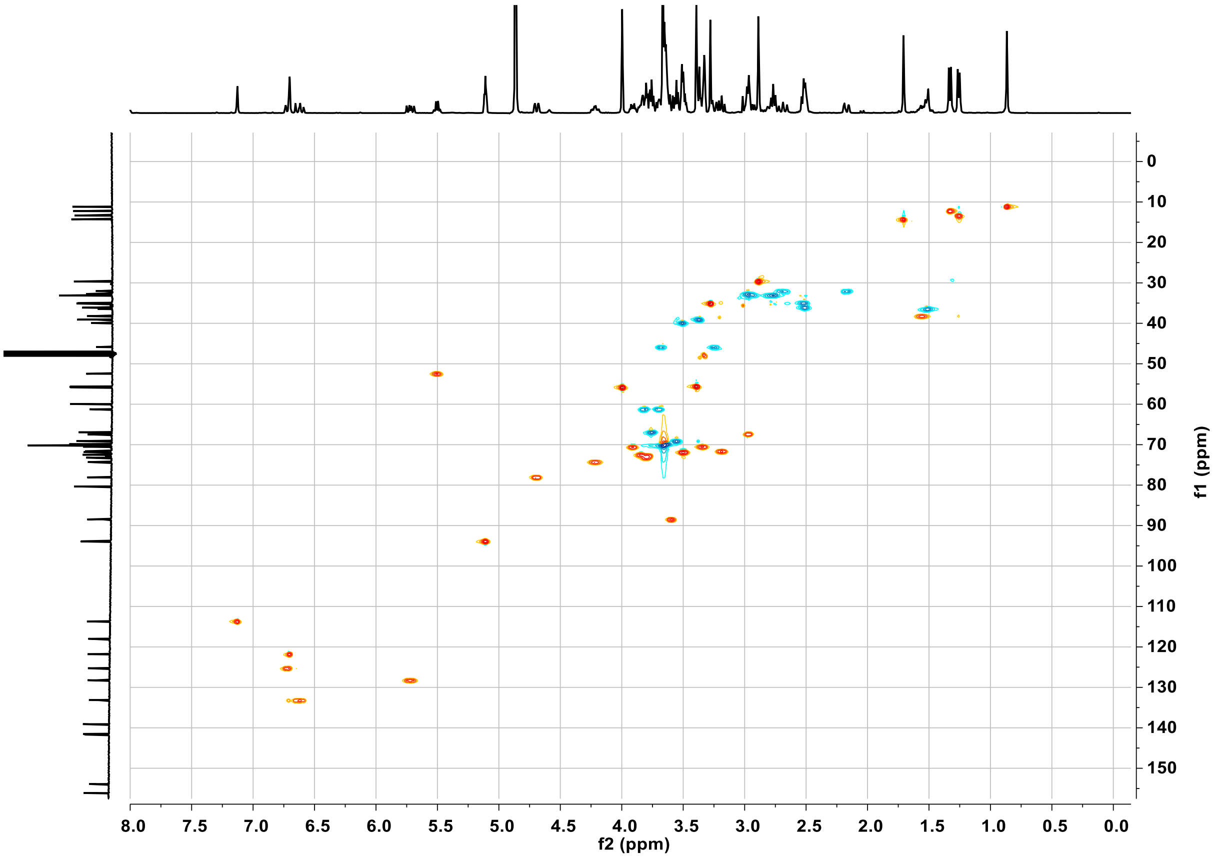

HR-MS:

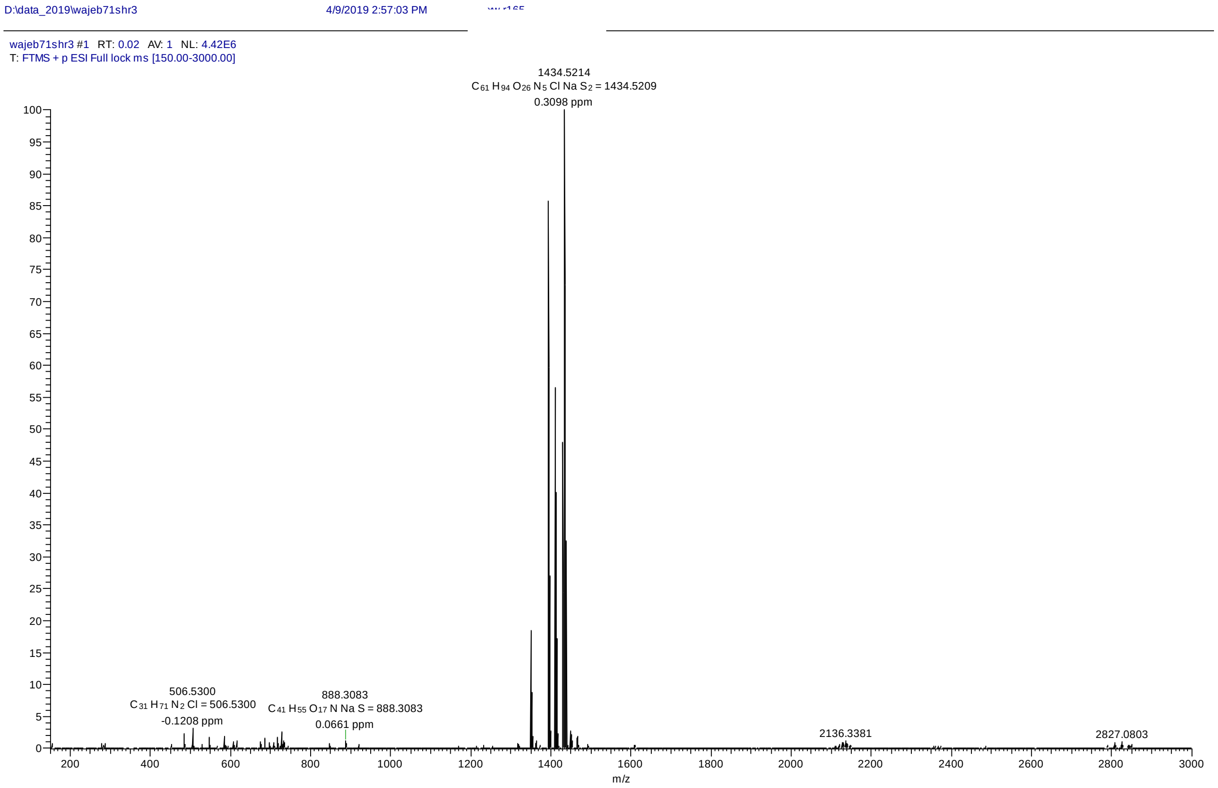

HPLC-UV (254 nm):

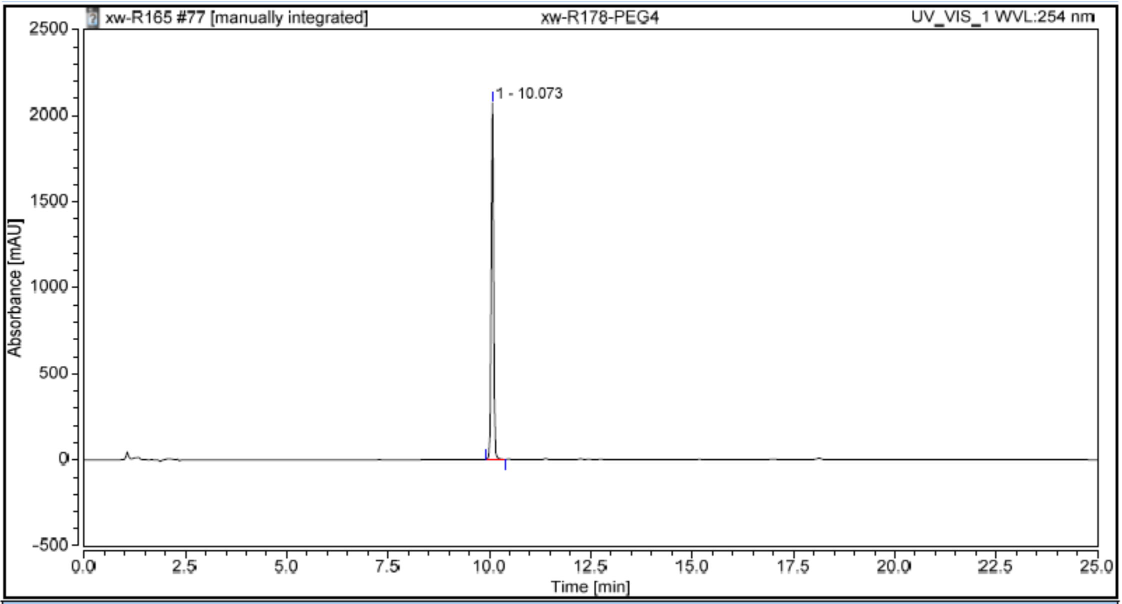

Integration Results

No.	Peak Name	Retention Time min	Area mAU*min	Height mAU	Relative Area %	Relative Height %	Amount n.a.
1		10.073	137.623	2072.594	100.00	100.00	n.a.
**Total:**			**137.623**	**2072.594**	**100.00**	**100.00**	

Trehalose-BODIPY (13)

^{1}H-NMR:

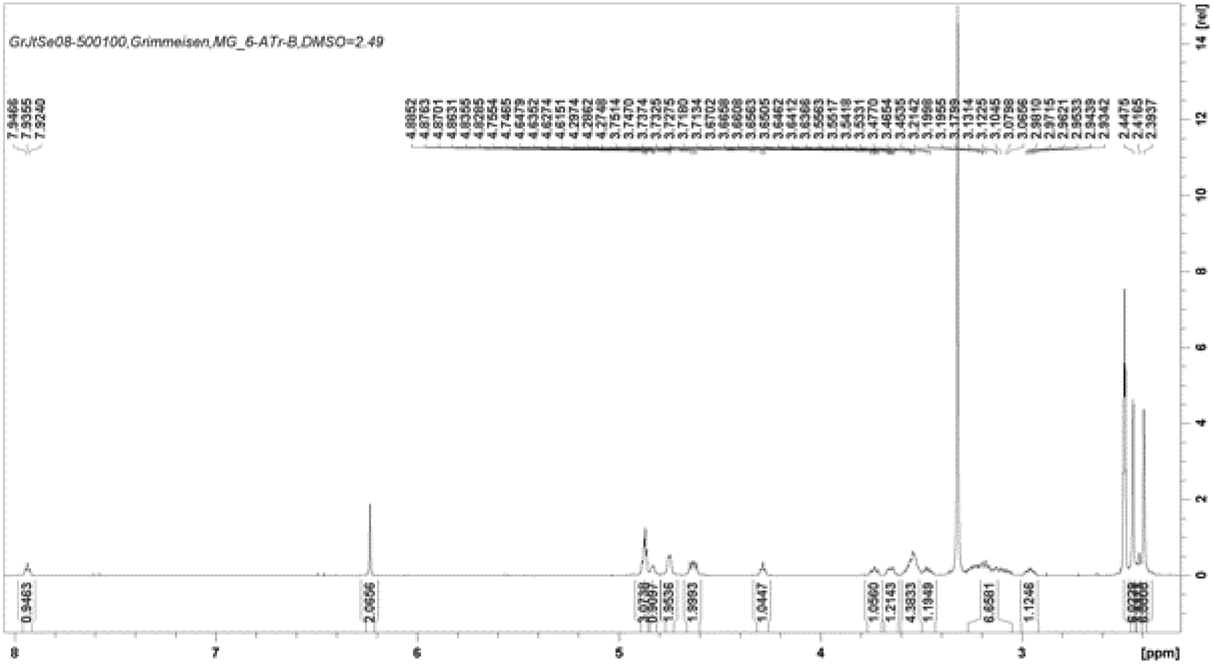

^{13}C-NMR:

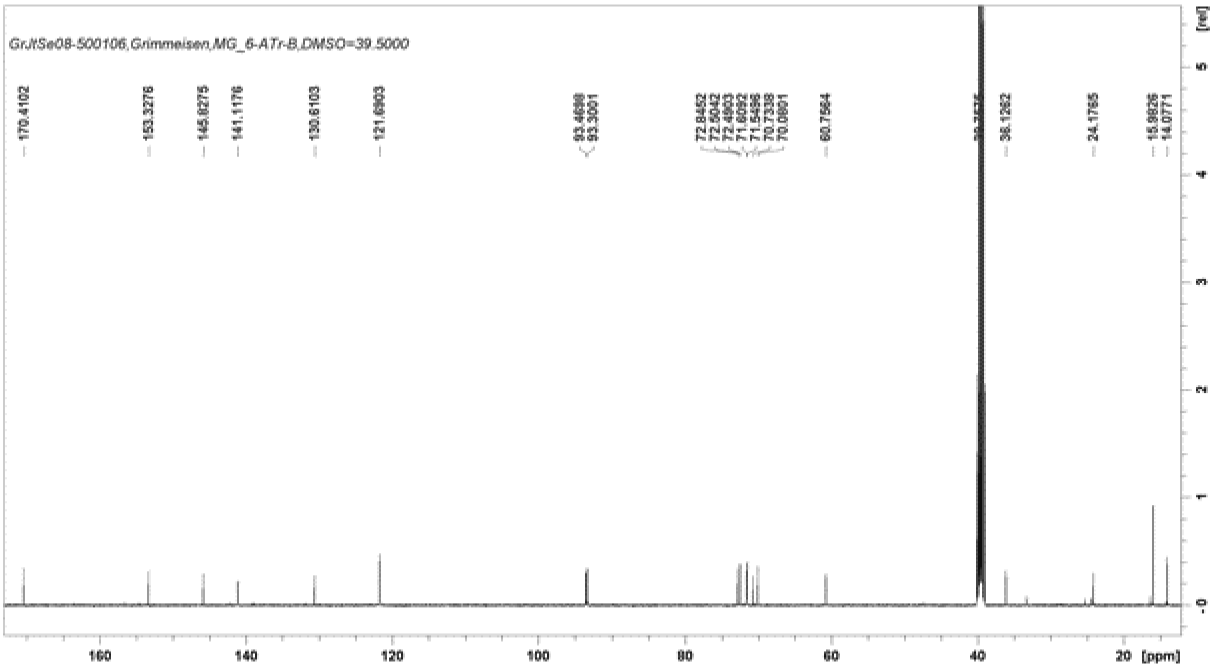

^{11}B-NMR:

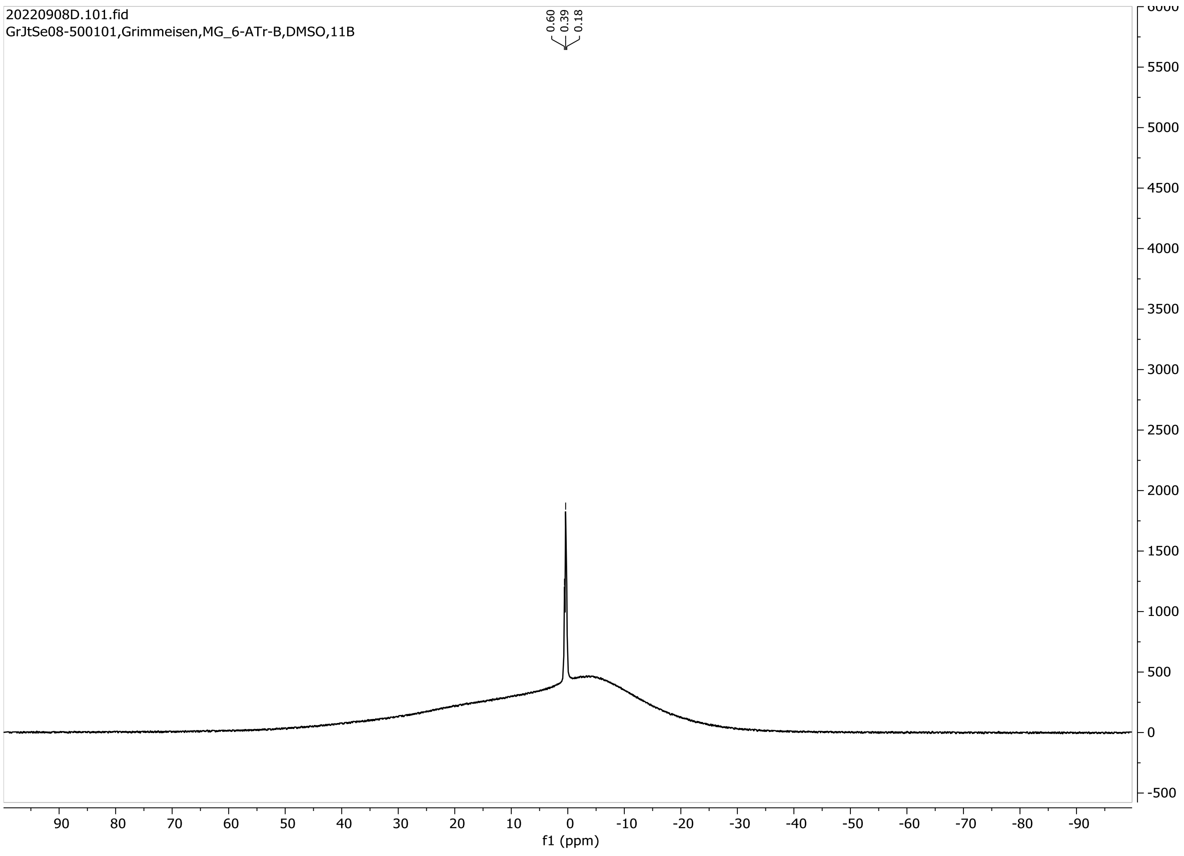

^{19}F-NMR:

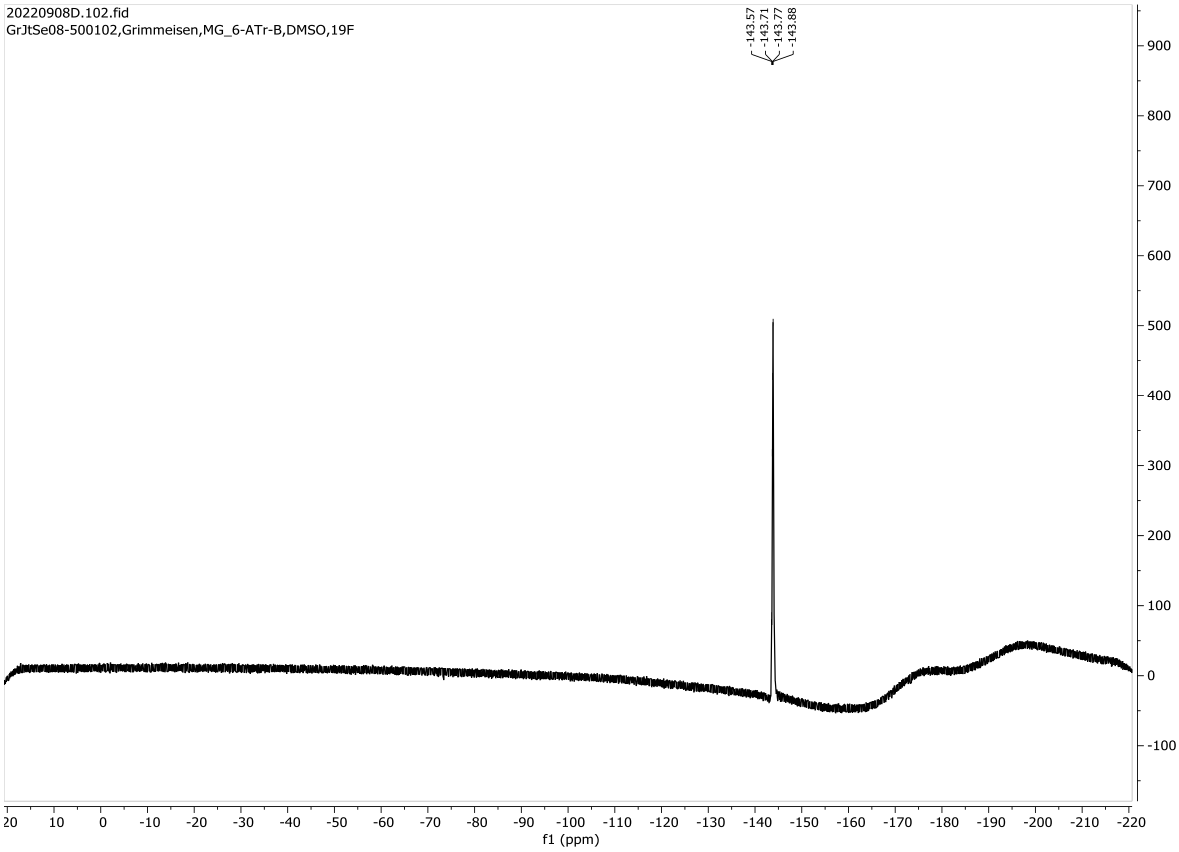

^{1}H- ^{1}H-COSY:

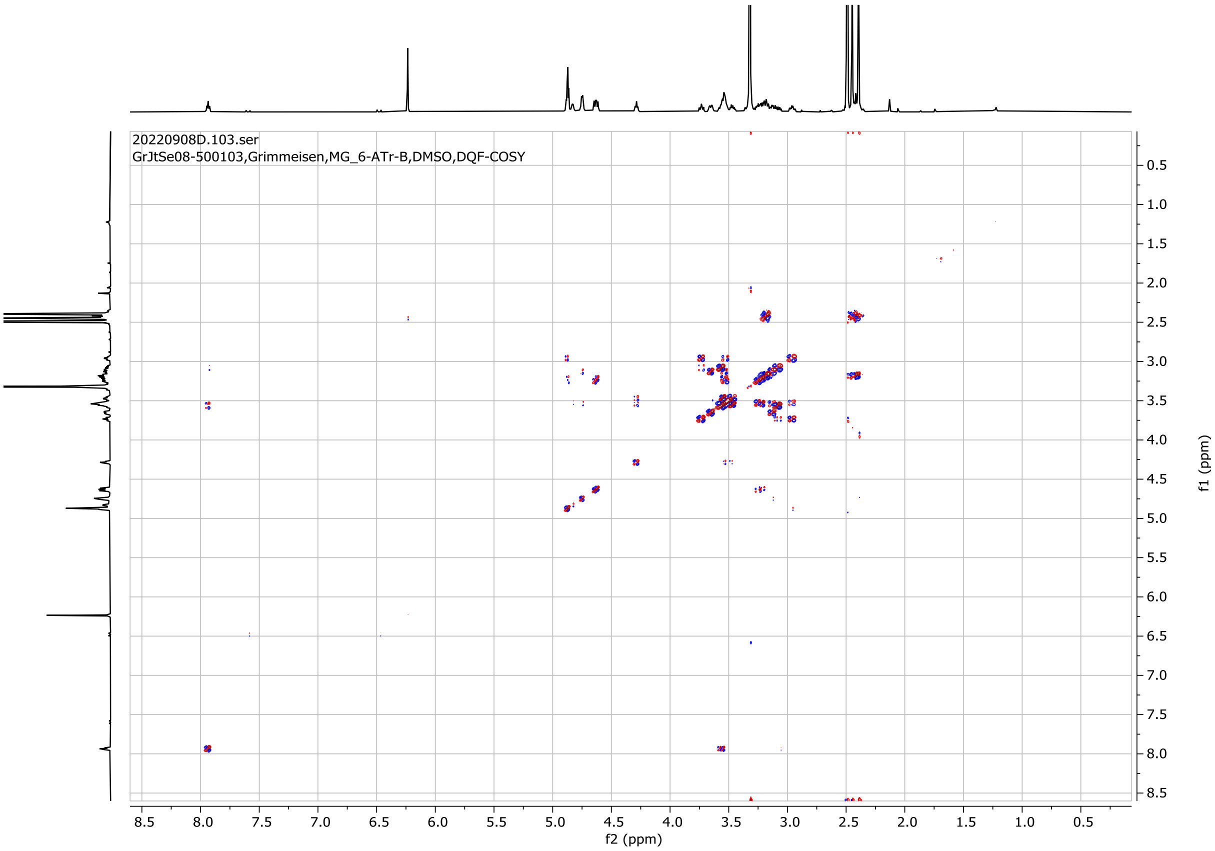

^{1}H- ^{13}C-HSQC:

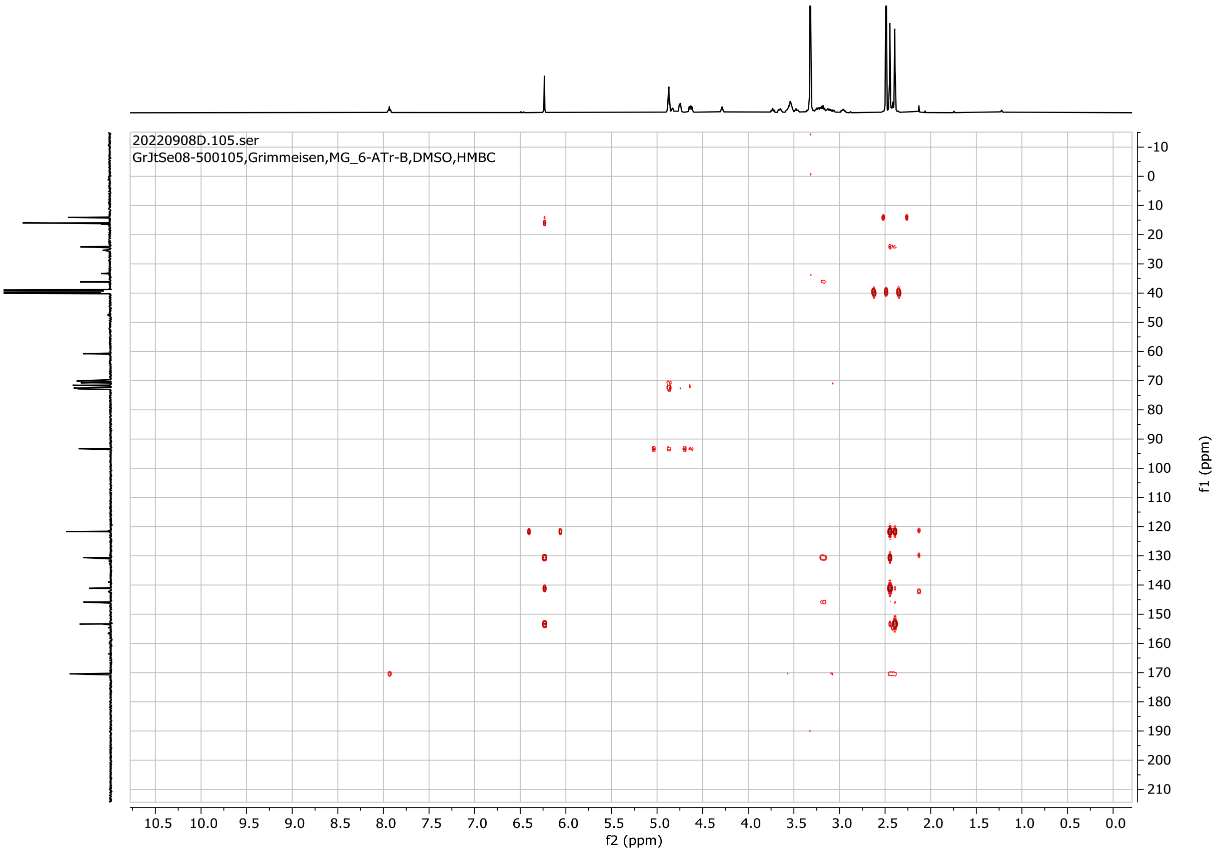

HR-MS:

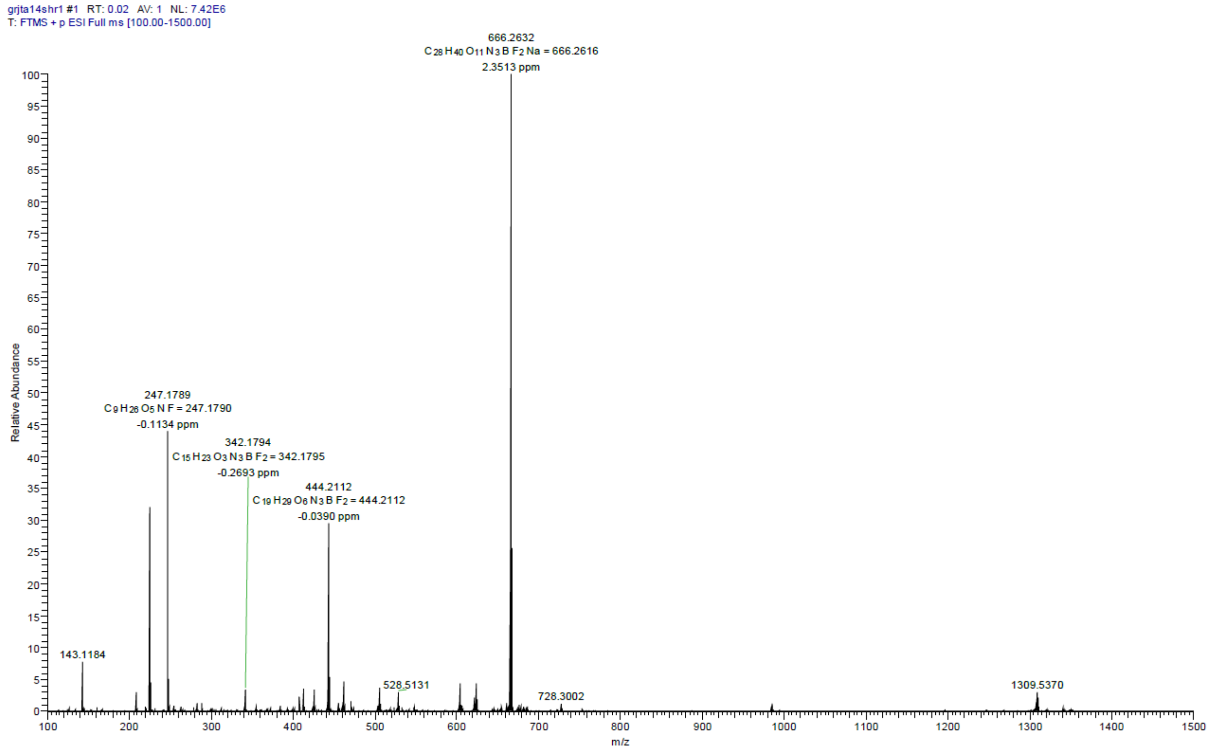

HPLC-UV (254 nm):

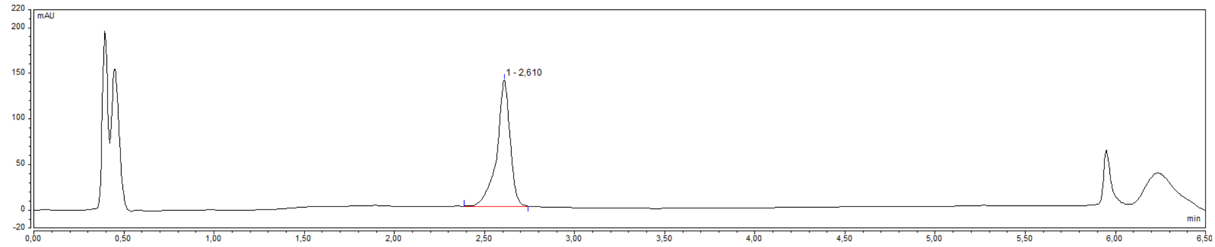

Trehalose-I-BODIPY (1)

^{1}H-NMR:

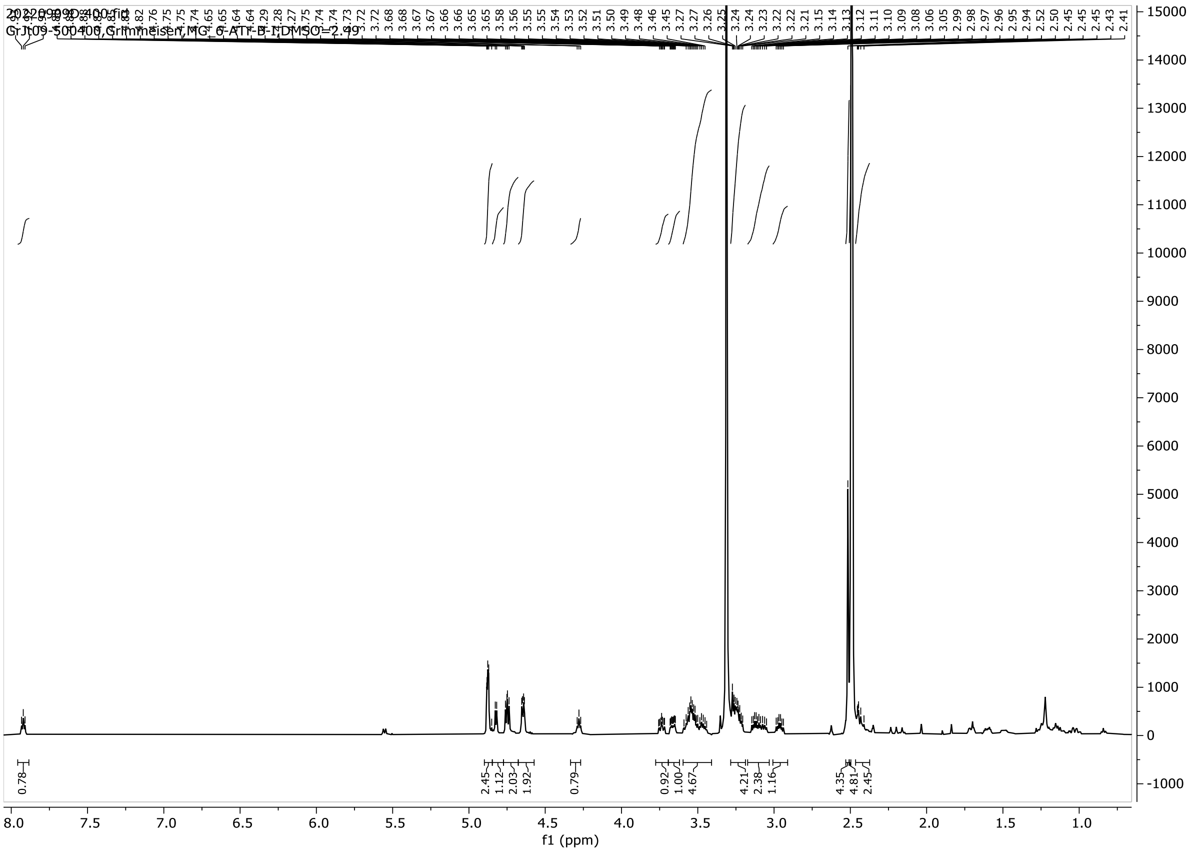

^{13}C-NMR:

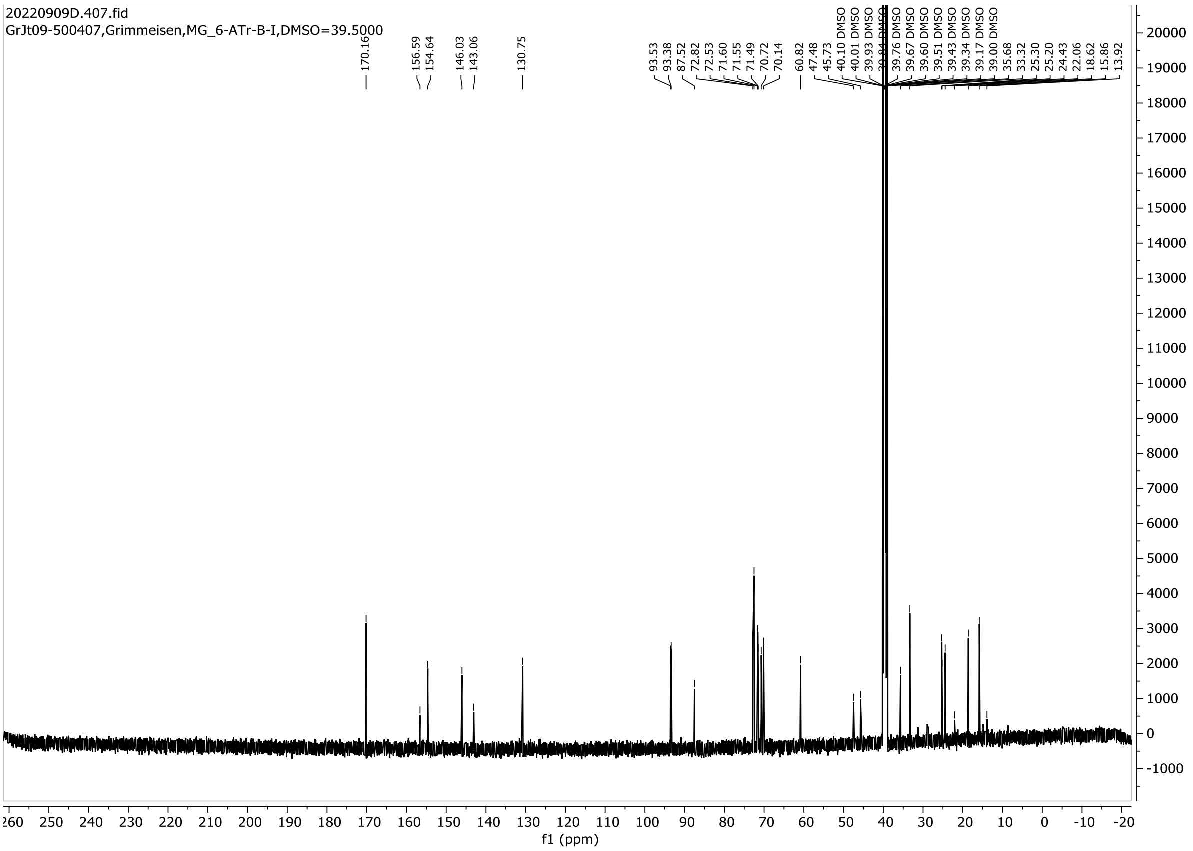

^{11}B-NMR:

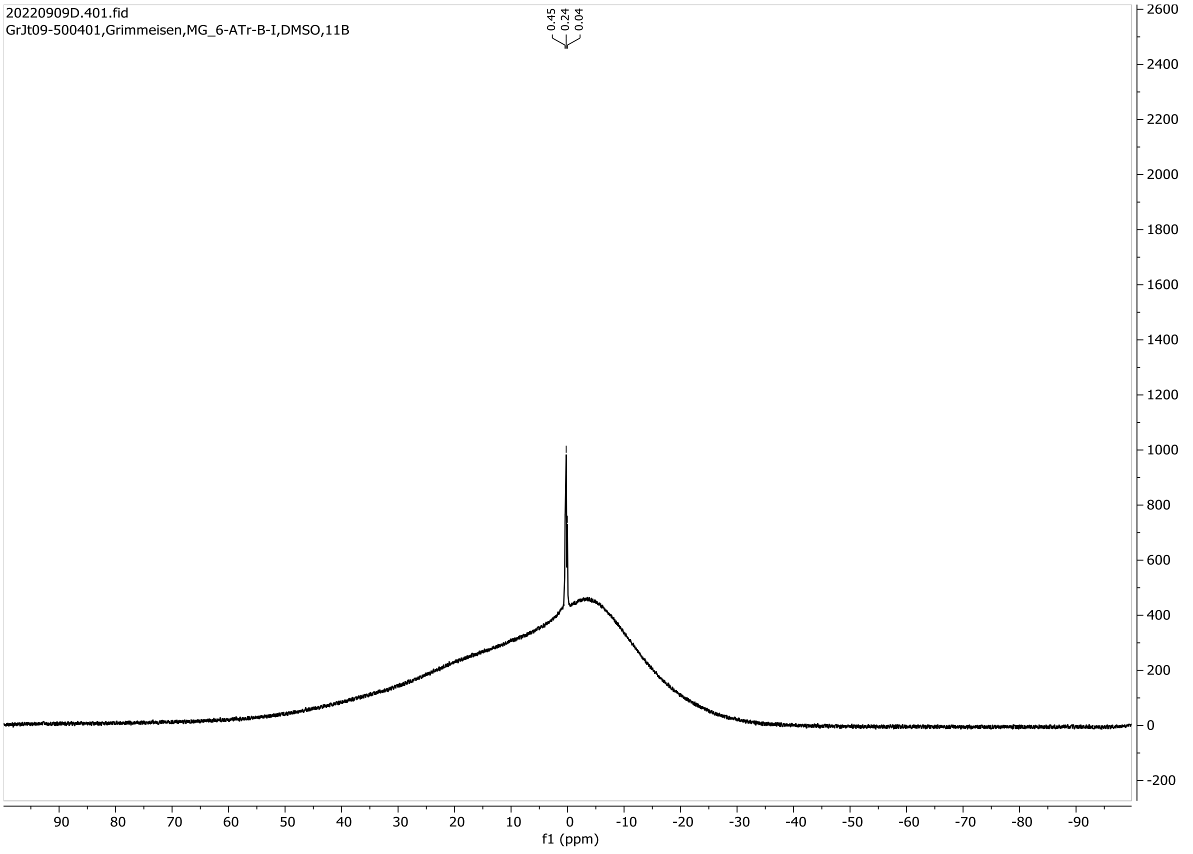

^{19}F-NMR:

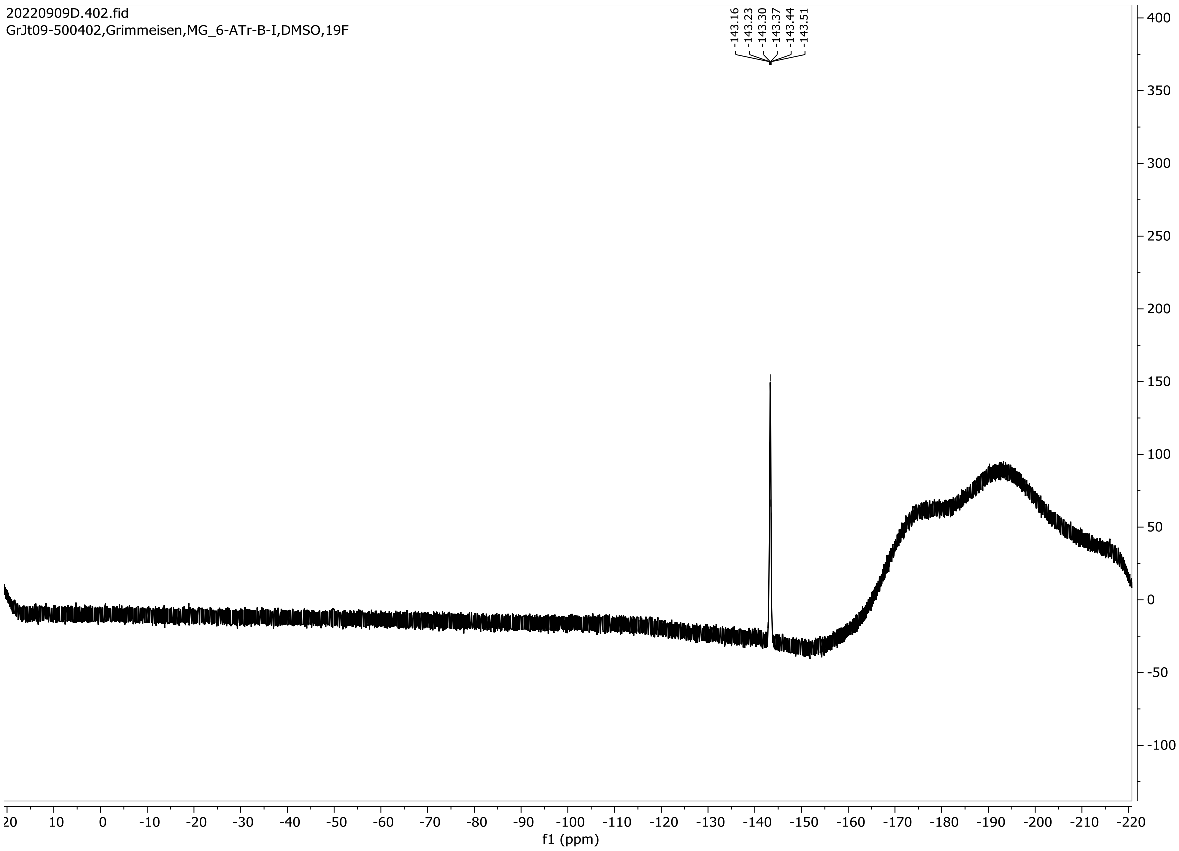

^{1}H- ^{1}H-COSY:

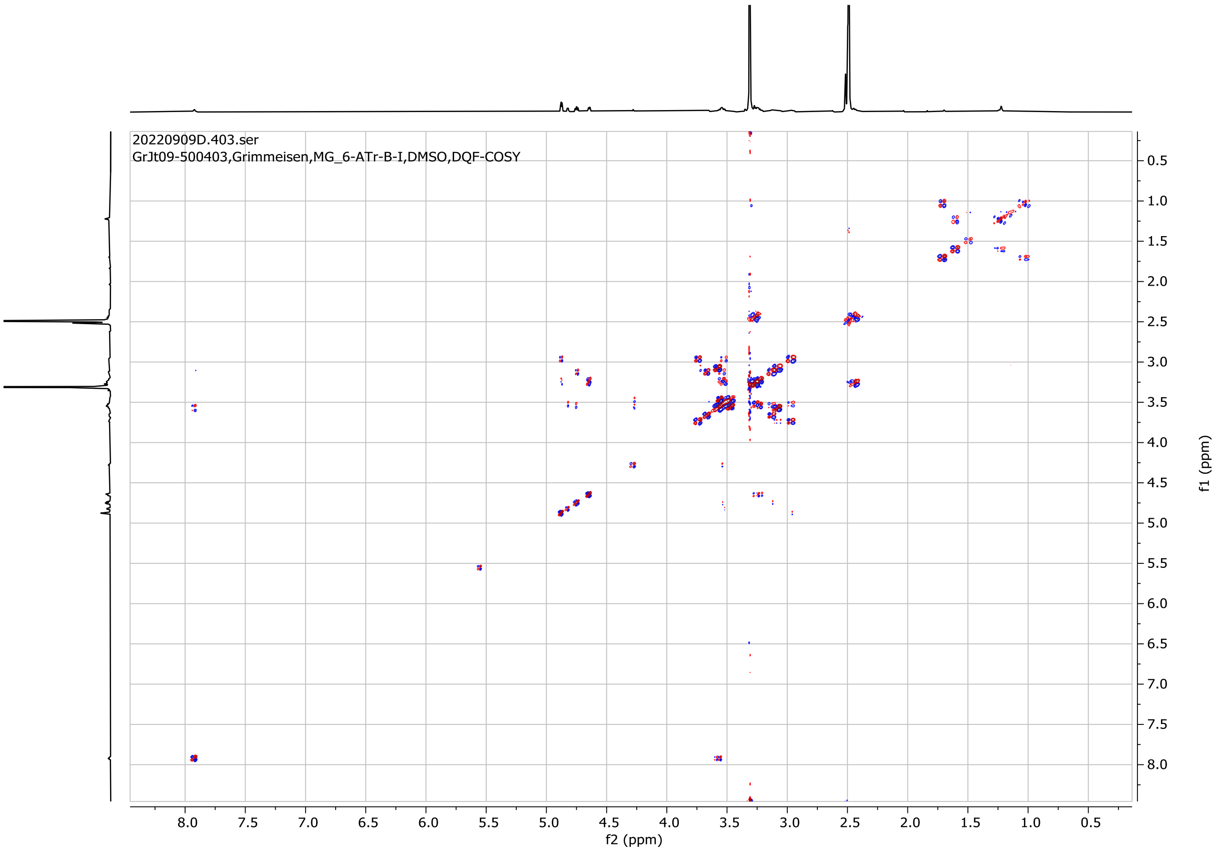

^{1}H- ^{13}C-HSQC:

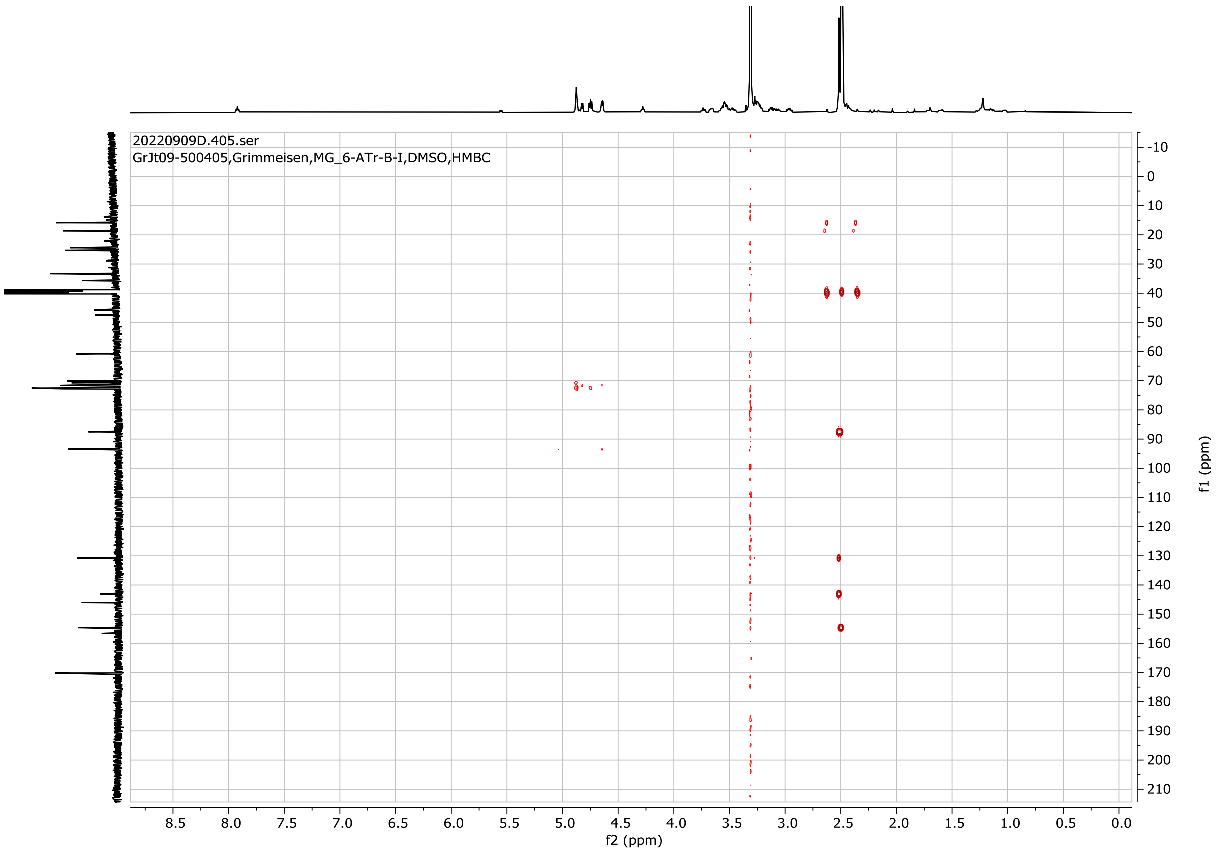

HR-MS:

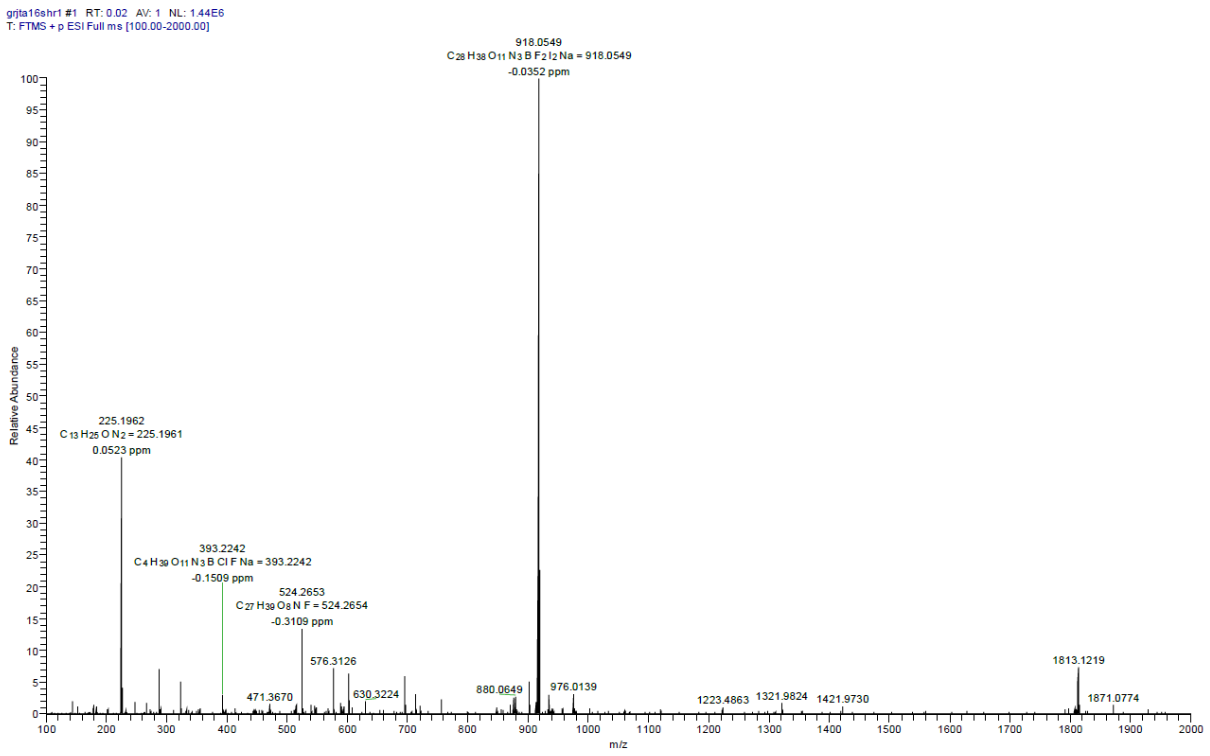

HPLC-UV (254 nm):

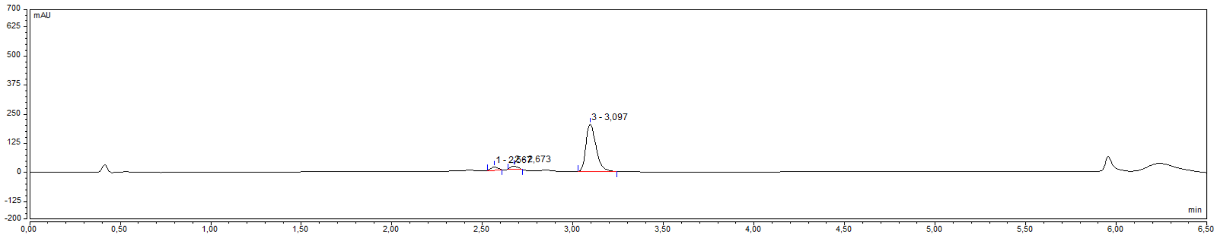

Ag85 processing assay (Figure S4):

Mass of spot (a):

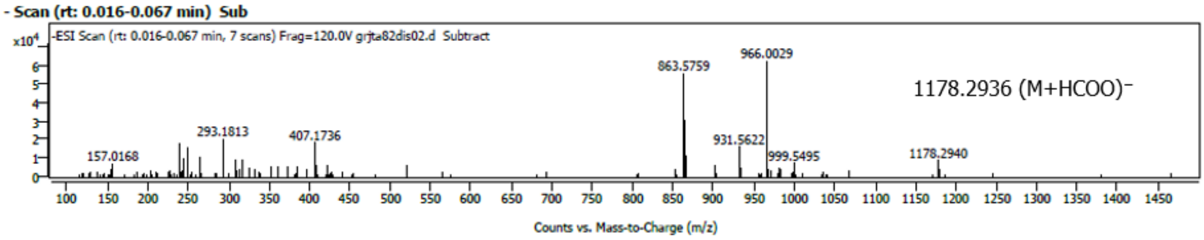

Supplement: Supplementary file 1 — Supplementary Material [file CBIC-26-e202500390-s001.pdf]
